# Combined administration of catalpol, puerarin, gastrodin, and borneol modulates the Tlr4/Myd88/NF-κB signaling pathway and alleviates microglia inflammation in Alzheimer’s disease

**DOI:** 10.3389/fphar.2024.1492237

**Published:** 2024-10-31

**Authors:** Huijing Ren, Ling Tang, Zhiying Yuan, Yang Liu, Xuejiao Zhou, Xiao Xiao, Xingyu Wu, Weihai Chen, Yi Chen, Hongjin Wang, Qiang Xue, Xiaoyu Xu

**Affiliations:** ^1^ College of Pharmaceutical Sciences and Chinese Medicine, Southwest University, Chongqing, China; ^2^ Tongren Polytechnic College, Tongren, Guizhou, China; ^3^ Shapingba District People’s Hospital of Chongqing, Chongqing, China; ^4^ Faculty of Psychology, Southwest University, Chongqing, China; ^5^ Chongqing Medical and Pharmaceutical College, Chongqing, China; ^6^ Southwest University Hospital, Chongqing, China; ^7^ Chongqing Key Laboratory of New Drug Screening form Traditional Chinese Medicine, Chongqing, China; ^8^ Key Disciplines of Traditional Chinese Medicine of Chongqing City, Rehabilitation Medicine of Southwest University, Chongqing, China

**Keywords:** AD, TCM, 3D-NUV, TLR4/MyD88/NF-κB, transcriptome

## Abstract

Alzheimer’s Disease (AD) is a progressive neurodegenerative disorder affecting millions of people worldwide, with no effective treatment currently available. In recent decades, various traditional Chinese medicines (TCMs) and their active ingredients have shown the potential to attenuate the pathogenesis of AD in cellular and animal models. However, the effects of TCM formulas, which are typically administered in practice, have been less studied. This study aims to investigate the therapeutic effects of several formulas consisting of 4 components herbal components: catalpol, puerarin, gastrodin, and borneol, on streptozotocin (STZ)-induced AD models in cells and rats. The new object recognition (NOR), elevated plus maze (EMP), and Morris water maze (MWM) tests were used to evaluate the cognitive functions of rats. Golgi staining, Haematoxylin and Eosin (HE) staining, and Nissl staining analyses were employed assess the physiology of hippocampal tissues. Gene expression profiles were analyzed used transcriptomics and reverse transcription quantitative polymerase chain reaction analysis, while protein expression levels were determined using immunoblotting, immunohistochemical, and immunofluorescence. The production of cytokines was evaluated with enzyme-linked immunosorbent assay. The results demonstrated that the combined administration of these 4 components (CPGB) had superior mitigating effects on AD cell model, as evidenced by the reduced pro-inflammatory cytokine production and decreased deposition of Aβ protein. Further *in vivo* and *in vitro* experiments confirmed that varying doses of CPGB formula effectively ameliorated STZ-induced cognitive deficits, as shown by NOR, MWM, and EMP tests, as well as pathological changes in hippocampal tissues and a 3-dimensional brain neurovascular unit (3D-NVU) model, including decreased deposition of Aβ protein and formation of plaques. Transcriptome sequencing and analysis identified 35 genes with significantly altered expression levels due to STZ and CPGB treatment in hippocampal tissues, which were enriched in the Tlr4/Myd88/NF-κB signaling pathway. Interference with this pathway significantly influenced the therapeutic effects of CPGB in the 3D-NVU model. Collectively, these findings suggest that the combined administration of catalpol, puerarin, gastrodin, and borneol offers superior therapeutic effects on AD by modulating the Tlr4/Myd88/NF-κB signaling pathway. This study strengthens the theoretical foundation for using TCMs to treat AD, proving new insights and references for alleviating and treating AD.

## Introduction

Alzheimer’s disease (AD) is a progressive neurological disorder characterized by dementia, and it is considered one of the most expensive, lethal, and burdensome diseases of this century (Scheltens et al., 2021). According to Alzheimer Disease International, the majority of the 10 million new cases of dementia each year are attributed to AD (Prince et al., 2015). Cohort studies have also reported that the median survival time after an AD dementia diagnosis is less than 6 years (Mayeda et al., 2017; Rhodius-Meester et al., 2019). In recent decades, significant advancements have been made in understanding the pathology of AD, including amyloid beta (Aβ) deposition and plaque (Depp et al., 2023), neurofibrillary tangles of abnormally phosphorylated Tau protein (Wegmann et al., 2021), neuroinflammation, oxidative stress, neuronal loss, synaptic dysfunction (Jack et al., 2024; Scheltens et al., 2021). At the cellular level, alterations in neurons, microglia, and astroglia have been observed to drive the gradual progression of AD before cognitive impairment becomes apparent (De Strooper and Karran, 2016). For example, neuroinflammation in microglia, a group of highly motile phagocytes in the central nervous system frequently found near Aβ deposits in AD patients (Merighi et al., 2022), is recognized as a major component of AD pathology, contributing to disease progression and neurodegeneration (Venegas et al., 2017; Xie et al., 2022). This makes it a popular therapeutic target for AD treatment (Lewcock et al., 2020; Venegas et al., 2017). Specifically, Aβ binds to several innate immune receptors in microglia, including toll-like receptor 2 (TLR2), TLR4, and TLR6, activating the microglia (Liu et al., 2012). Activated microglia then produce elevated levels of pro-inflammatory cytokines such as tumor necrosis factor (TNF)-α, interleukin (IL)-1β, IL-6, nitric oxide (NO), and reactive oxygen species (ROS) (Rani et al., 2023; Thakur et al., 2023), leading to neuroinflammation and AD progression.

Traditional Chinese medicine, including Chinese herbs and their extracts, has been frequently used in the treatment of dementia due to their multi-target and multi-pathway mechanisms, offering promising therapeutical for complex diseases like AD (Chen et al., 2020). Various active ingredients extracted from Chinese herbs have shown beneficial effects on AD through diverse mechanisms. For instance, catalpol, an active compound from *Rehmannia glutinos*, has been reported to slow AD progression by enhancing the expression of neural stem cell exosome-released miR-138-5p (Chen et al., 2022; Meng et al., 2023). Similarly, puerarin, derived from *Puerariae Lobatae Radix*, demonstrates neuroprotective effects by targeting the PI3K/Akt signaling pathway (Wang et al., 2022). Gastrodin, from *Gastrodia elata*, reduces neuro-inflammation in AD models by inhibiting the NF-κB signaling pathway (Yin et al., 2024). Lastly, borneol, extracted from *Blumea balsamifera DC* or *Cinnamomum camphora* (L.) *Presl*, has been found to effectively relieves AD-like symptoms in mice by clearing Aβ aggregates (Ye et al., 2024). These studies highlight the potential of individual herbal ingredients in targeting different aspects of AD pathology.

However, most of these ingredients were administered as single compounds, whereas combination therapies have been shown to produce enhanced therapeutic effects in complex diseases like AD (Cummings et al., 2024; Cummings et al., 2019). Given that TCM traditionally involves the use of multi-ingredient formulations and that AD is a multifactorial disease (Scheltens et al., 2021), it is hypothesized that combining these plant-derived ingredients may offer superior benefits in mitigating AD progression.

We hypothesize that the combined administration of catalpol, puerarin, gastrodin, and borneol will demonstrate improved mitigative effects on AD progression compared to their individual use. To validate our hypothesis, we will formulate these 4 ingredients into different combinations and evaluate their therapeutic effects on AD progression in models. Subsequently, we will further explore the underlying mechanisms of the formulation with the most potent therapeutic effects. Our research aims to further refine the theoretical foundation of using traditional Chinese medicine to treat AD, providing new insights and references for alleviating and treating the disease.

## Materials and methods

### Formulation preparation

In this study, we utilized 4 plant-derived compounds: catalpol (CAS: 2415-24-9), puerarin (CAS: 3681-99-0), gastrodin (CAS: 62499-27-8) and borneol (CAS: 124-76-5). Catalpol, puerarin, and gastrodin were sourced from Dierg Pharmaceutical Technology Co., Ltd., Nanjing, China, while borneol was obtained from Tongrentang, Chongqing, China. Supplementary Figure S1 presents the chemical structure diagrams of these compounds. In our previous research, we discovered that a combination of catalpol, puerarin, and borneol in a 1:4:2 ratio maximized brain absorption and exhibited neuroprotection effects in an ischemic model (Qiang et al., 2016; Tang et al., 2016). Building on these findings, we incorporated gastrodin into the formulation, using a 1:4:2:2 ratio for CPGB, which yielded significantly improved results in AD models compared to other ratios (data not shown). For this optimized ratio, we prepared 15 formulations: catalpol alone (C), puerarin alone (P), gastrodin (G), borneol (B), catalpol + puerarin (CP), catalpol + gastrodin (CG), catalpol +borneol (CB), puerarin +gastrodin (PG), puerarin + borneol (PB), gastrodin + borneol (GB), catalpol + puerarin + gastrodin (CPG), puerarin + gastrodin + borneol (PGB), catalpol + gastrodin + borneol (CGB), catalpol + puerarin + borneol (CPB), catalpol + puerarin + gastrodin + borneol (CPGB). These formulations were prepared using a 35% hydroxypropyl-beta-cyclodextrin solution (Merck, Kenilworth, NJ, United States), which was commonly used to solubilize poorly soluble drugs due to its ability to form inclusion complexes with hydrophobic molecules (Wang and Brusseau, 1993). Specifically, 800 mg of puerarin and 400 mg of borneol were dissolved in 50 mL of the solution, followed by the addition of 200 mg of catalpol and 400 mg of gastrodin to create the high-dose CPGB solution (360 mg/kg). The solution was subsequently diluted to obtain mild (180 mg/kg) and low (90 mg/kg) doses. All these formulations were freshly prepared and used immediately, and no changes in the physical appearance of the solution were observed during the preparation or usage process. As a result, a stability study on these formulations was not conducted in this study. Additionally, we used donepezil hydrochloride tablets (DPN; Zein Bio, Nanjing, China) as a positive control to evaluate the therapeutic effects of these formulations.

### Cell culture and treatment

The pheochromocytoma cell line (PC12) and the BV2 microglial cell line (BV2) used in this study were kindly provided by Prof. Hongyi Qi from the College of Pharmacy, Southwest University, Chongqing, China. These cell lines were cultured in a complete medium composed of 89% Dulbecco’s Modified Eagle Medium: Nutrient Mixture F-12 (DMEM/F12; Gibco, Waltham, MA, United States), 10% fetal bovine serum (FBS; Gibco, Waltham, MA, United States), and 1% penicillin-streptomycin (PS; Gibco, Waltham, MA, United States) maintained at 37%°C in an incubator with 5% CO_2_. Once the cells reached 90% confluence, they were detached, collected, and resuspended in a complete medium for subsequent experiments.

### AD cell model establishment and treatment

Referencing previous studies (Gupta et al., 2018; Kamat, 2015; Li et al., 2020), we established an AD cell model by treating PC12 and BV2 cells with 0.5 mol/mL STZ. Briefly, PC12/BV2 cells were evenly seeded into 6-well plates (ThermoFisher, Shanghai, China) at a density of 2 × 10^5^ cells each well. After 24 h of proliferation, the supernatant was discarded, and a fresh complete medium containing either no additives, 0.5 mol/mL streptozotocin (STZ; Solarbio, Beijing, China) or 0.5 mol/mL STZ plus various formulations was added for further culture. Each treatment was performed with 3 replicates. Following cultivation, the supernatant was collected for subsequent enzyme-linked immunosorbent assay (ELISA) experiments, while cells from each well were collected for total RNA or total protein extraction.

Alternatively, PC12/BV2 cells were seeded onto sterile coverslips in 24-well plates (ThermoFisher, Shanghai, China) at a density of 5 × 10^4^ cells/mL, with 1 mL of cell suspension added to each well. After 24 h of proliferation, the supernatant was discarded, and a fresh complete medium containing either no additives, 0.5 mol/L STZ, or 0.5 mol/L STZ plus various formulations was added for further culture. Each treatment was performed with 3 replicates. After cultivation, the coverslips were collected for subsequent immunofluorescence analyses.

### Three-dimensional brain neurovascular unit (3D-NVU) model establishment and treatment

To establish the 3D-NVU model, hippocampal neural stem cells (NSCs) and brain microvascular endothelial cells (BMECS) were harvested according to previous established methods (Liu et al., 2013; Wang et al., 2021). Briefly, NSCs were isolated from the hippocampi of 24-h-old SD rat pups. After anesthesia and decapitation, the brains were quickly removed under sterile conditions and placed in cold D-Hanks solution (Solarbio, Beijing, China). The hippocampal region was dissected, minced, and centrifuged at 800 rpm for 5 min. The resulting tissue was digested with accutase (ThermoFisher, Shanghai, China) for 5 min at 37°C. The cell suspension was filtered and centrifuged. The pellet was resuspended in DMEM/F12 medium containing 20 ng/mL epidermal growth factor (EGF), 10 ng/mL basic fibroblast growth factor (bFGF), 2% B27, and 0.0002% heparin, then cultured at 37°C with 5% CO2. Cells were passaged or used for experiments after 6-7 days. BMECs were isolated from the cortex of 10-day-old SD rats. After decapitation and removal of the brain, the cortex was dissected, and large blood vessels and meninges were removed. The tissue was homogenized, followed by centrifugation at 1,200 rpm for 5 min. The homogenate was treated with 25% BSA and centrifuged at 2,500 rpm to collect the microvascular fragments. The fragments were digested with 0.1% type II collagenase for 5 min, then centrifuged and resuspended in complete medium with 20% FBS. Cells were seeded into collagen-coated flasks and cultured at 37°C with 5% CO2. Media were changed every 2-3 days, and cells were used when 90% confluence was reached.

NSCs and BMECs were mixed with Matrigel (Corning, Corning, NY, United States) at a 1:10 density ratio. The mixture was resuspended, and 150 μL of the cell/Matrigel mixture was evenly distributed at the bottom of a laser confocal dish and incubated to solidify. After 20 min of incubation, an additional 50 μL of the cell/Matrigel mixture was spread over the surface of the solidified layer and returned to the incubator for another 20 min before adding the medium. The NSC/BMEC co-culture medium consisted of DMEM/F12 and Neurobasal (ThermoFisher, Shanghai, China) in a 1:1 ratio, supplemented with 2% B27, 20 ng/mL recombinant human EGF (rhEGF), and 10 mg/mL recombinant human basic fibroblast growth factor (rhbFGF). On the 3rd day, the B27 supplement was switched to a formulation containing vitamin A. After 7 days of culture, the 3D-NVU model was successfully replicated, as confirmed by the positive expression of β-tubulin3, glial fibrillary acidic protein (GFAP), and CD31 marker proteins (Wang et al., 2021). After confirming the successful establishment of the 3D-NVU model, a medium containing either no additives, 0.5 mol/L STZ, 0.5 mol/L STZ plus various formulations, or 0.5 mol/L STZ + various formulations + inhibitors were added for further culture. Each treatment was performed with 3 replicates. Following cultivation, the supernatant was collected for subsequent ELISA experiments, while cells from each well were collected for total RNA or total protein extraction.

### BV2 conditioned medium collection and treatment

Referencing a previous study (Qureshi et al., 2022), a conditioned medium of microglia was employed to induce cellular damage. Briefly, BV2 cells were evenly seeded into 6-well plates at a density of 2 × 10^5^ cells per well. After 24 h of culture, the medium in each well was replaced with a fresh complete medium containing either no additives, 0.5 mol/L STZ, or 0.5 mol/L STZ plus various formulations. Following another 24 h of culture, the medium was discarded, and the cells were washed 3 times. Subsequently, 1 mL of completing medium was added to each well for an additional 24 h of culture. The medium in each well was then collected as a conditioned medium. Additionally, PC12 cells were seeded into 96-well plates at the density of 2 × 10^3^ per well. After 24 h of culture, the medium was discarded, and the collected conditioned medium from BV2 cells was added to the wells for an additional 24 h of culture. The viability of the PC12 cells was then assessed using the Cell Count Kit-8 (CCK8) assay. Briefly, the supernatant was discarded, and 100 μL of fresh complete medium containing 10% CCK-8 solution (Solarbio, Beijing, China) was added. After 2 h of incubation, the optical density at 450 nm (OD450) was measured using a microplate reader (ThermoFisher, Shanghai, China).

### Animal housing and management

The rats used in this study were male Sprague-Dawley rats of specific pathogen-free grade, aged 3–4 months with weights ranging from 350 to 380 g. They were purchased from Liaoning Changsheng Biotechnology Co., Ltd. (Shenyang, China). The rats were maintained at the Experimental Animal Center of the College of Pharmacy, Southwest University. They were housed in standard cages at 22°C ± 2°C, on a 12-h light/dark cycle, with a humidity level of 40%–60%. Throughout the experimental period, all rats had free access to water and food, and the bedding was refreshed every 2 days.

### AD rat model establishment

Following a previous study (Moreira-Silva et al., 2018), an AD rat model was established through intracerebroventricular (ICV) injection of STZ. Briefly, after 1 week of adaptive feeding and a subsequent 12-h fasting period, the rats were anesthetized with 5% isoflurane. Their heads were then secured in a stereotaxic apparatus, and anesthesia was maintained with 2% isoflurane. The fur on the scalp was trimmed, and the skin was disinfected with iodine and alcohol. A midline incision approximately 1.5 cm in length was made along the sagittal suture to expose the skull. The periosteum was washed with 3% hydrogen peroxide (Solarbio, Beijing, China) and pushed aside to fully expose the cranial bone. Bilateral burr holes were drilled based on coordinates referenced from the bregma: −0.8 mm anteroposterior, ± 1.4 mm mediolateral, and −3.6 mm dorsoventral, according to the rat brain atlas by Paxinos and Watson (Paxinos and Watson, 2006). Small burr holes were carefully drilled using a dental drill, thinning the skull without penetrating it. A microsyringe (Hamilton, Reno, NV, United States) was then carefully inserted vertically to a depth of 3.8 mm. Afterward, both STZ and arachidonoylethanolamide (AEA; Sigma-Aldrich, St. Louis, MO, United States) were prepared in citrate buffer (Merck, Kenilworth, NJ, United States) and administered using an automated microinjection system (Insight Equipment LTDA, São Paulo, Brazil). This system included a polyethylene microtube connected to an injection needle and the inserted microsyringe coupled with an infusion pump. Each rat received 2 ICV bilateral injections of 2 μL at a rate of 1 μL/min. The first injection consisted of either citrate buffer (for sham procedure; 0.05 M) or AEA (100 ng). Five minutes later, a second injection of either citrate buffer or STZ (2 mg/kg) was administered. To prevent reflux, the injection needle was left in place for at least 2 min following each infusion. Post-surgery, the rats received an intraperitoneal injection of ceftriaxone sodium (100 mg/kg; Merck, Kenilworth, NJ, United States) once daily for 3 days to prevent infection, along with analgesic carprofen (5 mg/kg; Merck, Kenilworth, NJ, United States). Additionally, 2 mL of saline was administered subcutaneously for rehydration. The rats were then kept on thermal blankets with controlled temperature until full recovery.

### Treatment of rats and sample collection

On the day following surgery, rats in both the sham and model groups were orally administered a 35% hydroxypropyl β-cyclodextrin solution. Meanwhile, those in the treatment groups received DPN and CPGB at doses of 90, 180, and 360 mg/kg for low, medium, and high doses, respectively, via gavage at a rate of 1 mL/kg. Each group consisted of 16 rats. The treatment was administered daily for 42 consecutive days. The doses of CPGB were determined referencing to our previous research (Tang et al., 2016). An earlier acute toxicity study confirmed that these doses did not produce any observable toxic effects on the major organs of the rats. In summary, rats administered 5.12 g/kg of CPGB exhibited no abnormal reactions compared to normal rats. In contrast, rats given 6.4 g/kg of CPGB exhibited mild symptoms, such as reduced activity and partial paralysis, which resolved within 24 h. Furthermore, a 14-days treatment with CPGB at doses of 5.12 g/kg or lower did not result in any signs of toxicity, mortality, or significant changes in behavior, body weight, or organ function. Behavioral assessments were performed using the Novel Objective Recognition (NOR) test from days 31–33, the Elevated Plus Maze (EPM) test from days 34–35, and the Morris Water Maze (MWM) test from days 46 to 36. Upon completing the MWM test on the 42nd day, 3 rats from each group were euthanized to collect hippocampal electrical signals. The remaining rats were anesthetized and euthanized by cervical dislocation, after which their brains were collected for further experiments.

### New object recognition

To evaluate the hippocampus-dependent non-emotional memory in rats, the NOR test was conducted from days 31–33, as referenced in a previous study (Leger et al., 2013). The experimental setup consisted of a cylindrical enclosure made of black Plexiglas plastic, measuring 50 cm in both diameter and height. To minimize anxiety due to unfamiliarity, rats were allowed to acclimate to the environment by being placed in the apparatus for 5 min, 24 h before the test. During the familiarization phase, 2 identical objects, labeled A1 and A2, were positioned at opposite corners of the enclosure, each 5 cm from the corner. Each rat was gently placed in the enclosure and allowed to explore the objects for 5 min. After a 24-h interval, object A2 was replaced with a new object for the test session, and the rat was reintroduced to the enclosure to explore the objects for another 5 min. Exploratory behavior was defined as orienting the nose towards an object at a distance of less than 2 cm or making physical contact with the object using the nose. The exploration time for each object during both the familiarization and test sessions was recorded. The Discrimination Index (DI) was calculated using the following formula: DI = Time spent in exploring the novel object/(Time spent in exploring old object + Time spent in exploring the novel object).

### Elevated plus maze

To investigate anxiety-related behavior in rats, the EMP test was conducted, referencing a previous study (Walf and Frye, 2007). The maze consisted of 1 open arm and 1 closed arm. On the 34th after surgery, each rat was placed facing outwards on the open arm for free exploration. The time taken for the rat transition from the open arm to its initial entry into the closed arm was recorded by a camera and noted as the initial escape latency (EL1). If this time exceeded 90 s, it was recorded as 90 s. On the 35th day, the same test method was used to obtain the ultimate escape latency (EL2). The relative escape latency (REL) was calculated using the following formula: REL = EL2/EL1 × 100%.

### Morris water maze test

The spatial learning and memory abilities of rats were assessed using the Morris Water Maze (MWM) experiment (Vorhees and Williams, 2006). The procedure involved a series of acquisition trials conducted over 5 consecutive days. Each day, the rats underwent 4 trials with a minimum inter-trial interval of 15 s. In each trial, rats were released from a randomly selected starting point and given up to 120 s to locate the platform. Upon finding the platform, they were allowed to remain on it for 5 s. If a rat failed to find the platform independently, it was manually guided to the platform and held there for 15 s. The latency times for all trials were recorded. On the 6th day, a probe trial was conducted to evaluate spatial memory without the platform. During this trial, the frequency of platform crossings, as well as the duration and distance traveled within the target quadrant, were measured. The EthoVisionXT 8.5 software (Noldus, Wageningen, Netherlands) was used to analyze the frequency of each rat passing through the location of the original platform, the swimming distance within the quadrant, and the time spent in the platform area. More crossings and longer routes indicated better learning and memory retention, as did longer durations spent in the quadrant containing the original platform.

### Hippocampal electrical signal acquisition test

After the MWM test, 3 randomly selected rats were anesthesia with 5% isoflurane, and their heads were secured on a brain stereotaxic instrument (Insight Equipment LTDA, São Paulo, Brazil). Recordings were made using a NeurLynx 32-channel electrophysiological recorder. An 8-channel recording electrode was implanted with coordinates relative to the brain Bregma point as the zero point: dorsoventral at −3.6 mm and lateral ventricle at 2.5 mm. Following a 30-min baseline recording, the spike peak potential was recorded for an additional 30-min.

### ELISA

ELISA was employed to determine the concentrations of Aβ1-42, TNF-α, IL-1β, and IL-6 in the hippocampal tissue homogenate or collected supernatant using commercialized ELISA kits (Nanjing Jiancheng, Nanjing, China) according to their instructions. In brief, 100 μL of supernatant was added to each well of the reaction plate, which was then sealed with a membrane and incubated at room temperature for 2 h. Subsequently, biotinylated antibody, horseradish peroxidase-labeled streptavidin, and tetramethylbenzidine solution were added to each well. The plate was incubated overnight at room temperature in the dark. Finally, 50 μL of stop solution was added to terminate the reaction, and the OD450 was measured using a microplate reader.

### RNA extraction and reverse transcription quantitative polymerase chain reaction (TR-qPCR) analysis

Total RNA from collected hippocampal tissue and cells was extracted using Trizol Reagent (TransGen, Beijing, China) following the manufacturer’s protocol. The concentration and purity of extracted RNA were measured using a NanoDrop ND-2000 spectrophotometer (Thermo Fisher Scientific, Wilmington, NC, United States), and its integrity was confirmed through 1% agarose gel electrophoresis. Following this, the total RNA samples were reverse transcribed to complementary DNA (cDNA) using the RevertAid First Strand cDNA Synthesis Kit (ThermoFisher, Shanghai, China) following the manufacturer’s instructions. RT-qPCR was then conducted under the following conditions: initial denaturation at 95°C for 30 s, followed by 40 cycles of denaturation at 95°C for 5 s, and annealing and extension at 60°C for 30 s. The primer sequences used in this study are listed in Supplementary Table S1, and the data was analyzed using the 2^ΔΔC^ method in Excel.

### Total protein extraction and immunoblotting analysis

Proteins were extracted from collected hippocampal tissue and cells using RIPA lysis buffer (Solarbio, Beijing). The protein concentrations were determined with a BCA kit (Beyond, Shanghai, China). Subsequently, the proteins were denatured at 100°C for 10 min. And 30 μg of the extracted total protein from each sample were loaded onto a 12% sodium dodecyl sulfate-polyacrylamide for electrophoresis. After electrophoresis, the proteins were transferred onto a polyvinylidene fluoride membrane. The membrane was blocked with 5% skim milk in TBST for 2 h. Following blocking, the membranes were incubated overnight at 4°C with primary antibodies. On the following day, the membranes were treated with a secondary antibody and enhanced chemiluminescent reagents to visualize the protein bands. The antibodies used in this study are listed in Supplementary Table S2. The intensity of the protein bands was analyzed using ImageJ software.

### Histological analysis

For Golgi staining, a Golgi staining kit (Servicebio, Chengdu, China) was employed. Briefly, the brain tissues collected were first washed and fixed with 4% paraformaldehyde (Life iLab Bio, Shanghai, China) at −20°C for 24 h. Following fixation, the tissues were immersed in a dark A + B mixture at room temperature for 14 days, then soaked in C solution for an additional 5 days. The tissues were subsequently sectioned using a freezing microtome set to a thickness of 180 μm. These sections were stained with D solution and observed under a microscope for imaging.

The remaining freshly collected brain tissues were fixed in 4% paraformaldehyde for 48 h. After fixation, the tissues were rinsed several times with tap water, dehydrated through a gradient alcohol series, soaked in paraffin, and then embedded. For Haematoxylin and Eosin (HE) staining analysis, the paraffin sections (5 μm thick) were incubated at 80°C for 20 min, de-paraffinized, and then soaked in haematoxylin solution (Solarbio, Beijing, China) for 15 min. The sections were rinsed with tap water for re-bluing, stained in eosin, hydrated through a gradient alcohol series, cleared with xylene, and mounted with neutral resin before being air-dried, observed, and photographed under a microscope.

For Nissl staining, a Nissl staining kit (Beyotime, Shanghai, China) was employed. Briefly, brain sections were dewaxed, stained with methylene blue solution for 10 min, differentiated for 1 min, treated with ammonium molybdate solution for 5 min, and sealed using a neutral gum. The Nissl bodies were then visualized and imaged using a fluorescence microscope (Olympus, Tokyo, Japan) and quantified with ImageJ software.

For Immunohistochemical staining, the sections were dewaxed, washed 3 times, immersed with 3% H_2_O_2_ at room temperature for 10 min, and incubated with antigen retrieval solution at 37°C for 20 min. The sections were blocked with 5% bovine serum albumin (ThermoFisher, Shanghai, China) at room temperature for 2 h. Following blocking, sections were incubated with primary antibody at 4°C for 16 h, followed by 3 times washes. The sections were then incubated with a secondary antibody at 37°C for 30 min. DAB coloration was applied, and the nucleus was counterstained with hematoxylin. After dehydration, sections were sealed with neutral resin. The antibodies used in this study are listed in Supplementary Table S2. The Aβ-16 was visualized and imaged using a fluorescence microscope and quantified with ImageJ software.

### Immunofluorescence

The collected brain tissues and cell slices were dewaxed and fixed with 4% paraformaldehyde at −20°C for 10 min. Following fixation, the tissues and cells were permeabilized with 0.1% Triton X-100 (Solarbio, Beijing, China) at room temperature for 10 min. They were then blocked with 10 g/L bovine serum albumin (Solarbio, Beijing, China) for another 10 min and incubated overnight at 4°C with primary antibodies. After 3 washing, the secondary antibody was applied and incubated at room temperature for 4 h. The antibodies used in this study are listed in Supplementary Table S2. The cell nuclei were stained with 4′,6-diamidino-2-phenylindole (DAPI; Solarbio, Beijing, China) for 5 min at room temperature. Finally, the brain tissues and cells were observed and digitally captured using a fluorescence microscope.

### Transcriptome sequencing and analysis

Total RNA was extracted from the collected hippocampus tissues using Trizol Reagent, following the standard phenol/chloroform phase separation method. The RNA concentration and integrity were measured with a NanoDrop ND-2000 spectrophotometer and an Agilent Bioanalyzer 2100 (Agilent Technologies; Santa Clara, CA, United States). Only samples with an RNA integrity number (RIN) above 0.8 were selected for subsequent sequencing. Complementary DNA libraries were constructed using the Illumina TruSeq RNA Sample Prep Kit (Illumina; San Diego, CA, United States), resulting in an average fragment size of 150 bp, excluding adaptors. The quality and integrity of these libraries were assessed with an Agilent 2100 Bioanalyzer and an ABI StepOnePlus Real-Time PCR System (Thermo Fisher Scientific; Waltham, MA, United States). The RNA-Seq FASTQ files were aligned to the rat genome using the Hisat2 algorithm (Kim et al., 2019), referencing data from the Ensembl Rat Genome Database (https://ftp.ensembl.org/pub/release-112/fasta/rattus_norvegicus/). Cufflinks (Trapnell et al., 2012) were used to process the resulting binary alignment/map (BAM) files to evaluate transcript abundance and identify potential mRNA isoforms. Additionally, StringTie (v1.3.3b) (Pertea et al., 2015) was used to assemble the mapped reads from each sample using a reference-based approach. Transcript expression levels were quantified in terms of fragments per kilobase million (FPKM), and Principal Component Analysis (PCA) was conducted based on these FPKM values. The Kyoto Encyclopedia of Genes and Genomes (KEGG) and gene ontology (GO) enrichment analyses of differentially expressed genes (DEGs) were performed using DESeq2 software (Love et al., 2014) and the ClusterProfiler R package (Wu et al., 2021). The Benjamini-Hochberg method was applied to control the false discovery rate.

### Statistical analysis

Unless specified otherwise, all data were expressed as mean ± standard error and analyzed statistically using SPSS and RStudio software. Initially, continuous variables were tested for normal distribution and variance homogeneity. If the data satisfied both conditions, an independent samples analysis of variance (ANOVA) was employed for preliminary hypothesis testing, followed by the LSD method for *post hoc* analysis. For data not meeting these criteria, the Kruskal-Wallis test was used, with the Tukey method for *post hoc* testing. For repeated-measures data (e.g., escape latency), a repeated measures ANOVA method was applied to assess differences across groups and time points. A P value less than 0.05 was considered indicative of statistical significance in all hypothesis tests. In transcriptomics analysis, the similarity of gene expression profiles among different groups was evaluated using the permutational multivariate analysis of variance (PERMANOVA) method. In differential expression analysis, genes with |Log2 (FoldChange)| ≥ 1 and *p*-value < 0.05 were deemed significantly differentially expressed genes (DEGs). In KEGG and GO enrichment analysis signaling pathways with *p* values < 0.05 were considered significantly enriched. Data plots were created using OriginPro software (v2024), while all schematic diagrams and microscope images were illustrated or enhanced using Adobe Illustrator and Adobe Photoshop unless otherwise noted.

## Results

### Evaluation of formulations for optimal therapeutic effects

**FIGURE 1:**
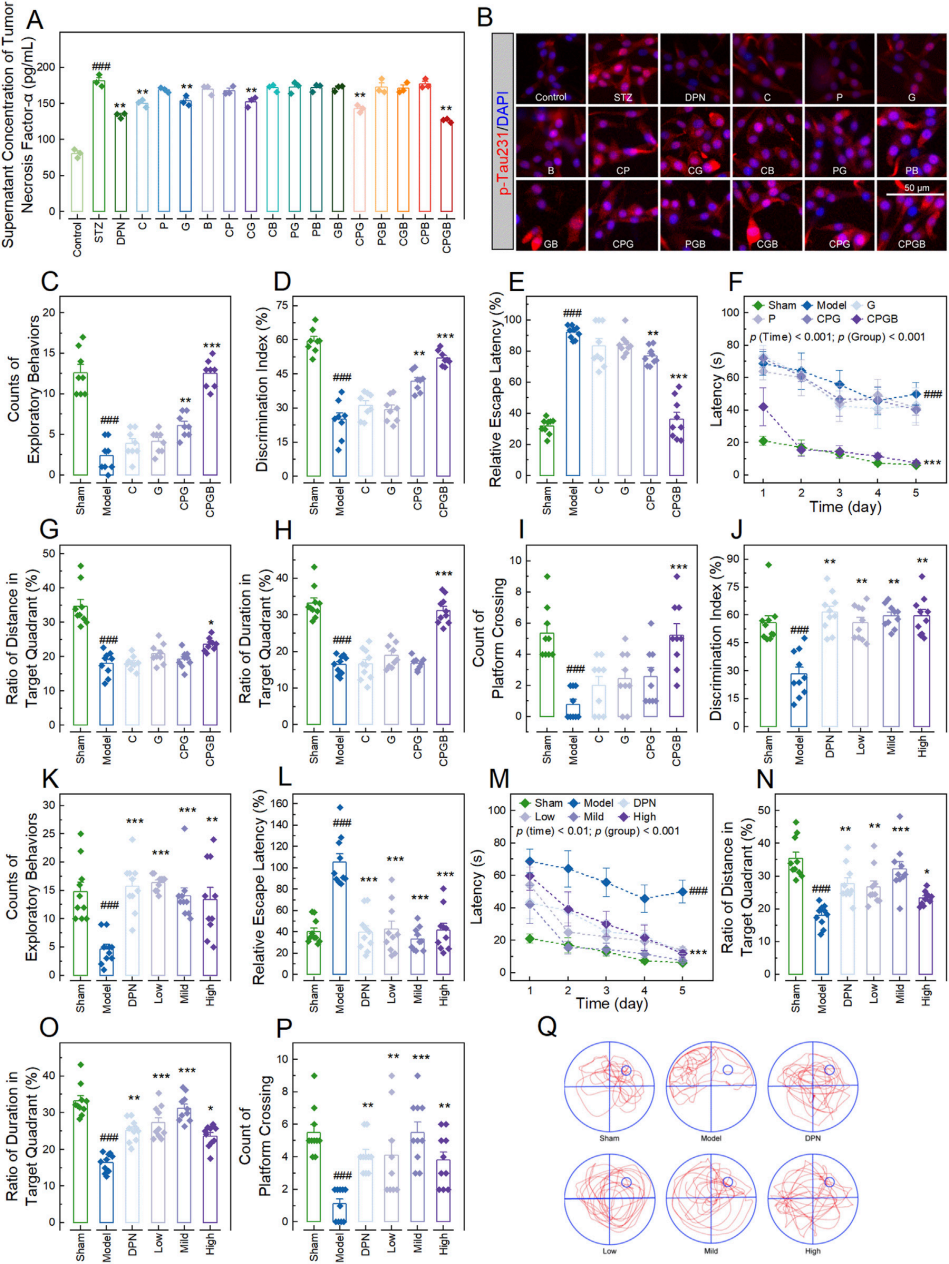
Evaluation of formulations for optimal therapeutic effects and their impact on cognitive behaviors in Alzheimer’s disease rats. **(A)**: Bar chart with dots showing the concentration of tumor necrosis factor-α in the supernatant of PC12 cells treated with various additives (n = 3); **(B)**: Representative microscopy images showing the p-Tau231in PC12 cells treated with various additives (n = 3); **(C, D, J, K)**: Bar charts with dots illustrating the counts of exploratory behaviors (**(C, K)**; n = 8) and discrimination indexes (**(D, J)**; n = 8–10) of rats in the new object recognition test; **(E, L)**: Bar charts with dots of the relative escape latency of rats (n = 10); **(F, M)**: Dot-line plots presenting the latencies of rats over the 5 days of Morris water maze tests (n = 10); **(G–I, N–P)**: Bar charts with dots representing the ratios of distance (**(G, N)**; n = 10) and duration (**(H, O)**; n = 10) of rats in the target quadrant, along with the platform crossing counts of rats (**(I, P)**; n = 10) in the Morris water maze tests; and **(Q)**: Swim paths illustrating the representative trajectories of rats in the Morris water maze test. In **(A, C–P)**, data are expressed as mean ± standard error. Statistical analyses were performed using one-way analysis of variance **(A, D, E, G, H, J, L, N–P)**, the Kruskal-Wallis test **(C, I, K)**, and repeated measures one-way analysis of variance **(F, M)**: ^###^, *p* < 0.001 compared to the Control/Sham group; *, *p* < 0.05, **, *p* < 0.01, ***, *p* < 0.001 compared to the STZ/Model group. STZ, streptozotocin; DPN, donepezil hydrochloride tablets; p-Tua231, phosphorylated Tau protein at threonine 231; DAPI, 6-diamidino-2-phenylindole.

To investigate the therapeutic effects of the 15 formulations on AD, we first measured concentrations of proinflammatory cytokines in the supernatant of PC12 cells treated with STZ and various formulations. The results indicated that STZ treatment significantly elevated the levels of TNF-α, IL-1β, IL-6, and Aβ1-42 (*p* < 0.001; Figure 1A; Supplementary Figures S2A–C) in the supernatant, as well as the expression of p-Tau231 (*p* < 0.001; Figure 1B; Supplementary Figure S2D) in PC12 cells. These increases were significantly mitigated by the positive drug DPN and 4 formulations: C, G, CPG, and CPGB (*p* < 0.01), while other treatments did not show significant effects (*p* > 0.05) (Figures 1A, B; Supplementary Figures S2A–D).

Next, we examined the therapeutic effects of these 4 formulations in an AD rat model. Rats in each group exhibited similar probe times and exploration speeds in the test phase of the NOR test and swimming speeds in the MWM test (*p* > 0.05; Supplementary Figures S2E–G). However, AD rats demonstrated significant reductions in exploratory behavior counts and discrimination index in the NOR test, as well as in the ratios of distance and duration of swimming in the target quadrant, and platform crossing counts in the MWM test (*p* < 0.001; Figures 1C, D, G–I). They also showed significant increases in relative escape latency in the EMP test and latency in the MWM test (*p* < 0.001; Figures 1E, F). These changes were significantly ameliorated by CPGB treatment (*p* < 0.05), but not by C or G treatments (*p* > 0.05; Figures 1C–I). Although CPG treatment exhibited some mitigating effects, its therapeutic impact was notably less than that of CPGB treatment (*p* < 0.05; Figures 1C–I). Hence, we chose CPGB as our formulation in the following experiments.

### CPGB mitigated STZ-induced AD-related cognitive behaviors in rats

After confirming that CPGB exhibits greater therapeutic effects on AD rats, we further explored the impacts of low (90 mg/kg), mild (180 mg/kg), and high (360 mg/kg) doses of CPGB on the cognitive behaviors of AD rats. In the familiarization phase of the NOR test and MWM test, rats across all groups displayed similar exploratory behavior counts, probe times, and swimming speeds (*p* > 0.05; Supplementary Figures S1H–J). However, during the test phase of the NOR test, DPN and all 3 doses of CPGB significantly improved the STZ-induced reductions in exploratory behavior counts and discrimination index, independent of dose (*p* < 0.01; Figures 1J, K). STZ treatment significantly increased the relative escape latency and latency in EMP and MWM tests (*p* < 0.001; Figures 1L, M). Both DPN and CPGB treatments significantly reduced these increases (*p* < 0.01), with the mild dose of CPGB resulting in significantly lower relative escape latency and latency compared to the low or high doses (*p* < 0.05; Figures 1L, M). In the MWM test, DPN and CPGB treatments also significantly mitigated the STZ-induced reductions in the ratios of distance and duration of swimming in the target quadrant, as well as in platform crossing counts (*p* < 0.05; Figures 1N–P). Rats treated with the mild dose of CPGB showed significantly higher ratios of distance and duration of swimming in the target quadrant and platform crossing counts compared to those treated with low or high doses (*p* < 0.05; Figures 1N–P). Detailed trajectories of rats in the MWM test are visualized in Figure 1Q.

### CPGB ameliorated STZ-induced AD-related pathological changes in rats

**FIGURE 2:**
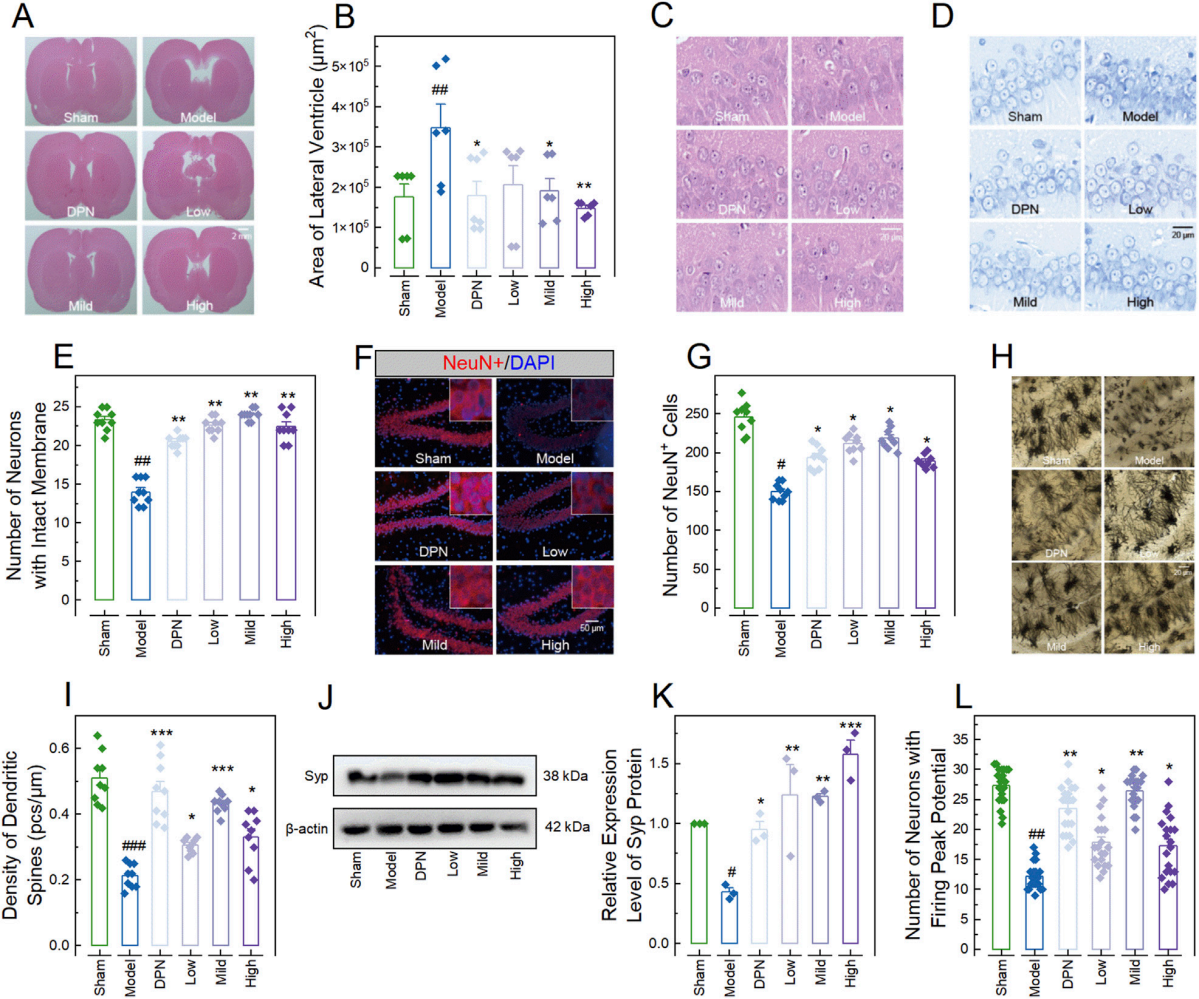
CPGB attenuated streptozotocin-induced pathological changes in rats. **(A, C, D, F, H)**: Representative microscopy images of hematoxylin and eosin-stained brain sections **(A)** and hippocampal tissue sections **(C)**, Nissl-stained bodies in hippocampal tissue sections **(D)**, NeuN staining in hippocampal tissue section **(F)**, and Golgi staining in hippocampal tissue sections **(H)**; **(B, E, G, I, K, L)**: Bar charts with dots representing the areas of lateral ventricles (n = 6–7; **(B)**), number of neurons with intact membranes (n = 9; **(E)**), number of NeuN^+^ cells (n = 9; **(G)**), density of dendritic spines (n = 9; **(I)**), relative gray values of Spy protein (n = 3; **(K)**), and number of neurons with firing peak potential (n = 21; **(L)**) in rats across different groups; and **(J)**: Representative gel images showing the expression level of Syp protein. In **(B, E, G, I, K, L)**, data are expressed as mean ± standard error. Statistical analyses were conducted using one-way analysis of variance **(B, I, K)** and the Kruskal-Wallis test **(E, G, L)**: ^#^, *p* < 0.05, ^##^, *p* < 0.01, ^###^, *p* < 0.001 compared to the Sham group; *, *p* < 0.05, **, *p* < 0.01, ***, *p* < 0.001 compared to the Model group. DPN, donepezil hydrochloride tablets; DAPI, 6-diamidino-2-phenylindole.

To further validate the therapeutic effects of CPGB on AD in rats, we evaluated the pathological changes in the lateral ventricle and hippocampus, as well as the electrophysiological dysfunction of hippocampal neurons. HE staining results showed that STZ treatment significantly enlarged the lateral ventricles in rats (*p* < 0.01). This enlargement was significantly reduced by treatments with mild and high doses of CPGB, as well as DPN (*p* < 0.05), but not by a low dose of CPGB (*p* > 0.05) (Figures 2A, B). Additionally, most hippocampal neurons in the Model group were degenerated, displaying blurred cell contours, ruptured cell bodies, and irregular arrangements compared to the Sham group. In contrast, neurons in rats treated with DPN or CPGB were intact and well-organized with clear outlines (Figure 2C). Nissl staining and immunofluorescence results indicated that the nuclear structure in the hippocampus of the Model group was unclear, with indistinct surrounding Nissl bodies. The number of neurons with intact membranes and NeuN^+^ cells in the hippocampus was significantly decreased in the Model group compared to the Sham group (*p* < 0.05). Rats treated with DPN and CPGB exhibited distinct Nissl bodies around the nucleus and a significantly increased number of neurons with intact membrane and NeuN^+^ cells compared to the Model group (*p* < 0.05) (Figures 2D–G). Golgi staining results demonstrated that the density, branch number, and total length of dendritic spines in hippocampal neurons were significantly lower in the Model group compared to the Sham group (*p* < 0.001). Treatments with DPN and CPGB significantly mitigated these decreases (*p* < 0.01), with a mild dose of CPGB exhibiting a better mitigative effect than the low and high doses (*p* < 0.05) (Figures 2H, I). Immunoblotting results showed that DPN and CPGB treatments significantly reversed the STZ-induced downregulation of Syp protein in hippocampal neurons (*p* < 0.05) (Figures 2J, K). Finally, the electrophysiological test identified a significantly decreased number of neurons with firing peak potentials in the hippocampal of the Model group (*p* < 0.01), which was significantly lower than in those treated with DPN and CPGB (*p* < 0.05) (Figure 2L).

### CPGB attenuated STZ-induced increases of AD-related markers in rats

**FIGURE 3:**
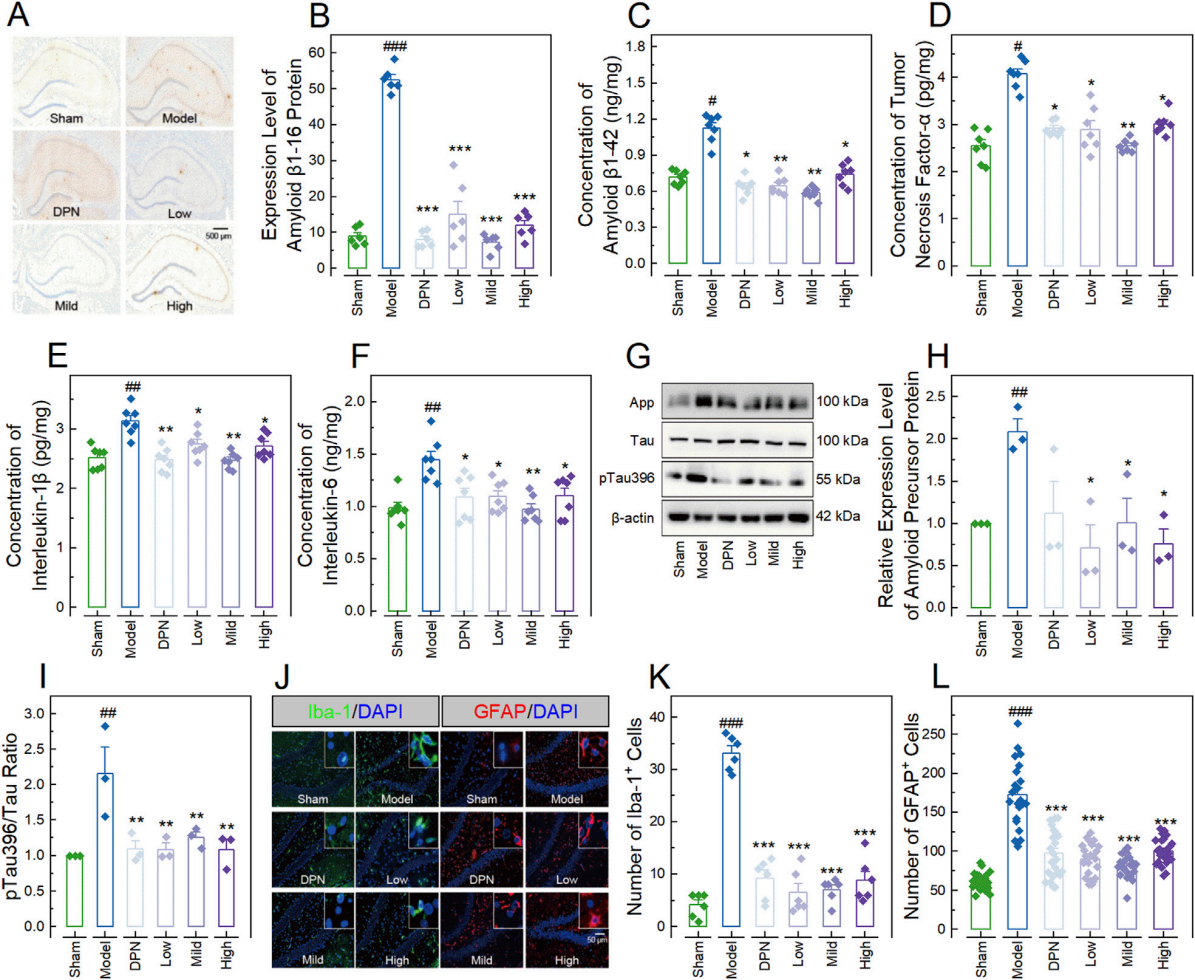
CPGB Mitigated increases in Alzheimer’s disease-related markers induced by streptozotocin in rats. **(A, J)**: Representative microscopy images showing stained amyloid β1-16 protein **(A)**, Iba-1, and GFAP **(J)** in hippocampal tissue sections; **(B–F, H, I, K, L)**: Bar charts with dots representing the expression level of amyloid β1-16 protein (n = 6; **(B)**), concentrations of amyloid β1-42 (n = 7; **(C)**), tumor necrosis factor-α (n = 7; **(D)**), interlukin-1β (n = 7; **(E)**), and interlukin-6 (n = 7; **(F)**), the relative expression level of App (n = 3; **(H)**), the ratio of pTau396 to Tau (n = 3; **(I)**), as well as the numbers of cells expressing Iba-1 (n = 6; **(K)**) and GFAP (n = 20; **(L)**); and **(G)** Representative gel images showing the expression levels of App, Tau, and pTau396, in the hippocampus of rats. In **(B–F, H, I, K, L)**, data are expressed as mean ± standard error. Statistical analyses were conducted using one-way analysis of variance **(B–F, H, I)** and the Kruskal-Wallis test **(K, L)**: ^#^, *p* < 0.05, ^##^, *p* < 0.01, ^###^, *p* < 0.001 compared to the Sham group; *, *p* < 0.05, **, *p* < 0.01, ***, *p* < 0.001 compared to the Model group. DPN, donepezil hydrochloride tablets; DAPI, 6-diamidino-2-phenylindole; App, amyloid precursor protein; Iba-1, ionized calcium-binding adaptor molecule 1; GFAP, glial fibrillary acidic protein.

In the meanwhile, we also investigated the effects of CPGB on AD-related markers in rats, including Aβ protein deposition, Tau protein hyperphosphorylation, hippocampal glial cell activation, and pro-inflammatory cytokine production. Immunohistochemistry results demonstrated a significantly higher level of Aβ1-16 in the Model group compared to the Sham group (*p* < 0.001). This increase was significantly inhibited by treatments with DPN and CPGB (*p* < 0.001), with the mild dose of CPGB resulting in a lower Aβ1-16 level compared to the low and high doses (*p* < 0.05) (Figures 3A, B). ELISA results indicated that treatments with DPN and CPGB significantly reversed the STZ-induced increases of Aβ1-42, TNF-α, IL-1β, and IL-6 concentrations in the Model group (*p* < 0.05; Figures 3C–F). Immunoblotting analysis revealed a significantly higher relative expression of amyloid precursor protein (App) and a higher pTau396 to Tau ratio (*p* < 0.01), both of which were significantly inhibited by CPGB treatments (*p* < 0.05) (Figures 3G–I). Finally, immunofluorescence analysis showed a significant increase in the number of microglia expressing ionized calcium-binding adaptor molecule 1 (Iba-1) and astrocytes expressing GFAP in the Model group compared to the Sham group (*p* < 0.001). These increases were also significantly ameliorated by treatments with DPN and CPGB (*p* < 0.001) (Figures 3J–L).

### Investigating the underlying mechanism behind the therapeutic effects of CPGB on AD in rats

**FIGURE 4:**
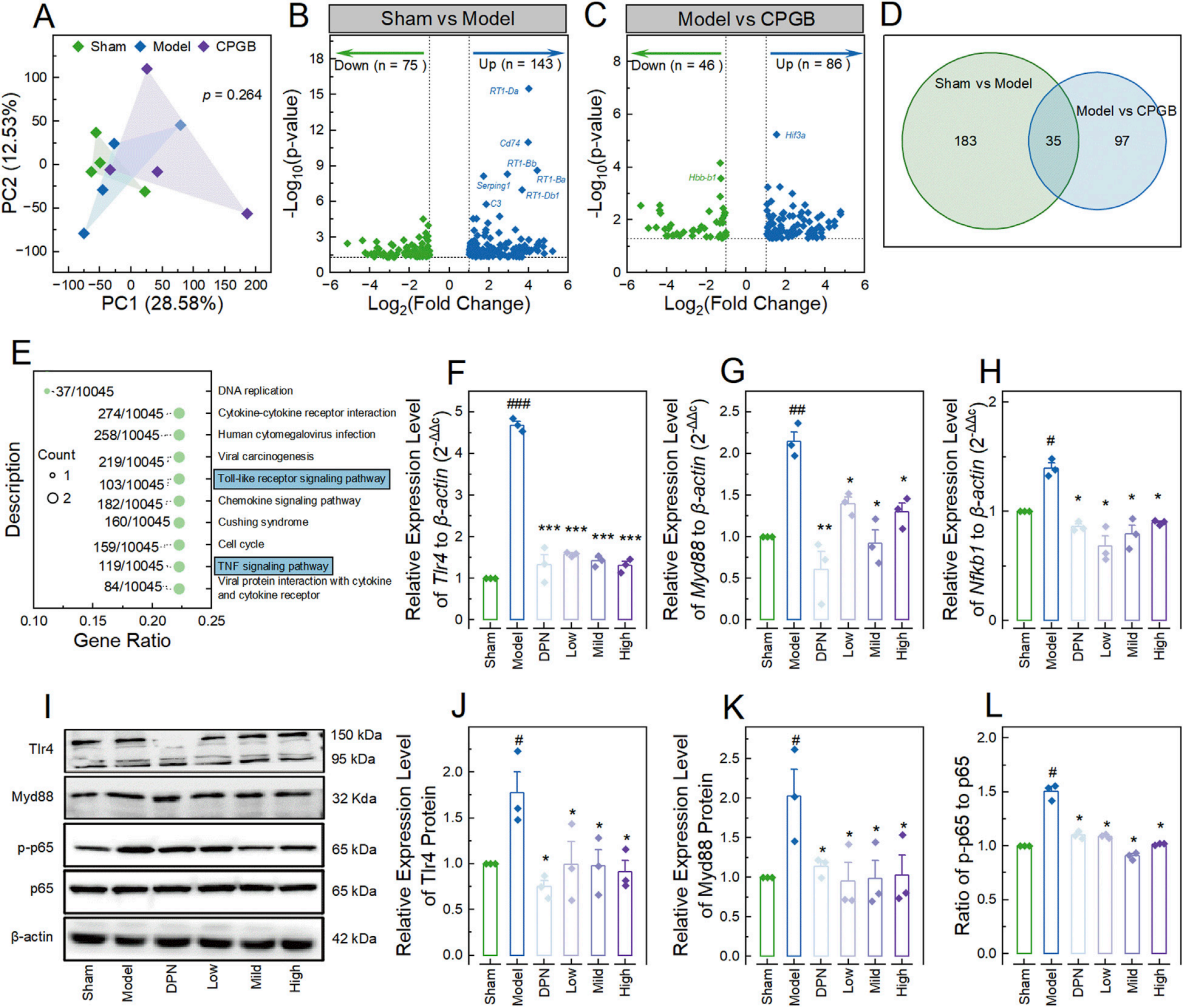
Investigating the underlying mechanisms behind the therapeutic effects of CPGB on Alzheimer’s disease in rats. **(A)**: Principal coordinate analysis plot based on unweighted unifrac distances, showing the variance in gene expression profiles among groups (n = 4); **(B, C)**: Volcano plots representing the differentially expressed genes (DEGs) between the Sham and Model groups **(B)** and between the Model and CPGB groups **(C)**; **(D)**: Venn diagram illustrating the shared and unique DEGs between the 2 comparisons; **(E)**: Bubble chart showing the significantly enriched KEGG pathways by the shared DEGs in **(D)**; **(F–H, J–L)**: Bar charts with dot showing the relative expression levels of mRNA *Tlr4* **(F)**, *Myd88* **(G)**, as well as *Nfkb1* **(H)**, proteins Tlr4 **(J)** and Myd88 **(K)**,and the ratio of p-p65 to p65 **(L)** (n = 3); and **(I)**: Representative gel images showing the expression levels of Tlr4, Myd88, p-p65, and p65, in the hippocampus of rats. In A, a permutational multivariate analysis of variance algorithm was used to calculate the similarity of gene expression profiles among groups. In **(E)**, the size of bubbles indicates the count of genes enriched into that KEGG pathway; “Gene Ratio” represents the ratio of the count of enriched genes to the total number of genes; the label alongside each bubble indicates the counts of genes in that KEGG pathway relative to total number of genes in the category. In **(F–H, J–L)**, data are expressed as mean ± standard error. Statistical analyses were conducted using one-way analysis of variance: ^#^, *p* < 0.05, ^##^, *p* < 0.01, ^###^, *p* < 0.001 compared to the Sham group; *, *p* < 0.05, **, *p* < 0.01, ***, *p* < 0.001 compared to the Model group. DPN, donepezil hydrochloride tablets.

To uncover the underlying mechanisms of CPGB’s therapeutic effects on AD rats, we randomly collected hippocampus samples from the 3 groups of rats: Sham (n = 4), Model (n = 4), and Mild (n = 4) groups. Then, we analyzed their mRNA expression profiles using transcriptome sequencing. This process generated a total of 141.93 Gbp of raw bases and 946.16 million raw reads, averaging 11.83 Gbp raw bases and 78.85 million raw reads per sample (Supplementary Table S3). After quality control and assembly, we annotated and quantified 20057 genes across the 12 hippocampal samples (Supplementary Table S4). PCA and PERMANOVA demonstrated no significant differences in gene expression profiles among groups (*p* = 0.264; Figure 4A). In the model group, compared to the Sham group, we identified 75 down-regulated and 143 up-regulated DEGs (Figure 4B; Supplementary Table S5). Similarly, in the CPGB group, compared to the Model group, there were 46 down-regulated and 86 up-regulated DEGs (Figure 4C; Supplementary Table S5). We further identified 35 DEGs common to both comparisons, including *Pkib*, *Hif3a*, *SNORD115*, *AABR07049688.1*, *Clec3a*, *LOC690082*, *AABR07036007.1*, *Ccr3*, *Ros1*, *Fndc9*, *AABR07053635.2*, *Wnt10b*, *Nod2*, *AABR07037487.1*, *U4*, *Tmem71*, *LOC100363125*, *Cdk1*, *Phex*, *PVR*, *AABR07047576.1*, *Ramp3*, *Cyp11b2*, *Spats1*, *Clec12b*, *AABR07043510.1*, *Aspg*, *AABR07019663.1*, *C7*, *Ccl5*, *Fam110d*, *Mcm3*, *AABR07028036.1*, *Vsig8*, and *Metazoa_SRP* (Figure 4D). These genes were enriched in 40 GO terms, primarily related to immune-inflammatory response regulation (Supplementary Table S6), and in 10 KEGG signaling pathways, including Viral protein interaction with cytokine and cytokine receptor, TNF signaling pathway, Cell cycle, Cushing syndrome, Chemokine signaling pathway, Toll-like receptor signaling pathway, Viral carcinogenesis, Human cytomegalovirus infection, Cytokine-cytokine receptor interaction, and DNA replication (Supplementary Table S6; Figure 4E).

Given the elevated pro-inflammatory responses in the hippocampus and the critical roles of the TLR and NF-κB pathways in neural inflammatory regulation (Squillace and Salvemini, 2022), we hypothesized that CPGB exerts its therapeutic effects on AD in rats by modulating the TLR4/Myd88/NF-κB signaling pathway. To test this hypothesis, we measured the relative expression levels of the *Tlr4*, *Myd88*, and *Nfkb1* genes compared to the housekeeping gene *β-actin* using the RT-qPCR method. The results showed significantly higher relative expression levels of *Tlr4*, *Myd88*, and *Nfkb1* in the Model group compared to the Sham group (*p* < 0.01). These increases were significantly reduced by treatments with DPN and CPGB (*p* < 0.05) (Figures 4F–H). Similarly, immunoblotting analysis also revealed significant increases in the relative express levels of Tlr4 and Myd88 proteins, as well as an increased ratio of p-p65 to p65 (*p* < 0.05). These increases were also significantly mitigated by DPN and CPGB treatments (*p* < 0.05) (Figures 4I–L).

### CPGB down-regulated the TLR4/Myd88/NF-κB signaling pathway and inhibited STZ-induced microglia neurotoxicity

**FIGURE 5:**
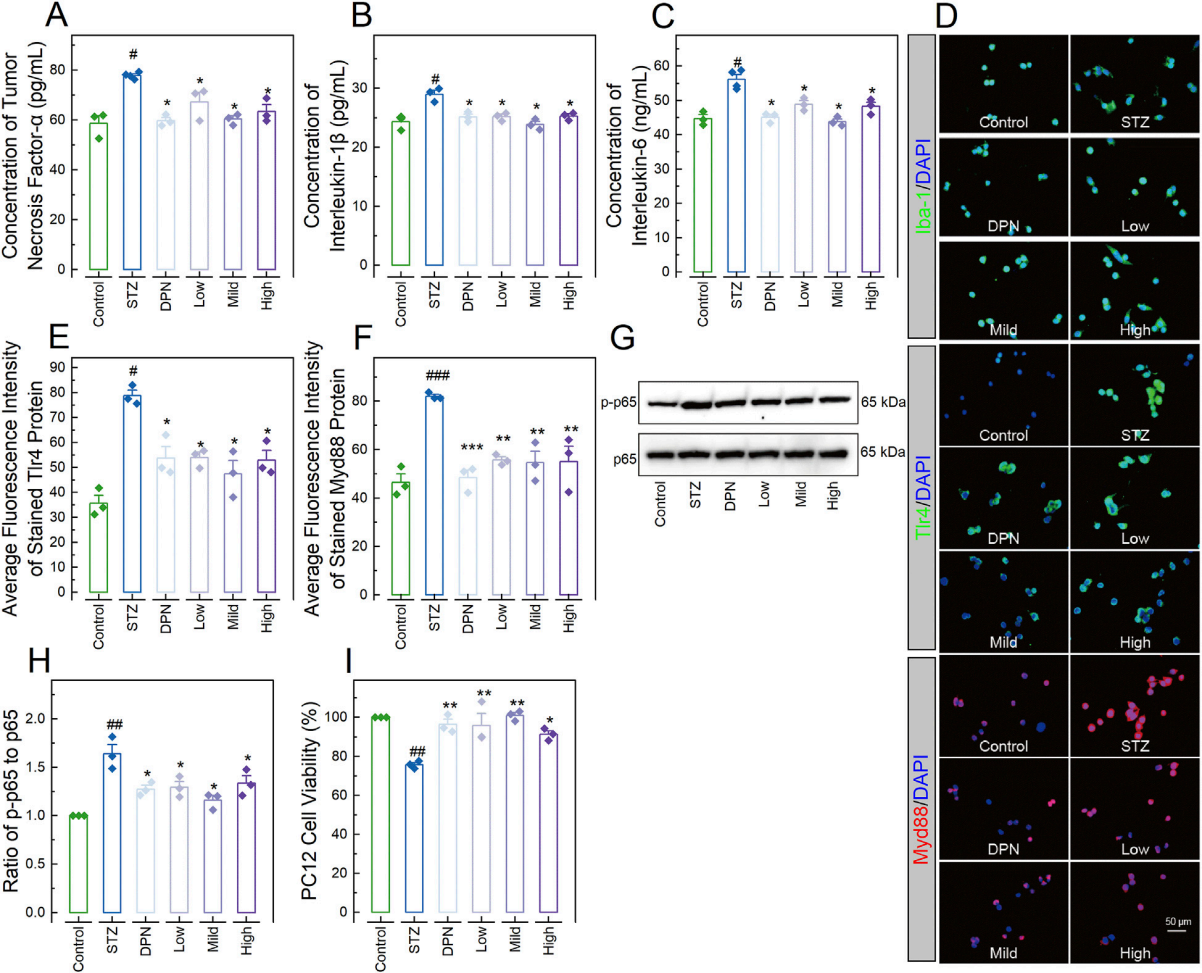
CPGB downregulated the TLR4/Myd88/NF-κB signaling pathway and inhibited STZ-induced microglia neurotoxicity. **(A–C, E, F)**: Bar charts with dots presenting the concentrations of pro-inflammatory cytokines tumor necrosis factor-α (n = 3; **(A)**), interleukin-1β (n = 3; **(B)**), and interleukin-6 (n = 3; **(C)**) in the supernatant of BV2 cells, as well as the average fluorescence intensity of stained Tlr4 (n = 3; **(E)**) and Myd88 (n = 3; **(F)**) proteins expressed in BV2 cells; **(D)**: Representative microscopy images showing the stained Iba-1, Tlr4, and Myd88 proteins expressed in BV2 cells; **(G)**: Representative gel images showing the expression levels of p-p65 and p65 proteins expressed in BV2 cells; **(H)**: Bar chart showing the relative gray values of pp65 and p65 proteins expressed in BV2 cells (n = 3); and **(I)**: Bar chart showing illustrating the viabilities of PC12 cells treated with conditioned medium from BV2 cells in each group (n = 3). In **(A–C, E, F, H, I)**: data are expressed as mean ± standard error. Statistical analyses were conducted using one-way analysis of variance: ^#^, *p* < 0.05, ^##^, *p* < 0.01, ^###^, *p* < 0.001 compared to the Control group; *, *p* < 0.05, **, *p* < 0.01, ***, *p* < 0.001 compared to the STZ group. STZ, streptozotocin; DPN, donepezil hydrochloride tablets; DAPI, 6-diamidino-2-phenylindole; Iba-1, ionized calcium-binding adaptor molecule 1.

To confirm the involvement of the TLR4/Myd88/NF-κB signaling pathway in the mitigating effects of CPGB on AD, we assessed pro-inflammatory responses and the activity of this signaling pathway in BV2 cells treated with STZ and 90 μg/mL (Low), 180 μg/mL (Mild), and 360 μg/mL (High) doses of CPGB. It was observed that the STZ challenge significantly increased the concentrations of pro-inflammatory cytokines TNF-α, IL-1β, and IL-6 in the supernatant of BV2 cells (*p* < 0.05; Figures 5A–C), as well as the expression levels Tlr4, Myd88 (*p* < 0.01; Figures 5D–F), and Iba-1 proteins, and the ratio of p-p65 to p65 (*p* < 0.01; Figures 5G, H) in BV cells. All these increases were significantly reduced by treatments with DPN and CPGB (*p* < 0.05; Figures 5A–H).

Additionally, PC12 cells treated with conditioned medium from BV2 cells in the STZ group demonstrated significantly lower viability compared to those treated with conditioned medium from BV2 in the Control group (*p* < 0.05; Figure 5I). In contrast, PC12 cells treated with conditioned medium from BV2 in the DPN and CPGB groups demonstrated significantly higher viability compared to those treated with conditioned medium from BV2 cells in the STZ group (*p* < 0.05; Figure 5I).

### CPGB attenuated STZ-induced pathogenesis of AD in a 3D-NVU model by inhibiting the TLR4/Myd88/NF-κB signaling pathway

**FIGURE 6:**
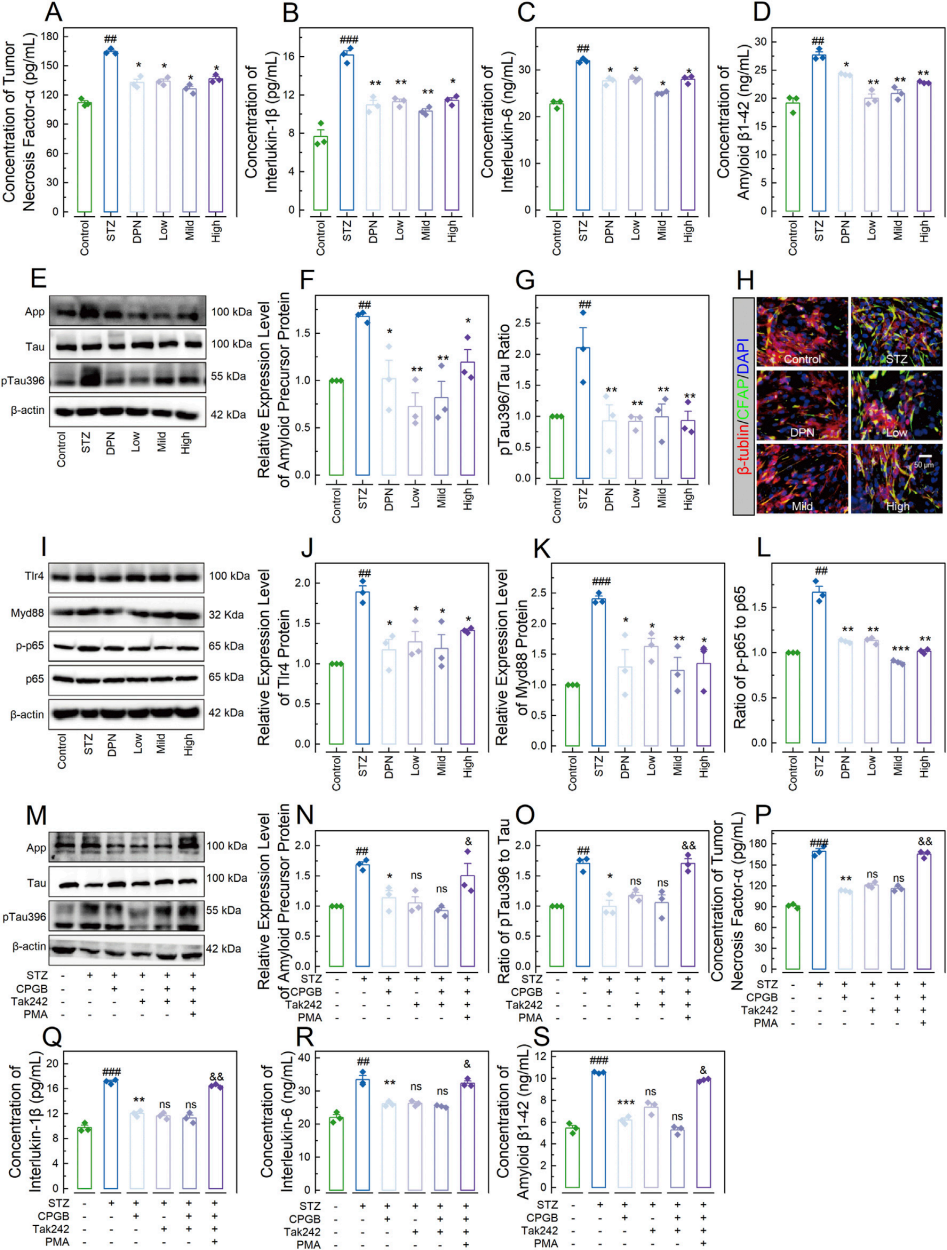
CPGB attenuated STZ-induced pathogenesis of Alzheimer’s disease in a 3-dimensional brain neurovascular unit model. **(A–D, P–S)**: Bar charts with dots representing the concentrations of tumor necrosis factor-α **(A, P)**, interleukin-1β **(B, Q)**, interleukin-6 **(C, R)**, and amyloid β1-42 protein **(D, S)** in the supernatant across different groups (n = 3); **(F, G, J–L, N, O)**: Bar charts showing the relative expression levels of App **(F, N)**, Tlr4 **(J)**, and Myd88 **(K)** proteins, as well as the ratio of pTau396 to Tau **(G, O)** and p-p65 to p65 **(L)** across different groups (n = 3); **(E, I, M)**: Representative gel images showing the expression levels of App, pTau396, and Tau proteins **(E, M)**, as well as Tlr4, Myd88, p-p65, and p65 proteins **(I)** across different groups; and **(H)**: Representative microscopy images showing the stained β-tubulin and CFAP across groups. In **(A–D, F, G, J–L, N–P)**, data are expressed as mean ± standard error. Statistical analyses were conducted using one-way analysis of variance: ^#^, *p* < 0.05, ^##^, *p* < 0.01, ^###^, *p* < 0.001 compared to the Control group; *, *p* < 0.05, **, *p* < 0.01, ***, *p* < 0.001 compared to the STZ group; ns: non-significant, ^&^, *p* < 0.05, ^&&^, *p* < 0.01. STZ, streptozotocin; DPN, donepezil hydrochloride tablets; DAPI, 6-diamidino-2-phenylindole; App, amyloid precursor protein; GFAP, glial fibrillary acidic protein; PMA, phorbol 12-myristate 13-acetate.

We also developed a 3D-NVU AD model, representing the core structure of AD pathological damage (Hongjin et al., 2020), and treated this model STZ and varying doses of CPGB: 90 μg/mL (Low), 180 μg/mL (Mild), and 360 μg/mL (High). We then assessed indicators related to AD and inflammatory response. As shown in Figures 6A–D, STZ treatment significantly increases the concentrations of TNF-α, IL-1β, IL-6, and Aβ1-42 in the supernatant (*p* < 0.01). These increases were significantly inhibited by treatments with DPN and CPGB (*p* < 0.05). Immunoblotting analysis confirmed that STZ treatment significantly elevated the relative expression level of APP and the ratio of p-Tau396 to Tau (*p* < 0.01). These elevations were also significantly reduced by treatments with DPN and CPGB (*p* < 0.05; Figures 6E–G). Additionally, immunofluorescence analysis showed that DPN and CPGB treatments reduced the expression level of GFAP in the model (Figure 6H). As expected, STZ treatment significantly increased the relative expression levels of Tlr4 and Myd88 proteins and the ratio of p-p65 to p65 (*p* < 0.01). These increases were significantly mitigated by treatments with DPN and CPGB (*p* < 0.05; Figures 6I–L).

To confirm the necessity of the TLR4/Myd88/NF-κB signaling pathway, we interfered with this pathway by inhibiting the Tlr4 protein using Tak242 (MilloporeSigma, Burlington, MA, United States) and activating the NF-κB complex with phorbol 12-myristate 13-acetate (PMA; MilloporeSigma, Burlington, MA, United States) in the 3D-NVU model treated with STZ and 180 mg/mL of CPGB. As shown in Figures 6M–O, Tak242 treatment did not enhance the inhibitory effects of CPGB on the STZ-induced increases in the relative expression level of App or the ratio of pTau396 to Tau (*p* > 0.05). In contrast, PMA treatment significantly blocked these increases (*p* < 0.05). Similarly, Tak242 treatment did not enhance the inhibitory effects of CPGB on the STZ-induced increases in the concentrations of TNF-α, IL-1β, IL-6, and Aβ1-42 (*p* > 0.05), whereas PMA treatment significantly blocked these increases (*p* < 0.05) (Figures 6P–S).

## Discussion

AD is one of the most expensive, lethal, and burdensome diseases of this century, affecting millions of people worldwide (Prince et al., 2015; Scheltens et al., 2021; Selkoe, 2021). In recent decades, multiple Chinese herbs and their extracts have been shown to attenuate the pathogenesis of AD (Han et al., 2020; Li et al., 2021). However, few studies have reported the therapeutic effects of a compound prescription in Chinese medicine. In this study, we investigated the therapeutic effects of CPGB, a formulation comprising 4 ingredients extracted from 4 different Chinese herbs, on AD employing cellular, organoid, and animal models.

Several models are available for studying AD, each with distinct advantages and limitations. Transgenic AD models, while accurately mimicking key features like Aβ deposition and Tau hyperphosphorylation, have significant drawbacks, including a long latency for the appearance of pathology (6–9 months) and complex, time-consuming breeding processes. Additionally, the limited number of animals with consistent pathological and cognitive symptoms further complicates their use in large-scale studies. Aβ or Tau protein injection models, which involve direct brain injections, often result in localized damage due to poor diffusion of the proteins, leading to inconsistent cognitive deficits. These models primarily simulate specific aspects of AD pathology but fail to represent the broader, multifactorial nature of the disease. The aluminum-induced model is another simple and cost-effective approach, but it only reproduces single pathological features, typically focusing on cognitive deficits rather than the full spectrum of AD-related pathology. Aging models, though relevant to AD pathogenesis, require long experimental periods and are often too expensive for routine use. Moreover, neuroinflammation models, such as those induced by lipopolysaccharide (LPS), can mimic inflammation-related cognitive deficits but lack the hallmark pathologies of AD, including Aβ accumulation. In contrast, the STZ-induced AD model offers several advantages. It reproduces a wide range of AD-like features, including ventricular enlargement, neuroinflammation, Aβ deposition, Tau hyperphosphorylation, oxidative stress, and synaptic dysfunction. Additionally, the STZ model is economically feasible and allows for rapid induction of AD-like pathology, making it well-suited for studies requiring timely results. Given its ability to mimic the complex, multifactorial nature of AD, particularly the sporadic form, it provides a robust model for investigating therapeutic interventions like CPGB (Drummond and Wisniewski, 2017; Yokoyama et al., 2022). Hence, we chose this model to explore the therapeutic effects of CPGB on AD.

Firstly, we selected 4 active ingredients, namely catalpol, puerarin, gastrodin, and borneol (Supplementary Figure S1), and developed 15 formulations through permutations and combinations. To identify a formulation with optimal therapeutic effects on AD, we established an AD cell model according to previous studies (Gupta et al., 2018; Kamat, 2015; Li et al., 2020), and evaluated the mitigative effects of these 15 formulations. The results showed that 4 formulations, including catalpol, gastrodin, CPG, and CPGB, significantly reduced concentrations of pro-inflammatory cytokines TNF-α, IL-1β, and IL-6 in the supernatant, as well as Aβ1-42 and the pTua231/Tau ratio (Figures 1A, B; Supplementary Figures S2A–D), which are considered AD-related indicators in cellular models (Lista et al., 2014). Subsequently, referencing our previous studies, we further evaluated the therapeutic effects of these formulations on AD using an STZ-induced AD rat model. The results demonstrated that CPGB produced greater improvements in STZ-induced Aβ protein deposition, pTua231 plaque formation, and cognitive deficits in the NOR and MWM tests, which are commonly used in AD rat models (Leger et al., 2013; Vorhees and Williams, 2006) (Figures 1C–I, 2). All these findings confirmed that CPGB was more effective in treating STZ-induced AD than other formulations, especially these monotherapy, suggesting that the great potential of CPGB in treating AD compared (Moreira-Silva et al., 2018).

Afterwards, we examined the therapeutic effects of low, mild, and high doses of CPGB, which were confirmed to show no significant toxicities on rats in our previous studies, on the behavioral, pathological, and molecular alterations induced by STZ. As anticipated, all doses of CPGB significantly alleviated STZ-induced cognitive deficits, AD-related pathological progressions, and molecular depositions in the hippocampus of rats (Figures 2, 3). The hippocampus, a core structure affected by AD, is composed of blood vessels (including endothelial cells, pericytes, and basement membrane), neurons, a variety of glial cells, and the extracellular matrix. Hence, to further explore CPGB’s effects, we established a 3D-NVU model, which reflects the dynamic integrity of brain structure and functions (Bhalerao et al., 2020; Caffrey et al., 2021), and validated the therapeutic effects of CPGB on AD at the organoid level. Similarly, CPGB significantly mitigated STZ-induced AD-related pathological progression and molecular deposition in this model (Figure 5). Together, these findings further confirm the efficacy of CPGB in preventing the pathogenesis of AD. Notably, we observed no significant differences in the AD-preventing efficacy of CPGB across different doses (Figures 2, 3, 5), suggesting a dose-independent therapeutic effect. However, this observation requires validation over a wider range of concentrations.

After confirming the effects, we further explored the underlying mechanisms. Through transcriptome sequencing and analysis, we identified 35 genes affected by both STZ and CPGB in the hippocampal tissues (Figures 4A–D). These genes were significantly enriched into 10 KEGG pathways (Figure 4E). Notably, the Toll-like receptor signaling pathway and NF-κB signaling pathway have been previously proven to play crucial roles in the pathogenesis of AD by regulating microglia-derived proinflammatory responses (Jha et al., 2019; Su et al., 2016). Therefore, we examined the activity of the Tlr4/Myd88/NF-κB signaling pathway by assessing the expression levels of its checkpoints in cellular and organoid models. As anticipated, CPGB significantly ameliorated STZ-induced increases in the relative expression levels of Tlr4, Myd88, and p65 proteins (Figures 4F–L, 5, 6A–L). Furthermore, inhibiting the Tlr4 protein did not enhance the therapeutic effects of CPGB, whereas activating the Myd88 protein significantly blocked these effects (Figures 6M–S). These findings suggest that the Tlr4/Myd88/NF-κB signaling pathway is essential for CPGB to exert its therapeutic effects on AD. It should be noted that we identified approximately 200 DEGs in each comparison, whereas a previous transcriptomic study identified thousands of DEGs (Mathys et al., 2019). This discrepancy suggests that other cells in the tissue, unaffected by STZ, might have influenced our transcriptome analysis results, explaining the lack of significant difference in gene expression profiles across groups (Figure 4A). Consequently, conducting a single-cell sequencing on rat hippocampal tissue or transcriptome sequencing on PC12 cells may reveal more DEGs and enrich additional signaling pathways, thus providing a broader understanding of potential mechanisms behind CPGB’s therapeutic effects on AD.

Our study is the first to combine 4 known bioactive compounds from TCMs with therapeutic potential for AD, demonstrating that this combination therapy is more effective than single-agent treatments. We have also preliminarily identified the molecular mechanisms by which this formulation affects microglial cells. Future experiments could further explore the pharmacological properties of this combination to provide theoretical support for its clinical application. However, our research has certain limitations. Notably, we did not investigate the compatibility of these 4 compounds or whether they interact with each other in ways that could impact the polypharmacological effect of this formulation, potentially influencing its therapeutic outcomes. This aspect warrants further investigation in future studies.

## Conclusion

In summary, our study demonstrated that the CPGB formula has superior mitigating effects on STZ-induced production of pro-inflammatory cytokines, as well as the deposition of Aβ proteins and hyperphosphorylated Tau proteins in PC12 cells. It also showed improvements in cognitive deficits in an AD rat model, compared to its components or other formulas. We further confirmed that varying doses of CPGB effectively prevented STZ-induced pathogenesis of AD, including cognitive deficits in rats, pathological changes in hippocampal tissues, and the production and deposition of AD-related markers in hippocampal tissues, 3D-NVU model, and BV2 cells. Additionally, our findings revealed that the Tlr4/Myd88/NF-κB signaling pathway is essential for the therapeutic effects of CPGB on AD pathogenesis. Our study enhances the theoretical foundation for using traditional Chinese medicine to treat AD, providing new insights and references for alleviating and treating AD.

## Data Availability

The raw data generated in the transcriptome sequencing have been uploaded to the NCBI (BioProject) database, accession number PRJNA1149930. All data and materials can be accessed from the corresponding author once requested.

## Supplementary material

SUPPLEMENTARY TABLE S1 Sequences of primers used in transcription-quantitative polymerase chain reaction analysis.

SUPPLEMENTARY TABLE S2 Detailed information about antibodies used in immunofluorescence, immunoblotting, and immunohistochemical experiments.

SUPPLEMENTARY TABLE S3 Detailed information about the generated raw data in transcriptome sequencing. Note: Q20 indicates the percentage of bases with a Phred quality score >20, signifying the reliability of base identification in the sequencing data.

SUPPLEMENTARY TABLE S4 Detailed information about the annotated and quantified genes for the generated data.

SUPPLEMENTARY TABLE S5 Detailed statistical information about the genes with *p*-value < 0.05.

SUPPLEMENTARY TABLE S6 Detailed results of gene ontology and kyoto encyclopedia of genes and genomes (KEGG) enrichment analyses.

SUPPLEMENTARY FIGURE S1 Chemical structure diagrams of the 4 plant-extracted compounds we used in this study.

SUPPLEMENTARY FIGURE S2 Evaluation of formulations for optimal therapeutic effects and their impact on cognitive behaviors in Alzheimer’s disease rats. **(A–C)**: Bar charts with dots depicting the concentrations of interleukin-1β, interleukin-6, and amyloid β1-42 in the supernatant of PC12 cell treated with various additives (n = 3); **(D)**: Bar chart with dot showing the average fluorescence intensity of the stained p-Tua231 protein in the PC12 cells treated with various additives (n = 3); **(E, F, H, I)**: Bar charts with dots illustrating the probe time **(E)**, exploration speed **(F, I)**, and exploratory behavior counts **(H)** of rats (n = 8–10) in the new object recognition tests; and **(G, J)**: Bar charts with dot representing the swimming speed of rats in the Morris water maze tests (n = 8–10). Data are expressed as mean ± standard error. Statistical analyses were performed using one-way analysis of variance **(A–G, I, J)** and the Kruskal-Wallis test **(H)**: ^###^, *p* < 0.001 compared to the Control/Sham group; *, *p* < 0.05, **, *p* < 0.01 compared to the STZ/Model group. STZ, streptozotocin; DPN, donepezil hydrochloride tablets; p-Tua231, phosphorylated Tau protein at threonine 231.

## Glossary

AD: Alzheimer’s Disease
3D-NVU: 3-dimensional brain neurovascular unit
AEA: arachidonoylethanolamide
ANOVA: analysis of variance
App: amyloid precursor protein
Aβ: amyloid beta
BMSCs: brain microvascular endothelial cells
DAPI: 4′,6-diamidino-2-phenylindole
DI: Discrimination index
DMEM/F12: Dulbecco’s modified eagle medium: Nutrient mixture F-12
DPN: donepezil hydrochloride tablets
ELISA: enzyme-linked immunosorbent assay
EMP: elevated plus maze
FBS: fetal bovine serum
FPKM: fragments per kilobase million
GFAP: glial fibrillary acidic protein
GO: gene ontology
HE: Haematoxylin and Eosin
ICV: intracerebroventricular
IL: interleukin
Iba-1: adaptor molecule 1
KEGG: Kyoto Encyclopedia of Genes and Genomes
MWM: Morris water maze
NO: nitric oxide
NOR: new object recognition
NSCs: neural stem cells
PC12: pheochromocytoma cell line
PCA: principal component analysis
PERMANOVA: permutational multivariate analysis of variance
PS: penicillin-streptomycin
ROS: reactive oxygen species
RT-qPCR: reverse transcription quantitative polymerase chain reaction
STZ: streptozotocin
TCMs: traditional Chinese medicine
TLR: toll-like receptor
TNF-α: tumor necrosis factor-α
cDNA: complementary DNA
rhEGF: recombinant human epidermal growth factor
rhbFGF: recombinant human basic fibroblast growth factor
