# Supplementary material for: Combined administration of catalpol, puerarin, gastrodin, and borneol modulates the Tlr4/Myd88/NF-κB signaling pathway and alleviates microglia inflammation in Alzheimer’s disease
Source: Front Pharmacol. 2024 Oct 31;15:1492237. doi: 10.3389/fphar.2024.1492237 (PMC11560463; doi:10.3389/fphar.2024.1492237)
Supplement: Supplementary file 1 [file Table1.DOCX]

| **Gene Name** | **Forward Primer Sequence** | **Reverse Primer Sequence** |
| --- | --- | --- |
| *Tlr4* | CCAGAGCCGTTGGTGTATCTT | GAGCATTGTCCTCCCACTCG |
| *Myd88* | CTCGCAGTTTGTTGGATGCC | CCAGTTCCTTTGTCTGTGGGA |
| *Nfkb1* | CTGAGTCTTCTGGACCGCTG | GCTGCCTTGCTGTTCTTGAG |
| *β-actin* | TGGCACCCAGCACAATGAA | CTAAGTCATAGTCCGCCTAGAAGCA |
