# Supplementary figures and images for: Combined administration of catalpol, puerarin, gastrodin, and borneol modulates the Tlr4/Myd88/NF-κB signaling pathway and alleviates microglia inflammation in Alzheimer’s disease
Source: Front Pharmacol. 2024 Oct 31;15:1492237. doi: 10.3389/fphar.2024.1492237 (PMC11560463; doi:10.3389/fphar.2024.1492237)

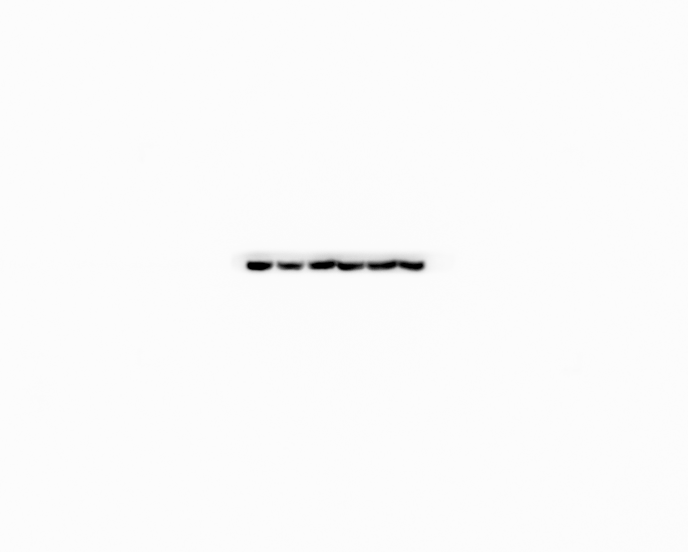

Supplement: Supplementary file 2 [file DataSheet1.ZIP › Original Gel Pictures/Figure 3 G/╬▓-actin-replicate 3.tif]

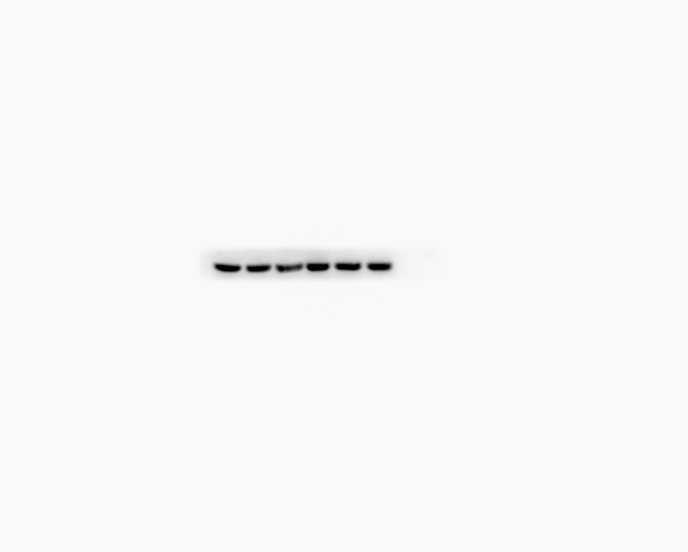

Supplement: Supplementary file 2 [file DataSheet1.ZIP › Original Gel Pictures/Figure 3 G/╬▓-actin-replicate 2.tif]

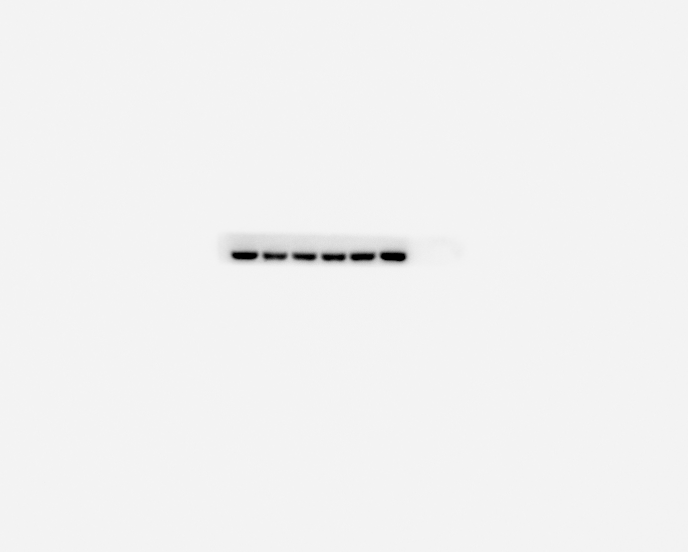

Supplement: Supplementary file 2 [file DataSheet1.ZIP › Original Gel Pictures/Figure 3 G/╬▓-actin-replicate 1.tif]

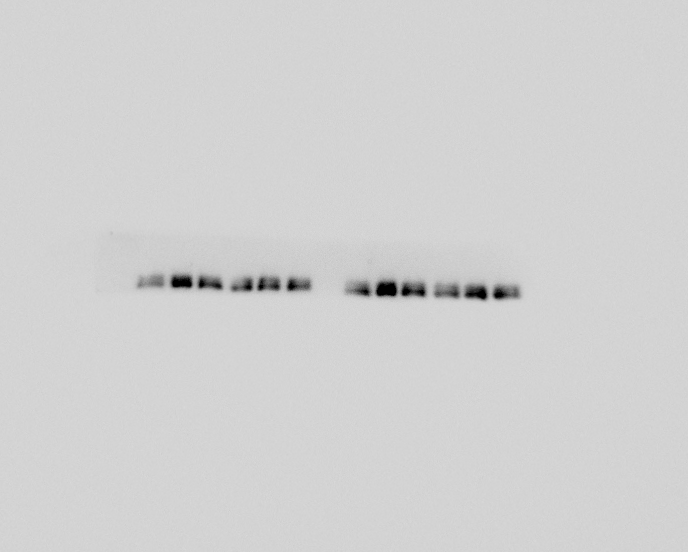

Supplement: Supplementary file 2 [file DataSheet1.ZIP › Original Gel Pictures/Figure 3 G/App-replicate 2.tif]

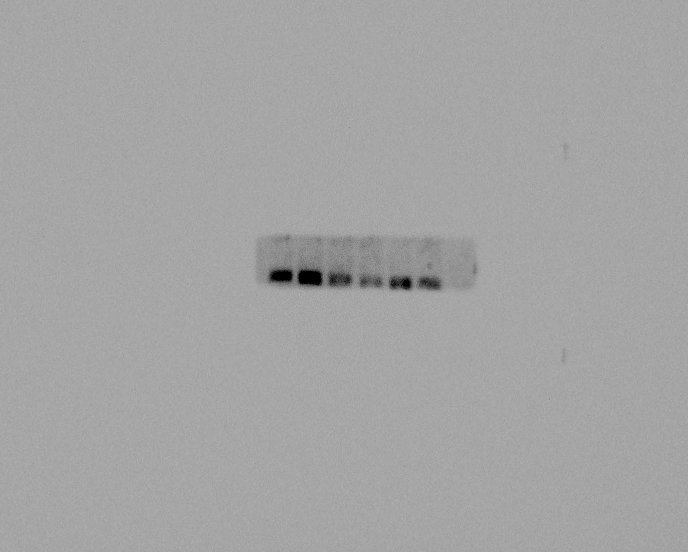

Supplement: Supplementary file 2 [file DataSheet1.ZIP › Original Gel Pictures/Figure 3 G/App-replicate 3.tif]

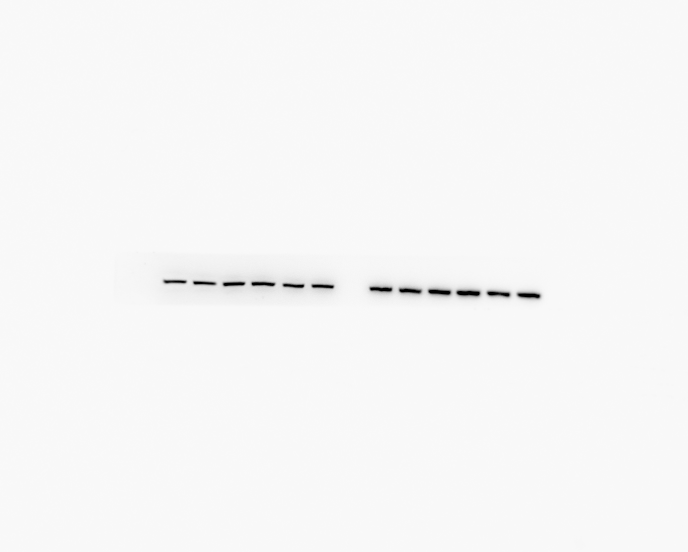

Supplement: Supplementary file 2 [file DataSheet1.ZIP › Original Gel Pictures/Figure 3 G/Tau-replicate 1.tif]

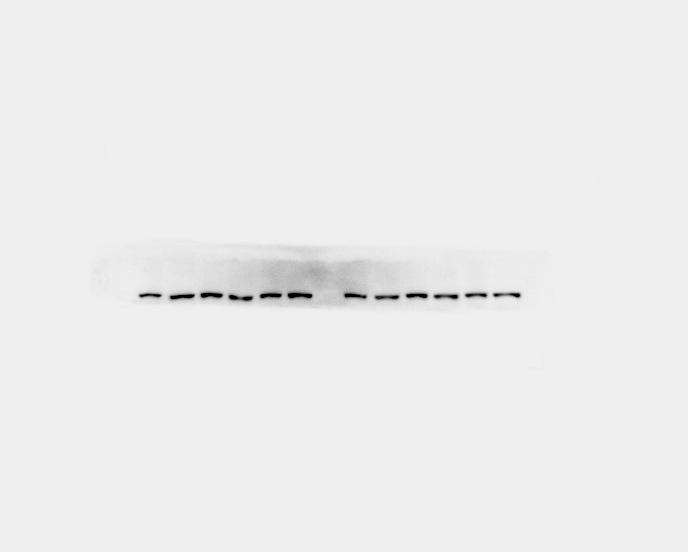

Supplement: Supplementary file 2 [file DataSheet1.ZIP › Original Gel Pictures/Figure 3 G/Tau-replicate 3.tif]

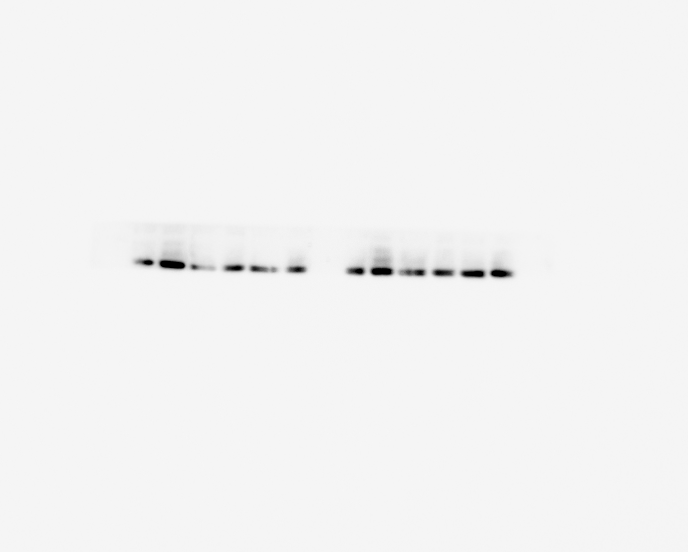

Supplement: Supplementary file 2 [file DataSheet1.ZIP › Original Gel Pictures/Figure 3 G/p-Tau396-replicate 1.tif]

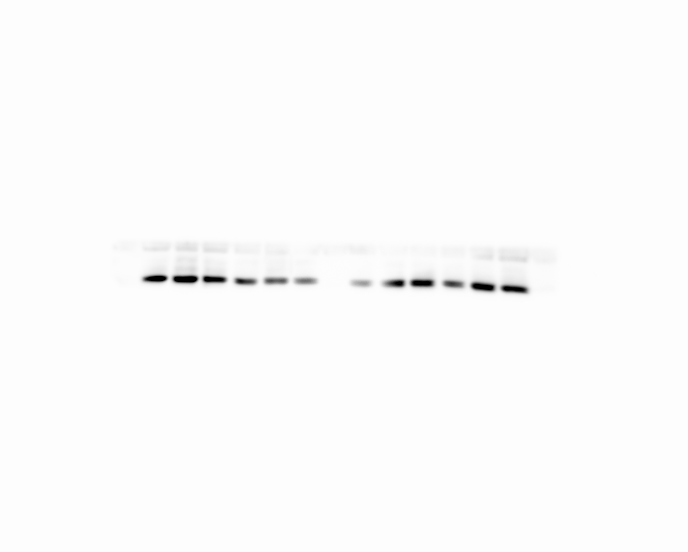

Supplement: Supplementary file 2 [file DataSheet1.ZIP › Original Gel Pictures/Figure 3 G/p-Tau396-replicate 3.tif]

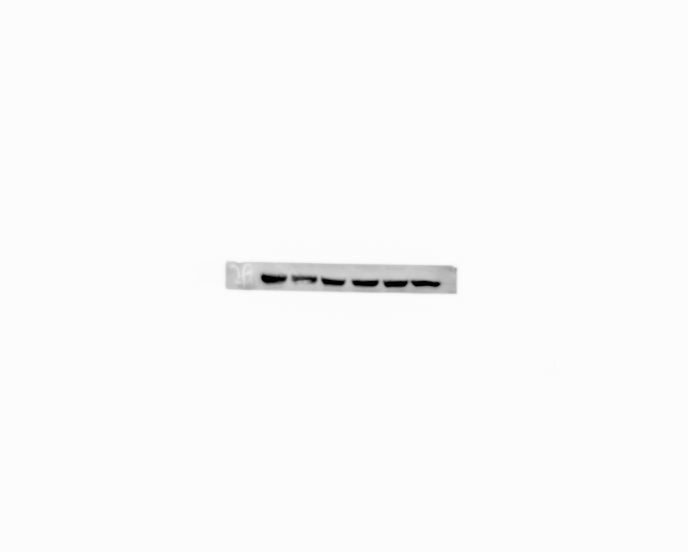

Supplement: Supplementary file 2 [file DataSheet1.ZIP › Original Gel Pictures/Figure 6 E/╬▓-actin-replicate 3.tif]

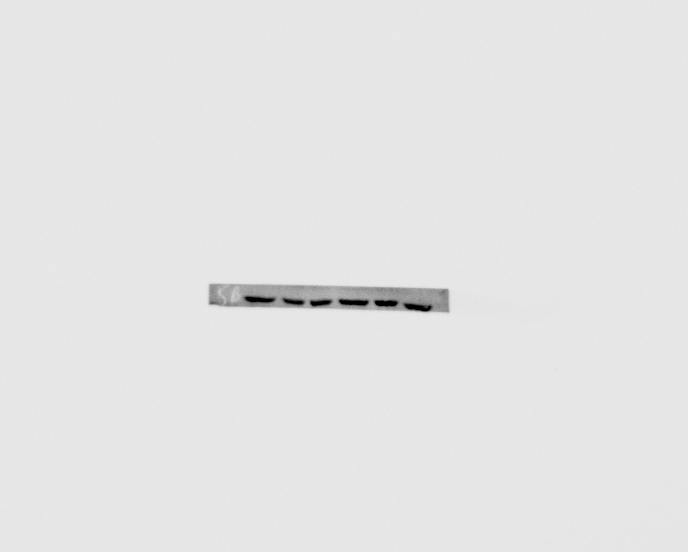

Supplement: Supplementary file 2 [file DataSheet1.ZIP › Original Gel Pictures/Figure 6 E/╬▓-actin-replicate 2.tif]

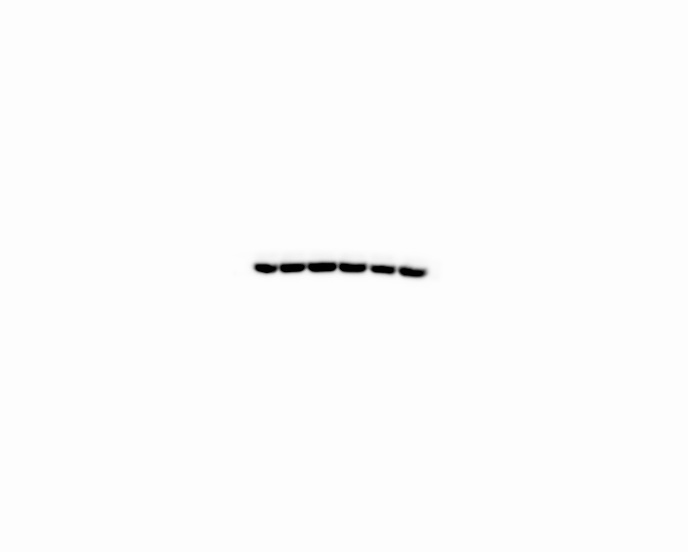

Supplement: Supplementary file 2 [file DataSheet1.ZIP › Original Gel Pictures/Figure 6 E/╬▓-actin-replicate 1.tif]

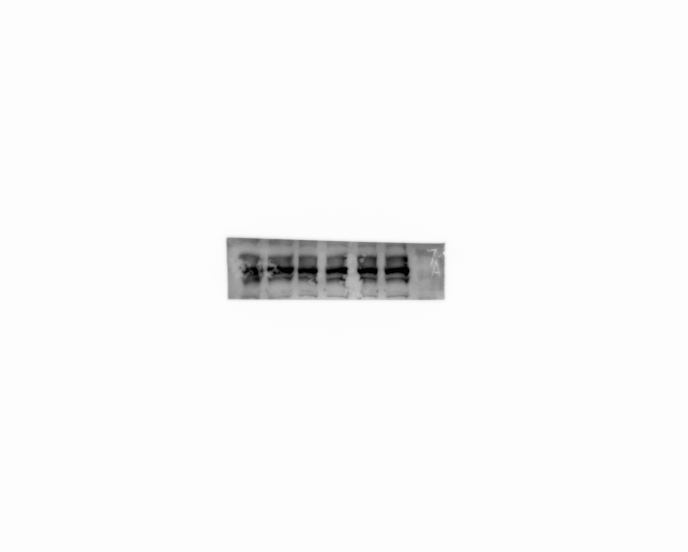

Supplement: Supplementary file 2 [file DataSheet1.ZIP › Original Gel Pictures/Figure 6 E/App-replicate 2.tif]

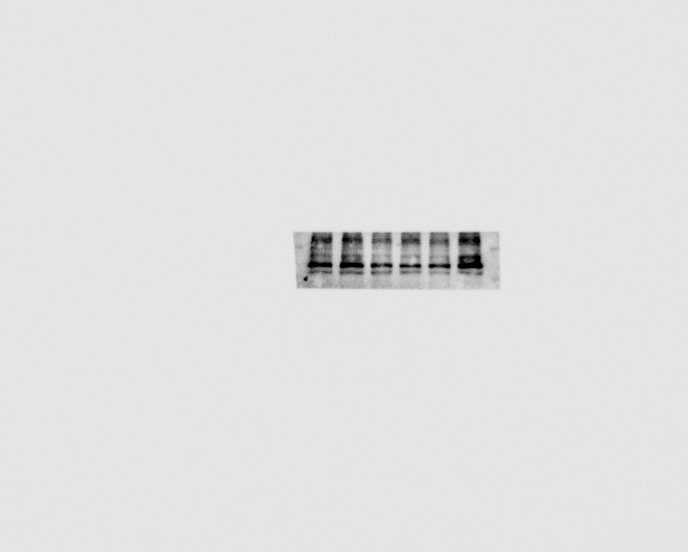

Supplement: Supplementary file 2 [file DataSheet1.ZIP › Original Gel Pictures/Figure 6 E/App-replicate 3.tif]

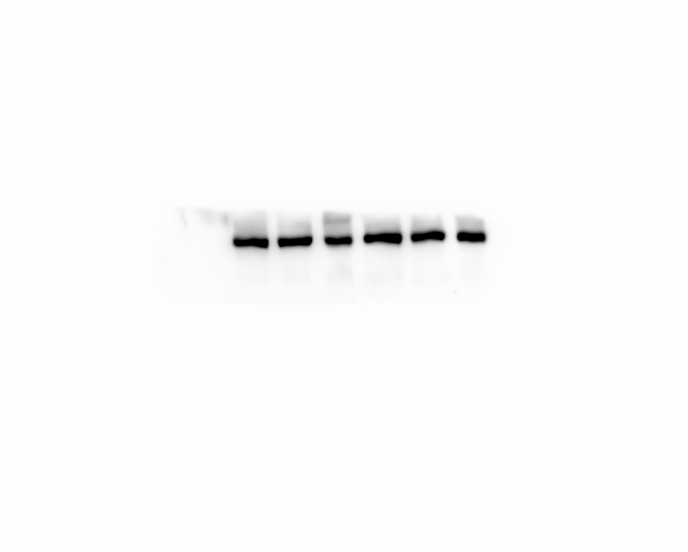

Supplement: Supplementary file 2 [file DataSheet1.ZIP › Original Gel Pictures/Figure 6 E/Tau-replicate 1.tif]

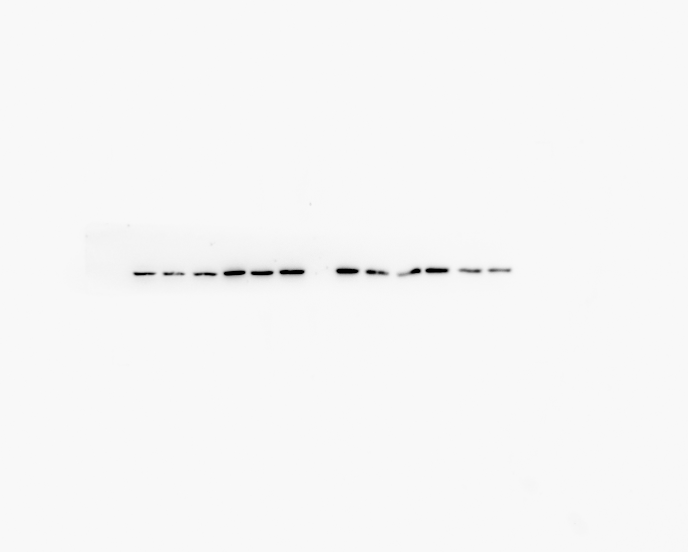

Supplement: Supplementary file 2 [file DataSheet1.ZIP › Original Gel Pictures/Figure 6 E/Tau-replicate 3.tif]

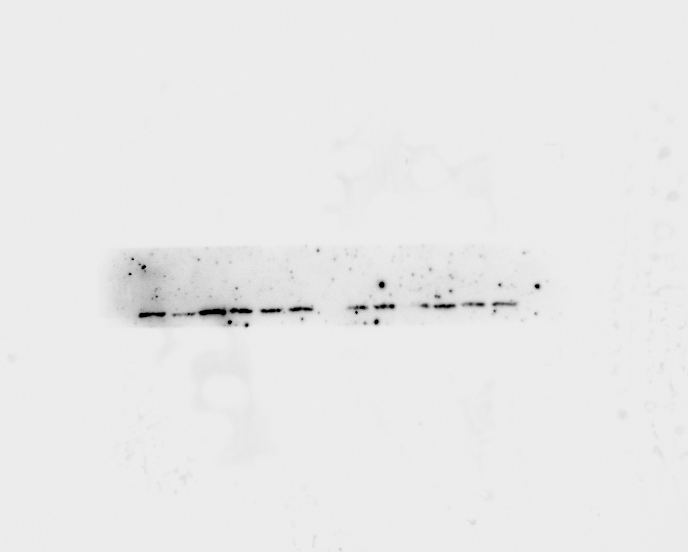

Supplement: Supplementary file 2 [file DataSheet1.ZIP › Original Gel Pictures/Figure 6 E/Tau-replicate 2.tif]

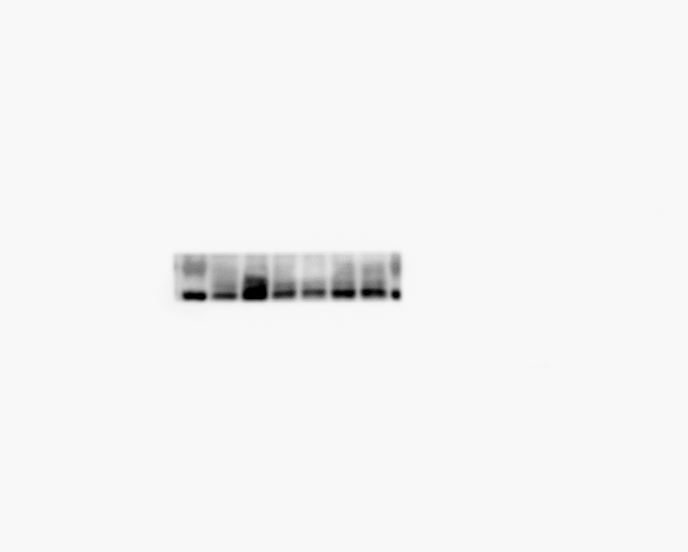

Supplement: Supplementary file 2 [file DataSheet1.ZIP › Original Gel Pictures/Figure 6 E/p-Tau396-replicate 1.tif]

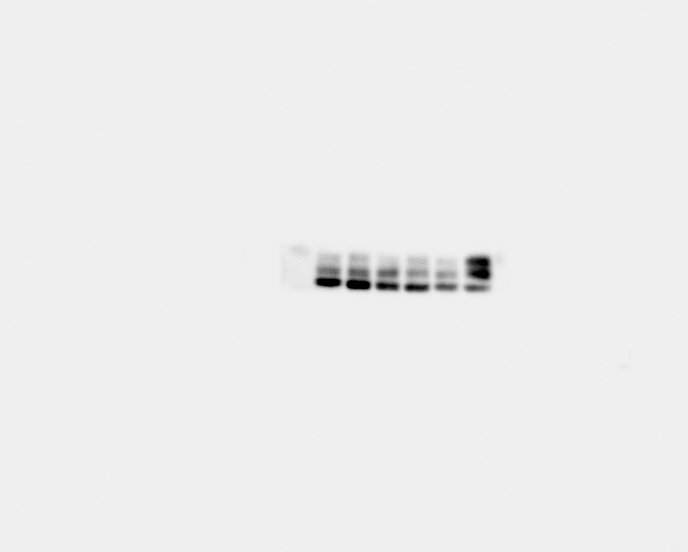

Supplement: Supplementary file 2 [file DataSheet1.ZIP › Original Gel Pictures/Figure 6 E/p-Tau396-replicate 2.tif]

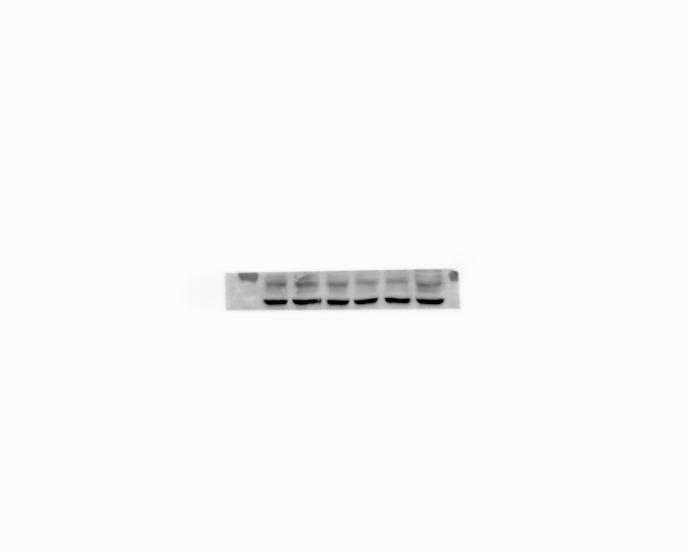

Supplement: Supplementary file 2 [file DataSheet1.ZIP › Original Gel Pictures/Figure 6 E/p-Tau396-replicate 3.tif]

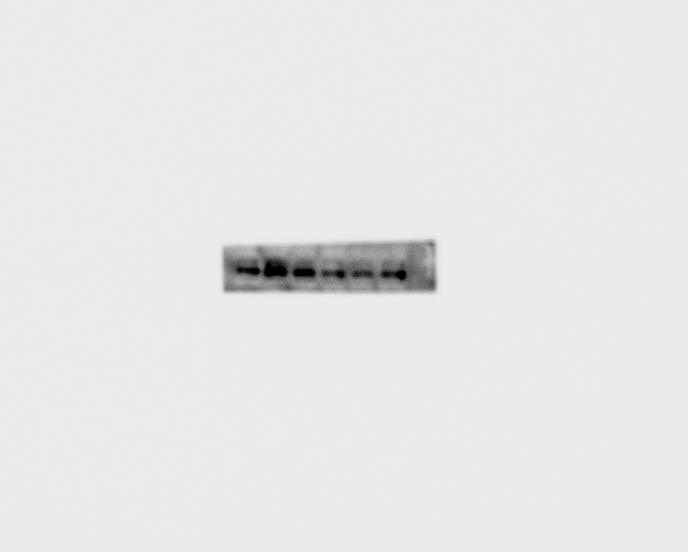

Supplement: Supplementary file 2 [file DataSheet1.ZIP › Original Gel Pictures/Figure 6 E/App-replicate 1.app.tif]

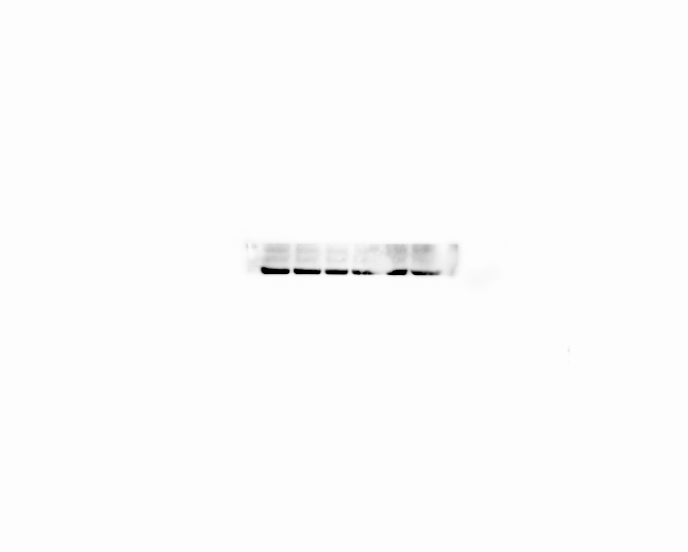

Supplement: Supplementary file 2 [file DataSheet1.ZIP › Original Gel Pictures/Figure 2 J/╬▓-actin-replicate 3.tif]

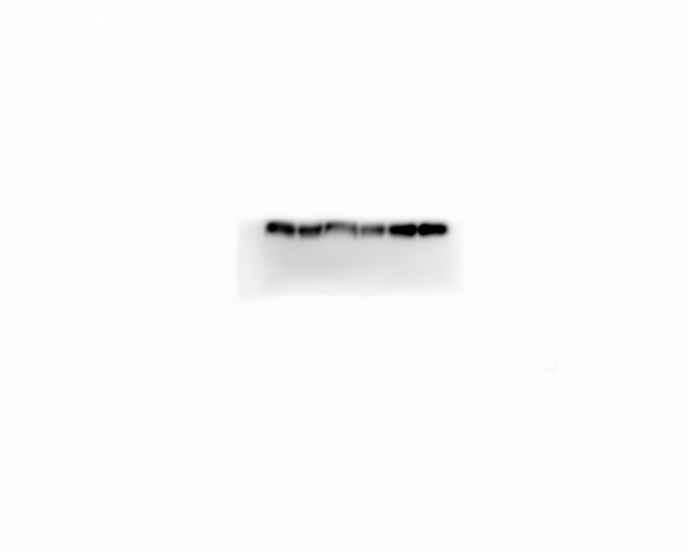

Supplement: Supplementary file 2 [file DataSheet1.ZIP › Original Gel Pictures/Figure 2 J/Syp-replicate 2.tif]

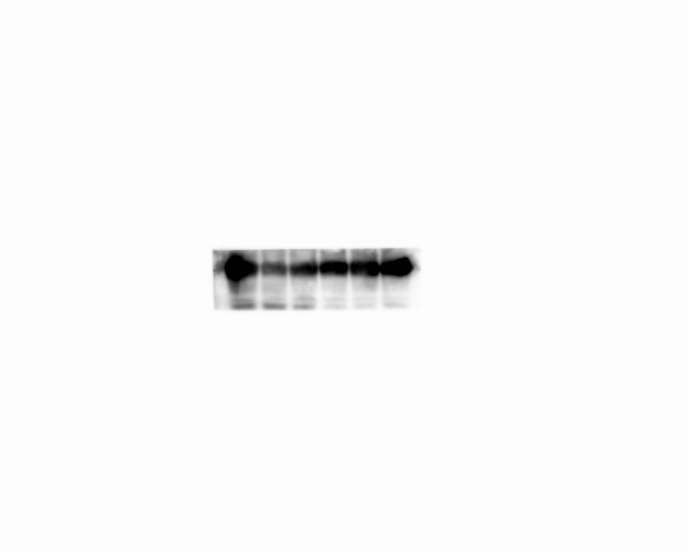

Supplement: Supplementary file 2 [file DataSheet1.ZIP › Original Gel Pictures/Figure 2 J/Syp-replicate 3.tif]

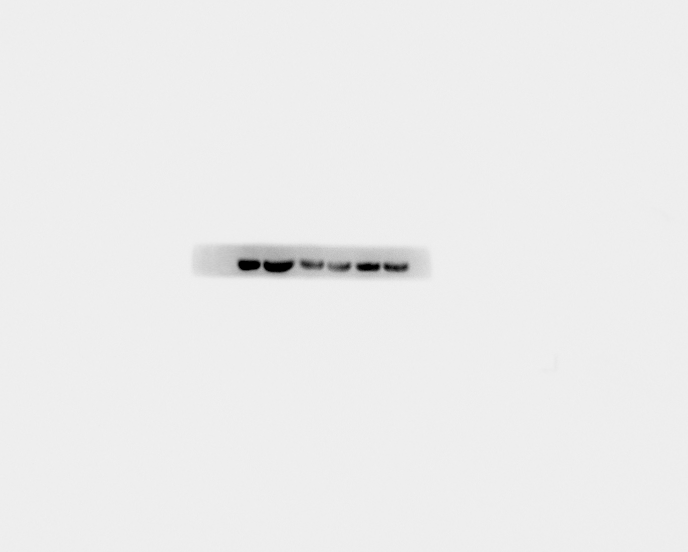

Supplement: Supplementary file 2 [file DataSheet1.ZIP › Original Gel Pictures/Figure 2 J/╬▓-actin-replicate 2.tif]

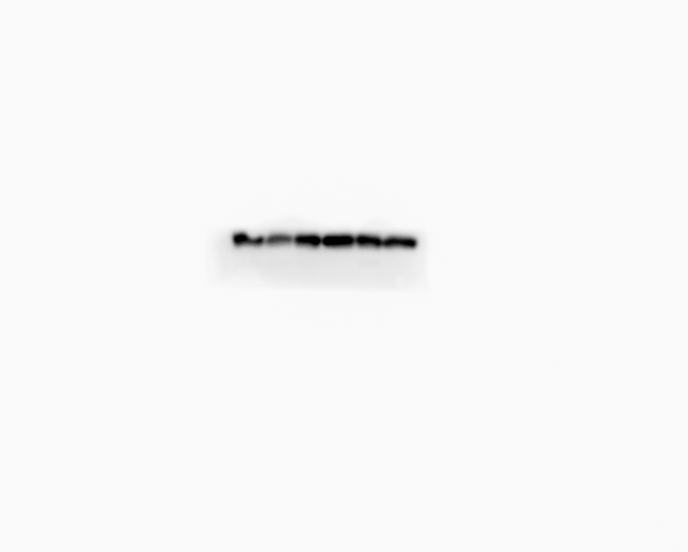

Supplement: Supplementary file 2 [file DataSheet1.ZIP › Original Gel Pictures/Figure 2 J/Syp-replicate 1.tif]

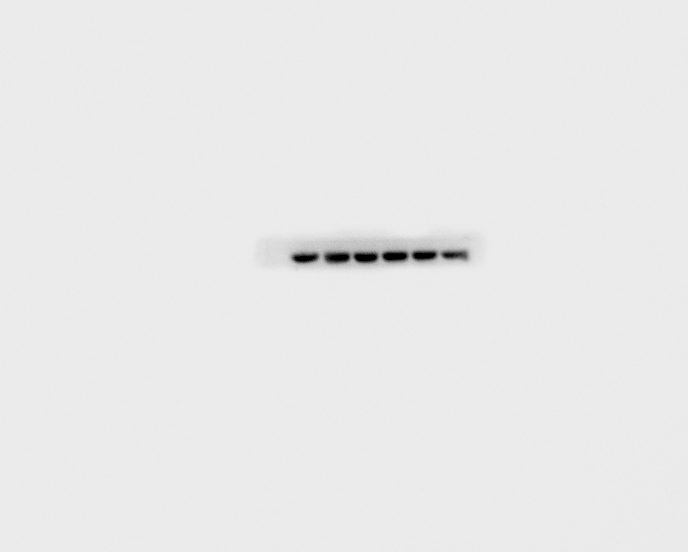

Supplement: Supplementary file 2 [file DataSheet1.ZIP › Original Gel Pictures/Figure 2 J/╬▓-actin-replicate 1.tif]

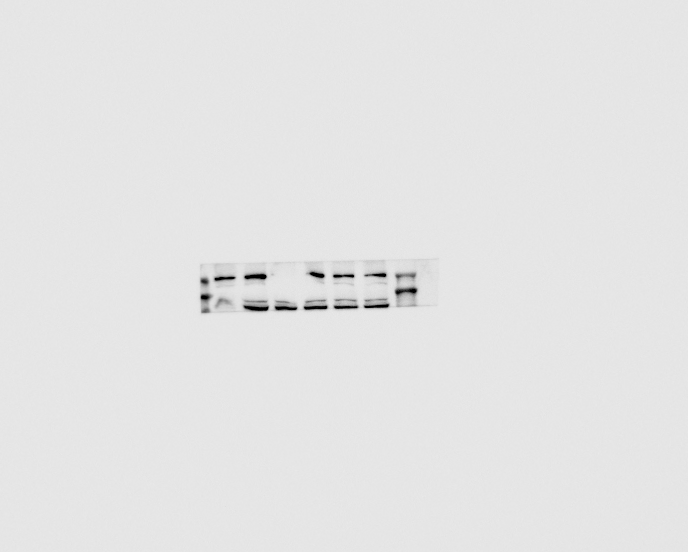

Supplement: Supplementary file 2 [file DataSheet1.ZIP › Original Gel Pictures/Figure 4 I/Tlr4-replicate 2.tif]

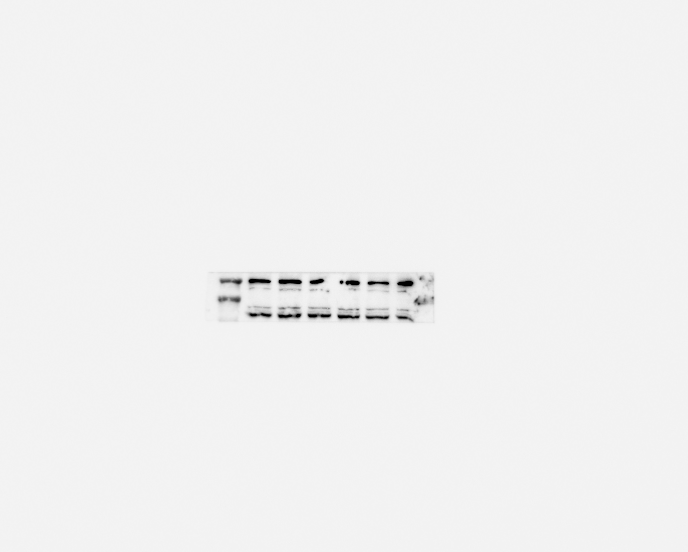

Supplement: Supplementary file 2 [file DataSheet1.ZIP › Original Gel Pictures/Figure 4 I/Tlr4-replicate 3.tif]

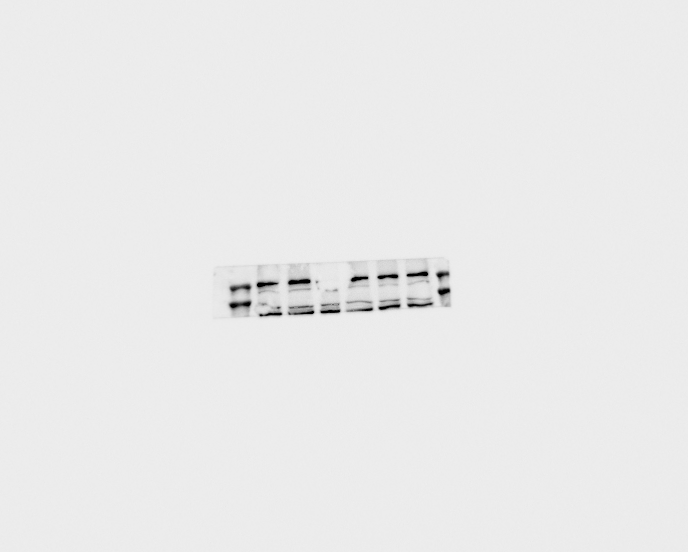

Supplement: Supplementary file 2 [file DataSheet1.ZIP › Original Gel Pictures/Figure 4 I/Tlr4-replicate 1.tif]

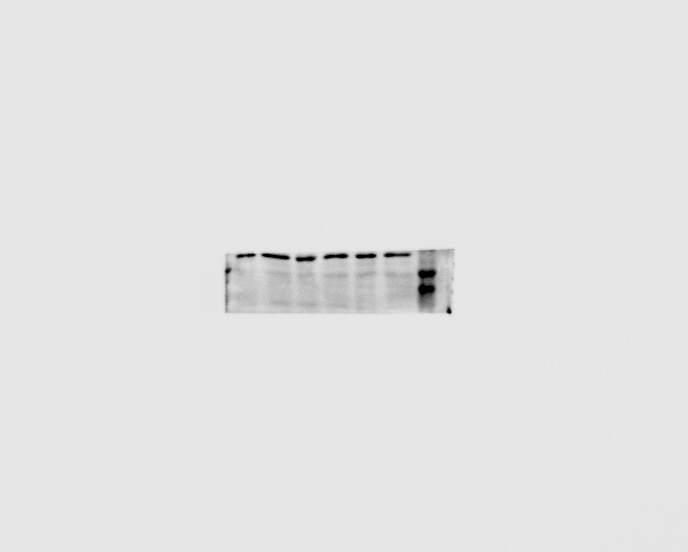

Supplement: Supplementary file 2 [file DataSheet1.ZIP › Original Gel Pictures/Figure 4 I/Myd88-replicate 2.tif]

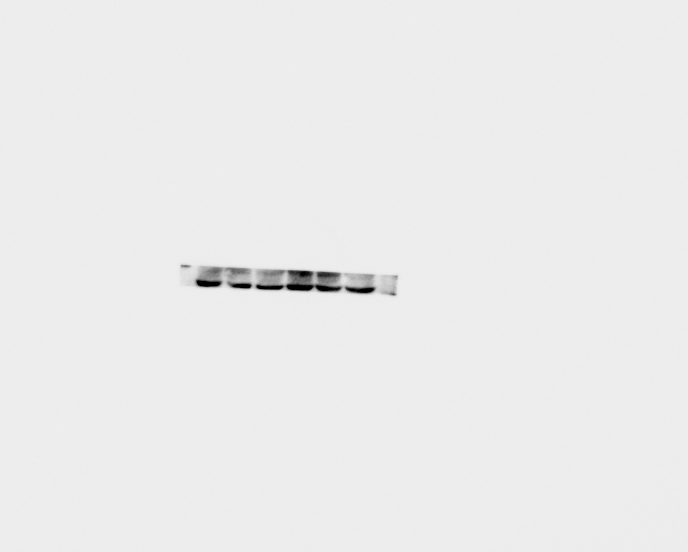

Supplement: Supplementary file 2 [file DataSheet1.ZIP › Original Gel Pictures/Figure 4 I/╬▓-actin-replicate 3.tif]

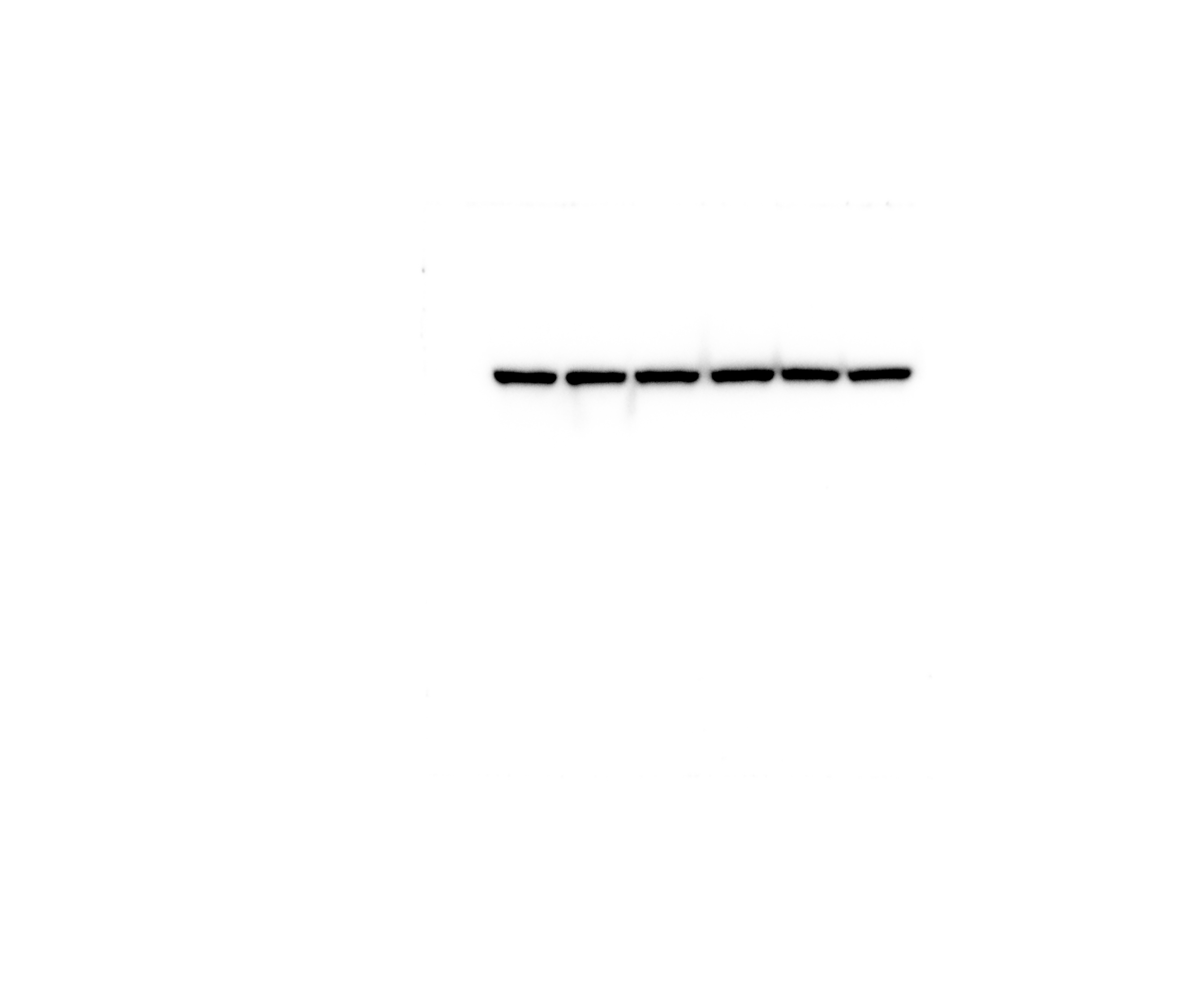

Supplement: Supplementary file 2 [file DataSheet1.ZIP › Original Gel Pictures/Figure 4 I/p65-replicate 1.tif]

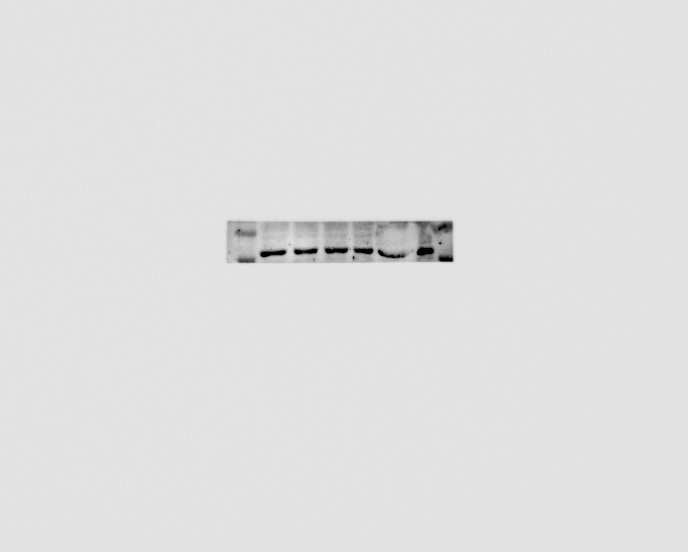

Supplement: Supplementary file 2 [file DataSheet1.ZIP › Original Gel Pictures/Figure 4 I/╬▓-actin-replicate 2.tif]

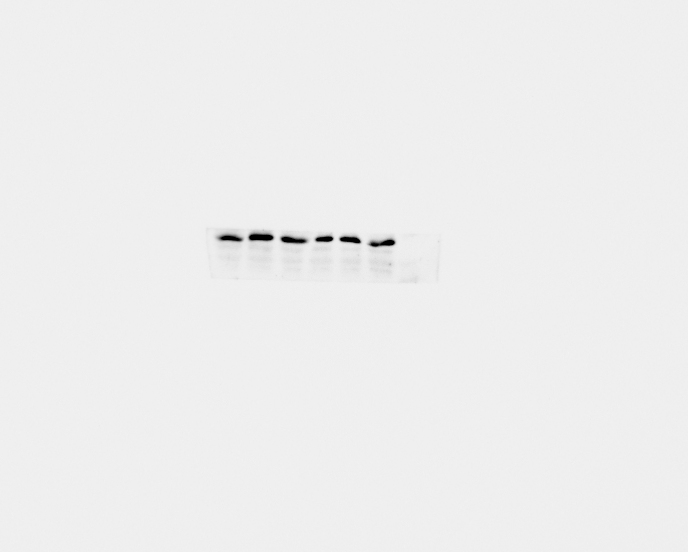

Supplement: Supplementary file 2 [file DataSheet1.ZIP › Original Gel Pictures/Figure 4 I/Myd88-replicate 3.tif]

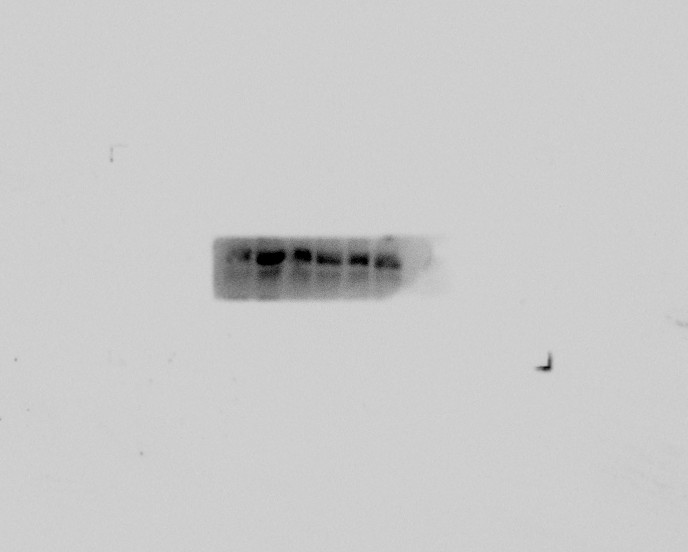

Supplement: Supplementary file 2 [file DataSheet1.ZIP › Original Gel Pictures/Figure 4 I/Myd88-replicate 1.tif]

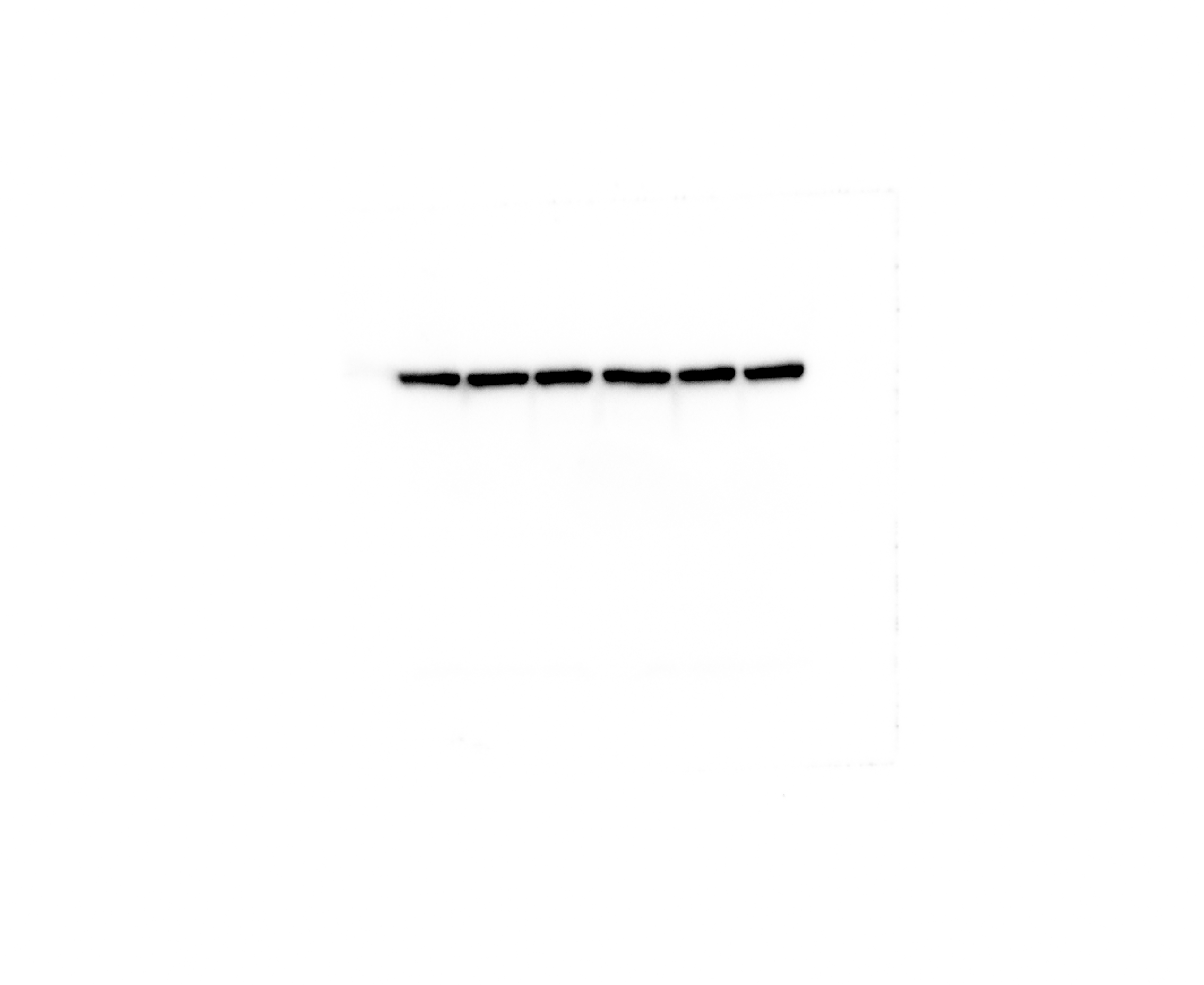

Supplement: Supplementary file 2 [file DataSheet1.ZIP › Original Gel Pictures/Figure 4 I/p65-replicate 2.tif]

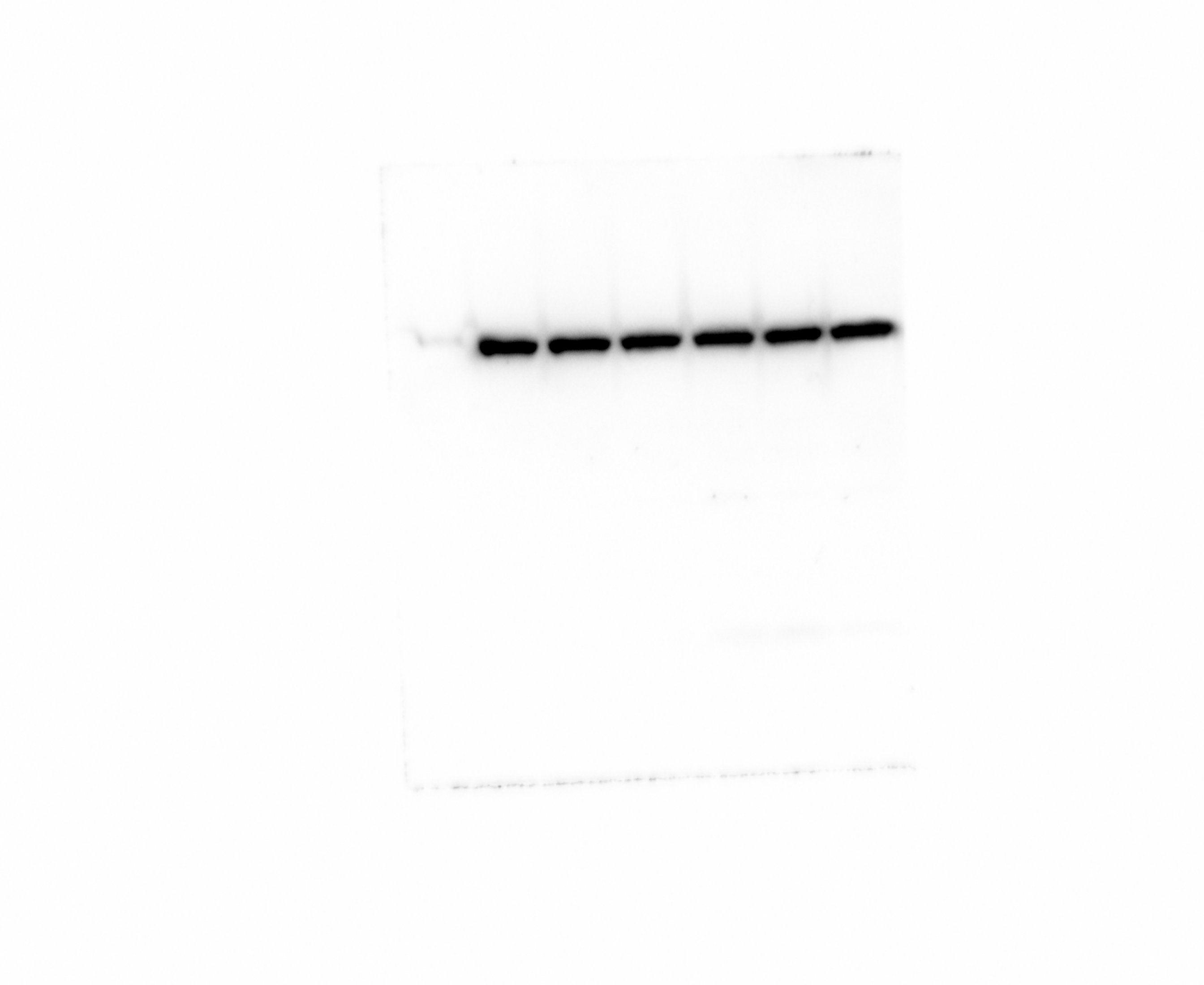

Supplement: Supplementary file 2 [file DataSheet1.ZIP › Original Gel Pictures/Figure 4 I/p65-replicate 3.tif]

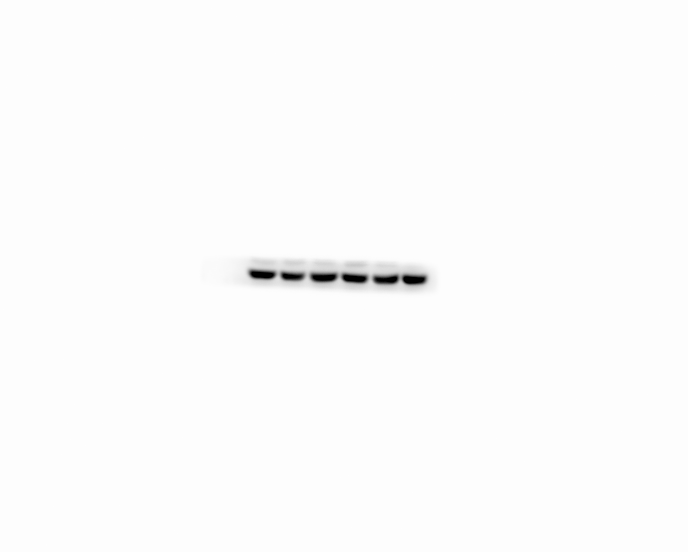

Supplement: Supplementary file 2 [file DataSheet1.ZIP › Original Gel Pictures/Figure 4 I/╬▓-actin-replicate 1.tif]

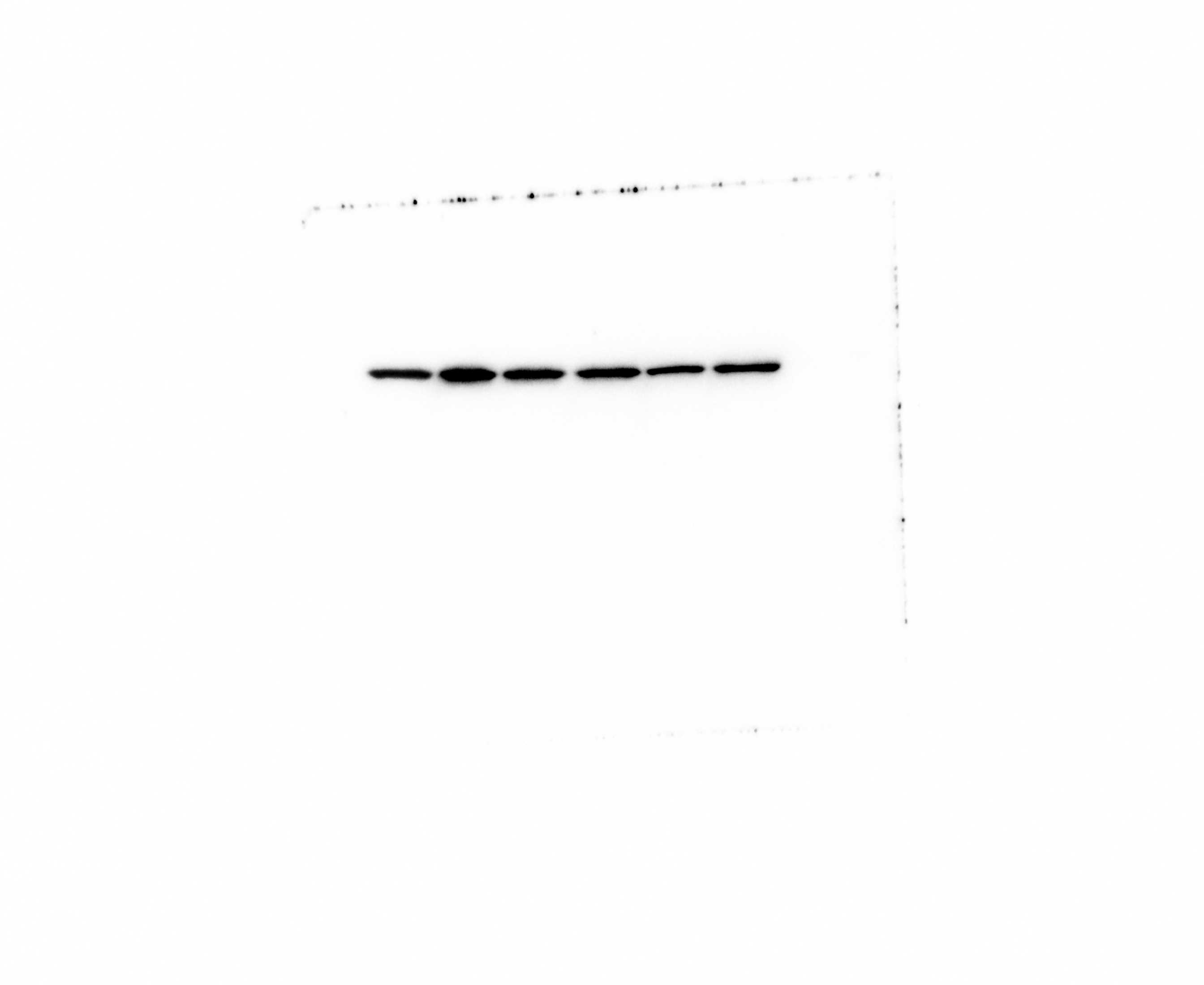

Supplement: Supplementary file 2 [file DataSheet1.ZIP › Original Gel Pictures/Figure 4 I/p-p65-replicate 2.tif]

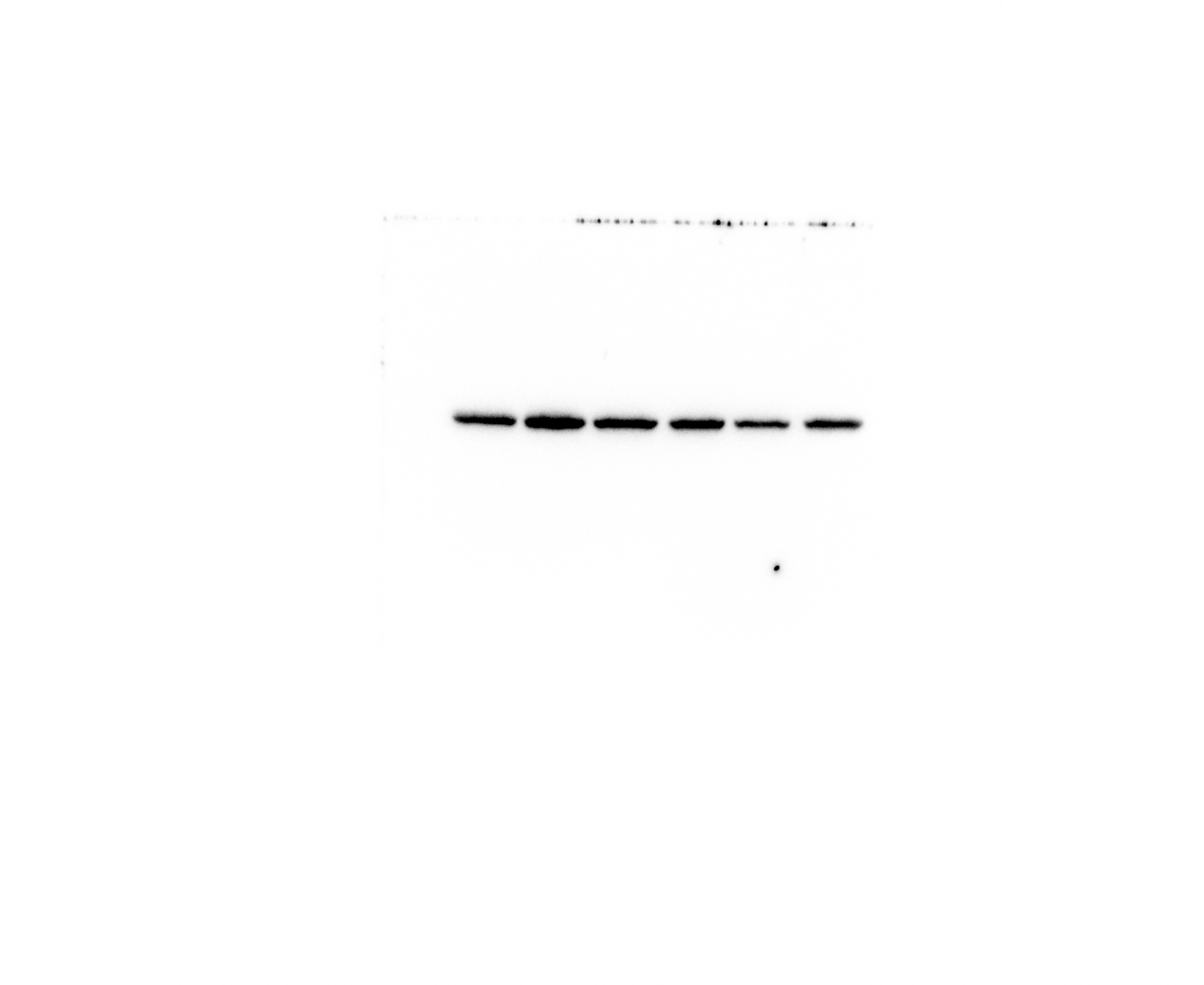

Supplement: Supplementary file 2 [file DataSheet1.ZIP › Original Gel Pictures/Figure 4 I/p-p65-replicate 3.tif]

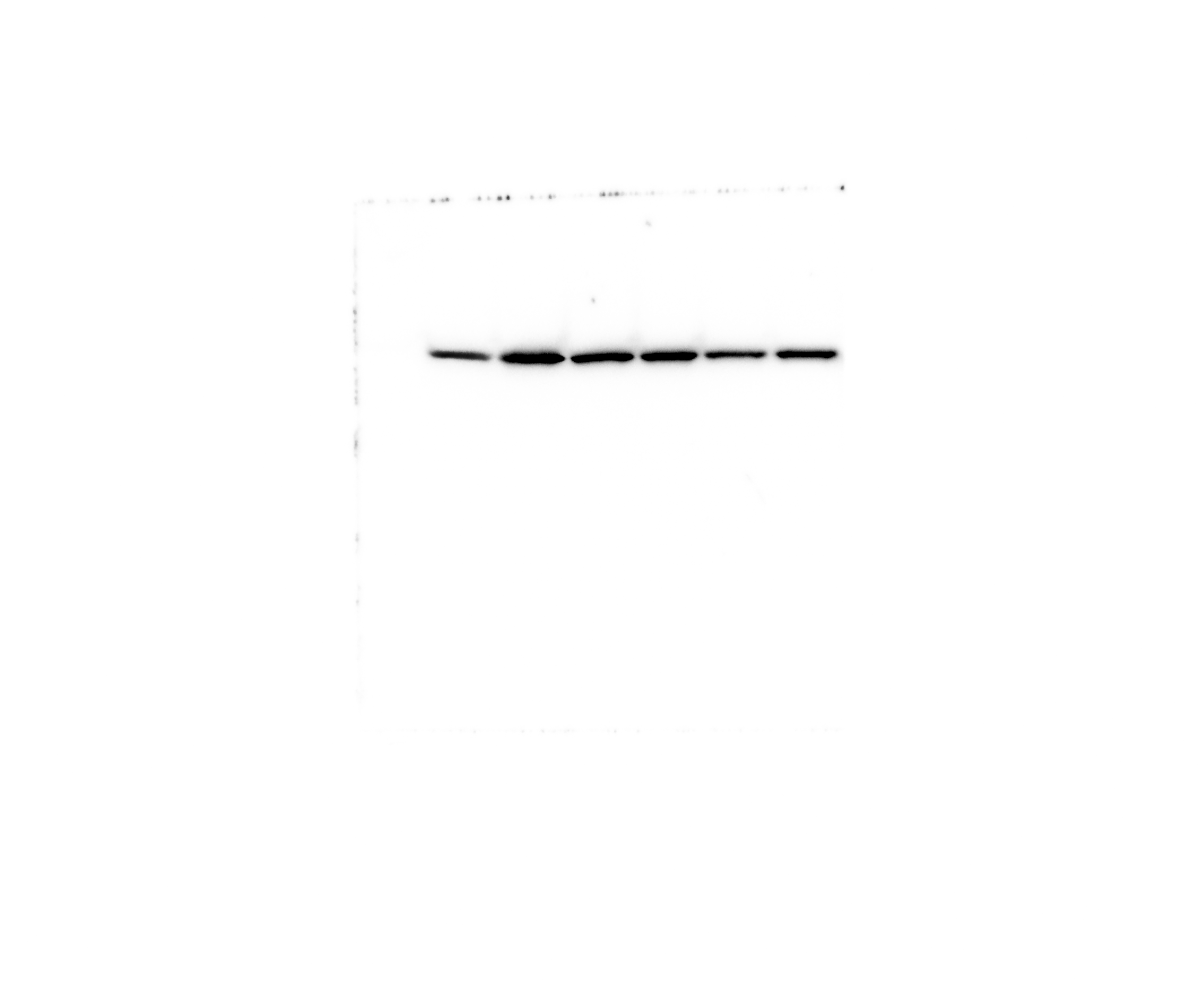

Supplement: Supplementary file 2 [file DataSheet1.ZIP › Original Gel Pictures/Figure 4 I/p-p65-replicate 1.tif]

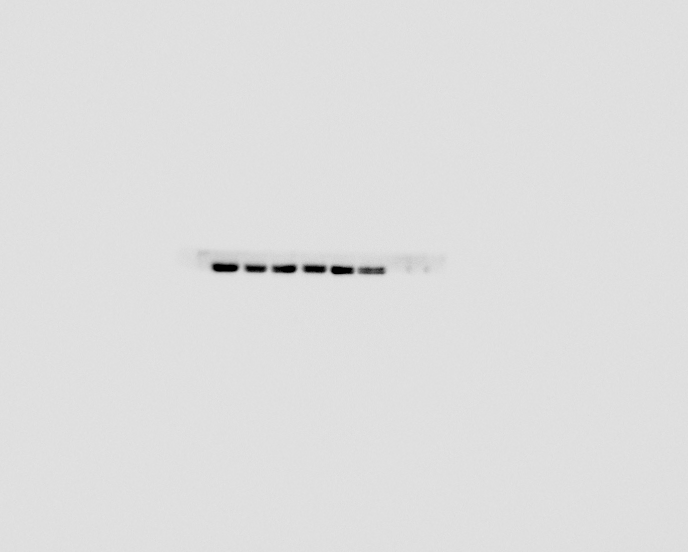

Supplement: Supplementary file 2 [file DataSheet1.ZIP › Original Gel Pictures/Figure 6 M/╬▓-actin-replicate 3.tif]

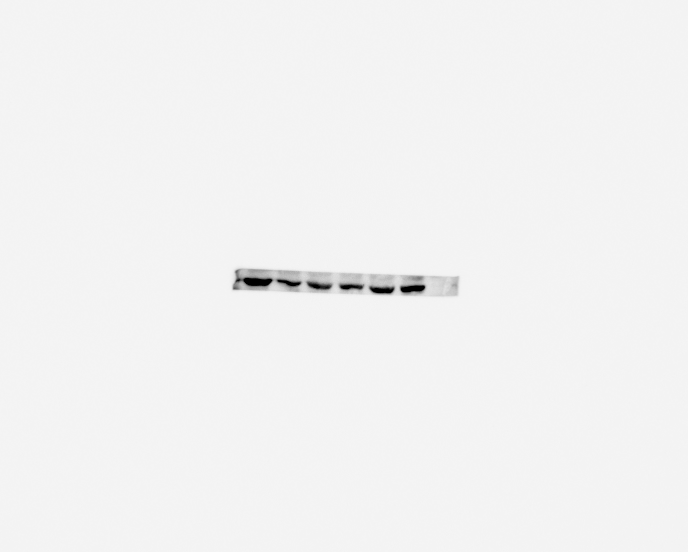

Supplement: Supplementary file 2 [file DataSheet1.ZIP › Original Gel Pictures/Figure 6 M/╬▓-actin-replicate 2.tif]

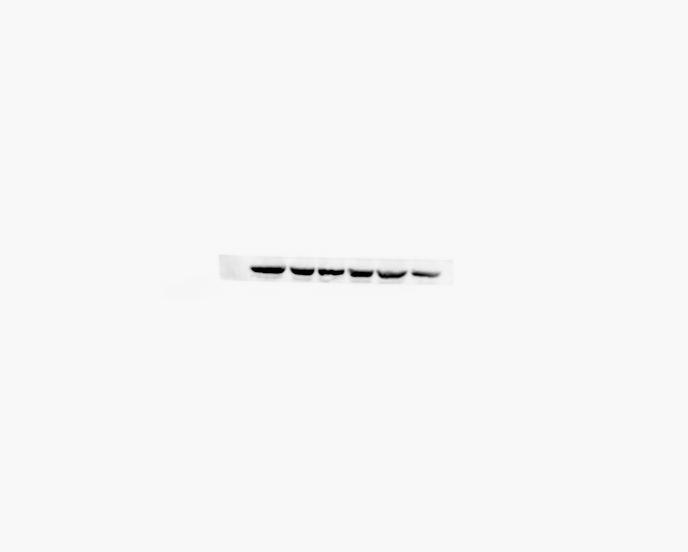

Supplement: Supplementary file 2 [file DataSheet1.ZIP › Original Gel Pictures/Figure 6 M/╬▓-actin-replicate 1.tif]

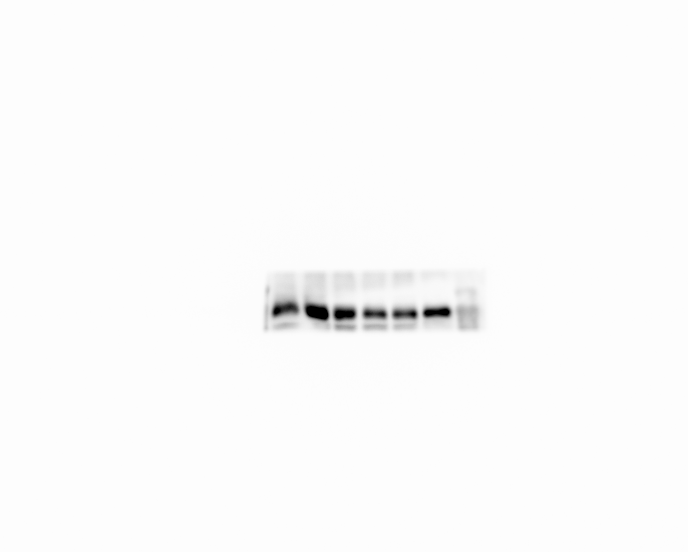

Supplement: Supplementary file 2 [file DataSheet1.ZIP › Original Gel Pictures/Figure 6 M/App-replicate 2.tif]

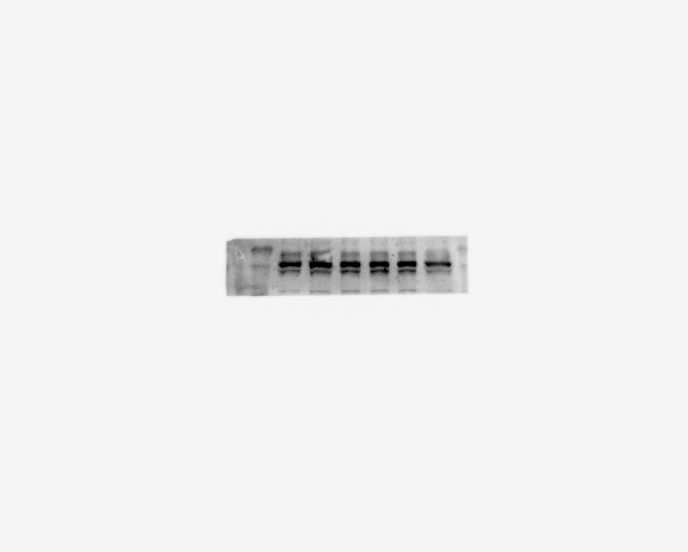

Supplement: Supplementary file 2 [file DataSheet1.ZIP › Original Gel Pictures/Figure 6 M/App-replicate 3.tif]

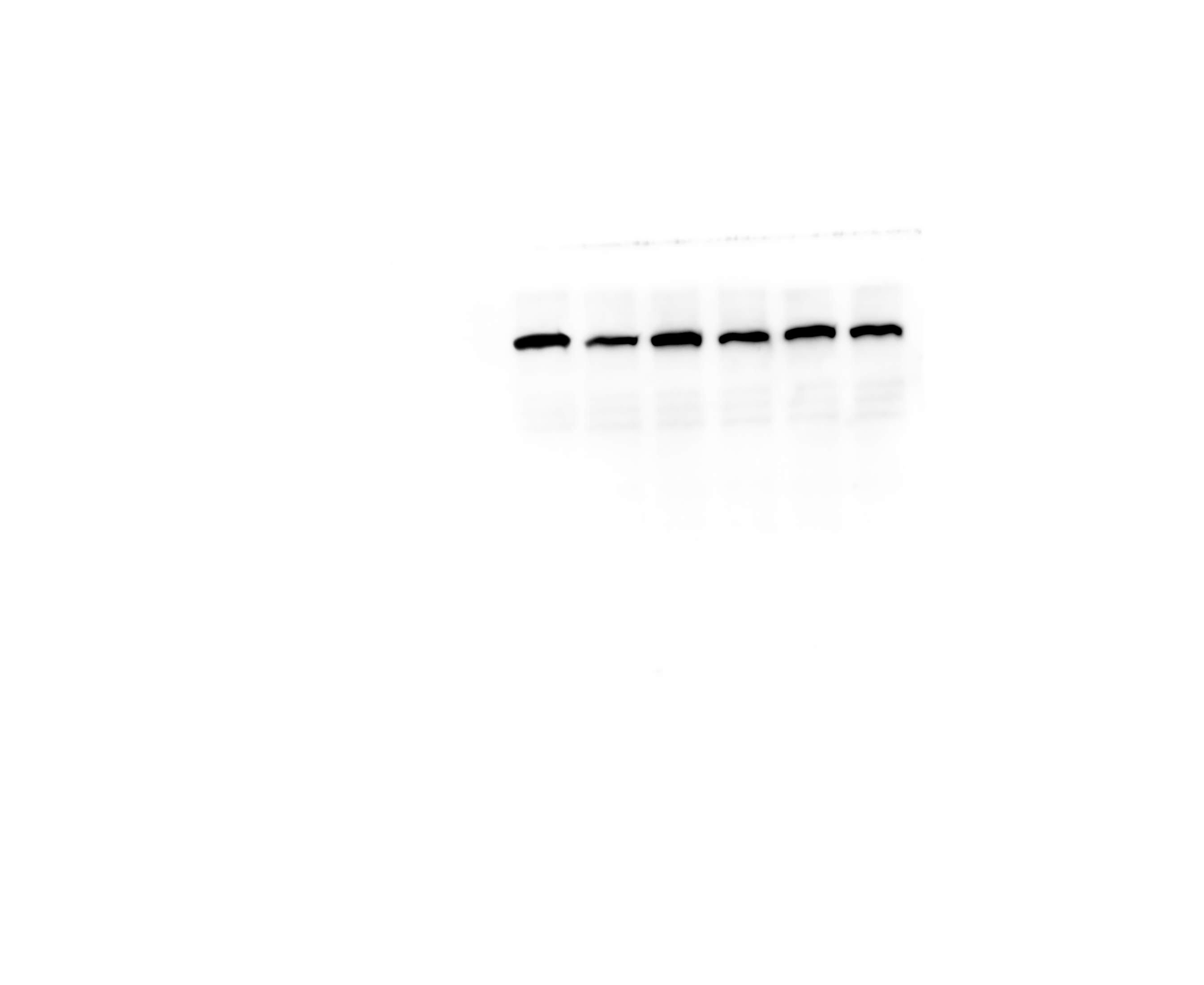

Supplement: Supplementary file 2 [file DataSheet1.ZIP › Original Gel Pictures/Figure 6 M/Tau-replicate 1.tif]

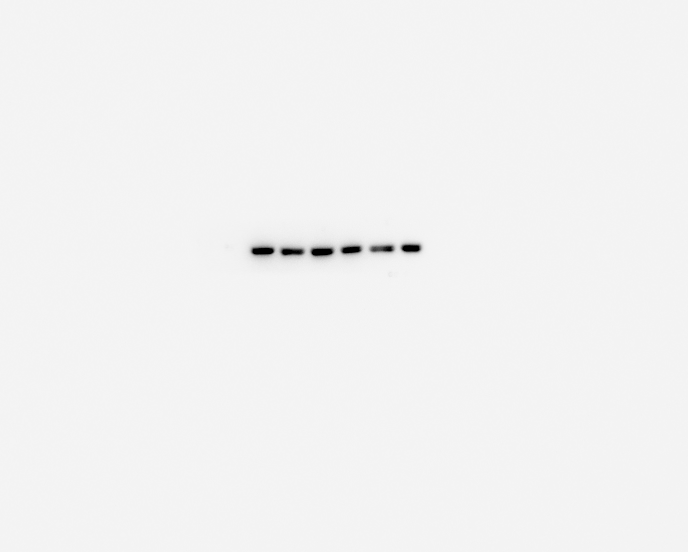

Supplement: Supplementary file 2 [file DataSheet1.ZIP › Original Gel Pictures/Figure 6 M/Tau-replicate 3.tif]

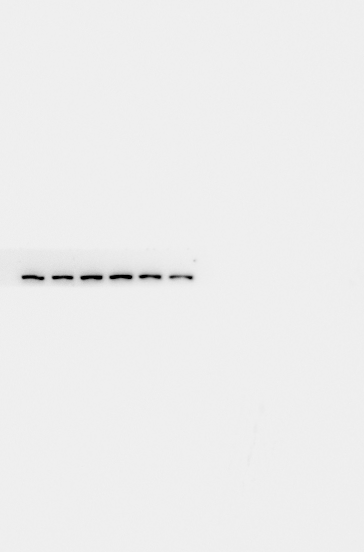

Supplement: Supplementary file 2 [file DataSheet1.ZIP › Original Gel Pictures/Figure 6 M/Tau-replicate 2.tif]

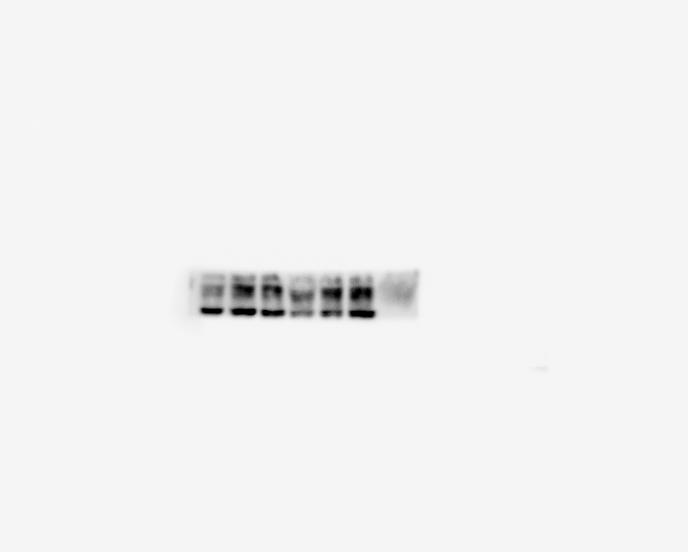

Supplement: Supplementary file 2 [file DataSheet1.ZIP › Original Gel Pictures/Figure 6 M/p-Tau396-replicate 1.tif]

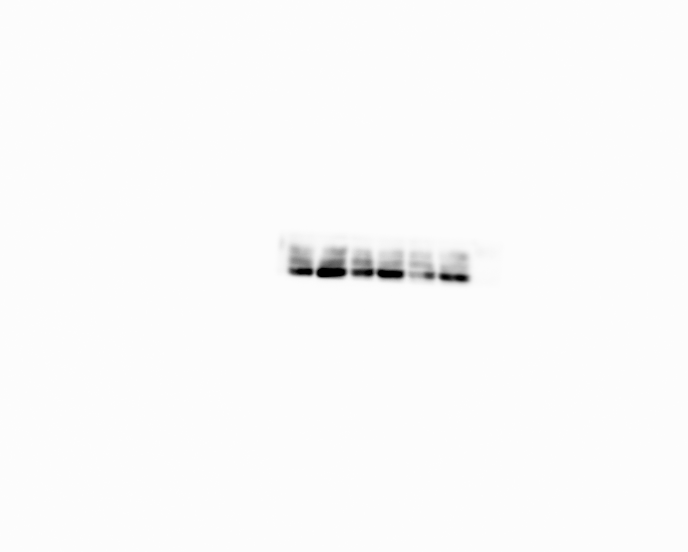

Supplement: Supplementary file 2 [file DataSheet1.ZIP › Original Gel Pictures/Figure 6 M/p-Tau396-replicate 2.tif]

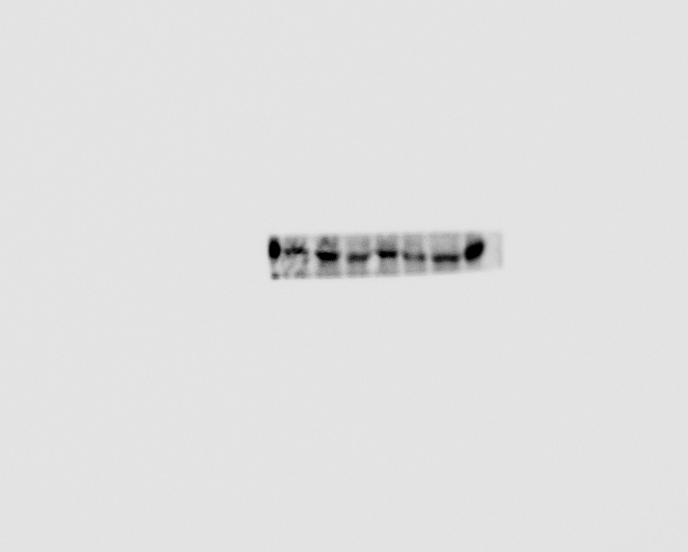

Supplement: Supplementary file 2 [file DataSheet1.ZIP › Original Gel Pictures/Figure 6 M/p-Tau396-replicate 3.tif]

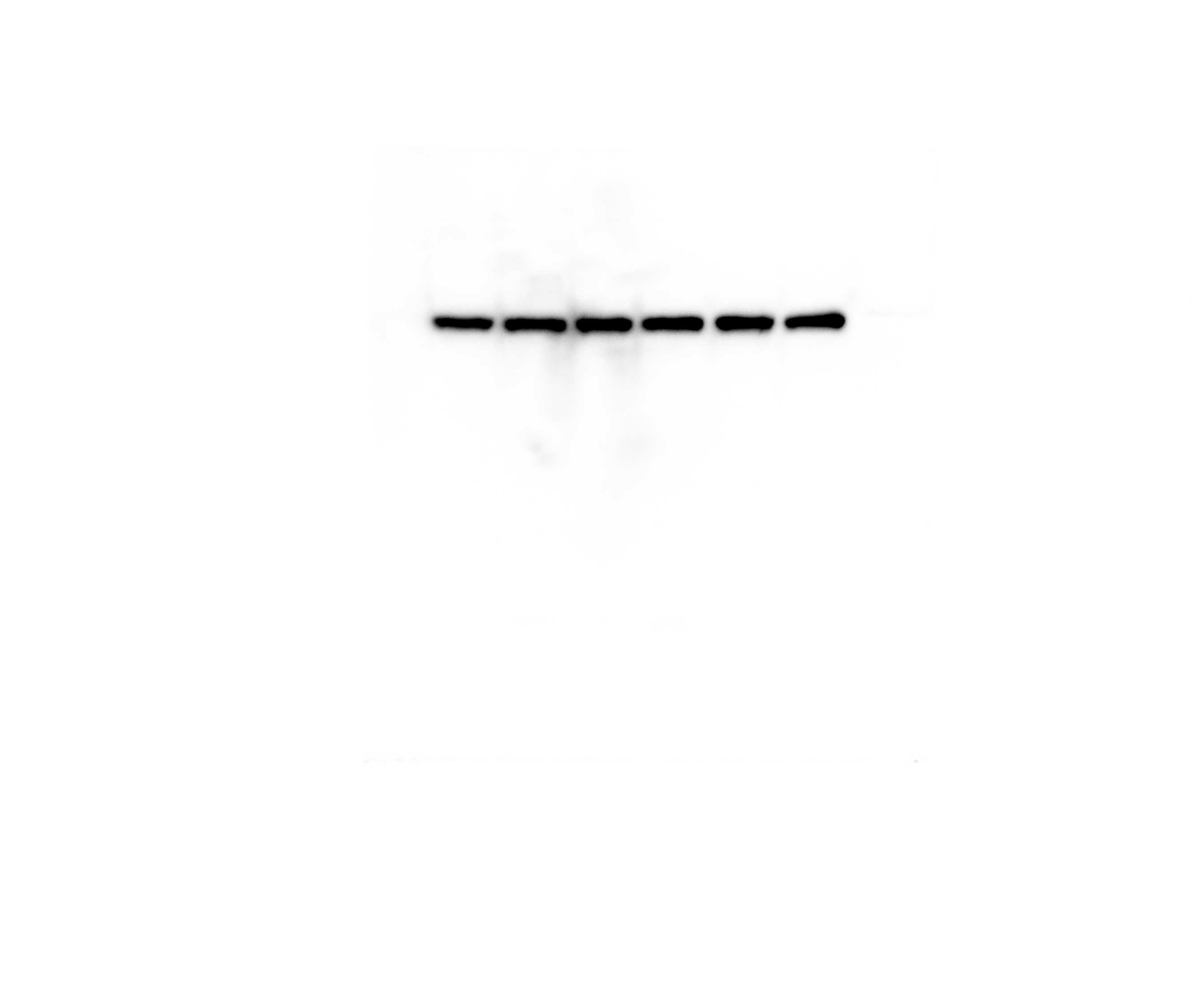

Supplement: Supplementary file 2 [file DataSheet1.ZIP › Original Gel Pictures/Figure 5 G/p65-replicate 1.tif]

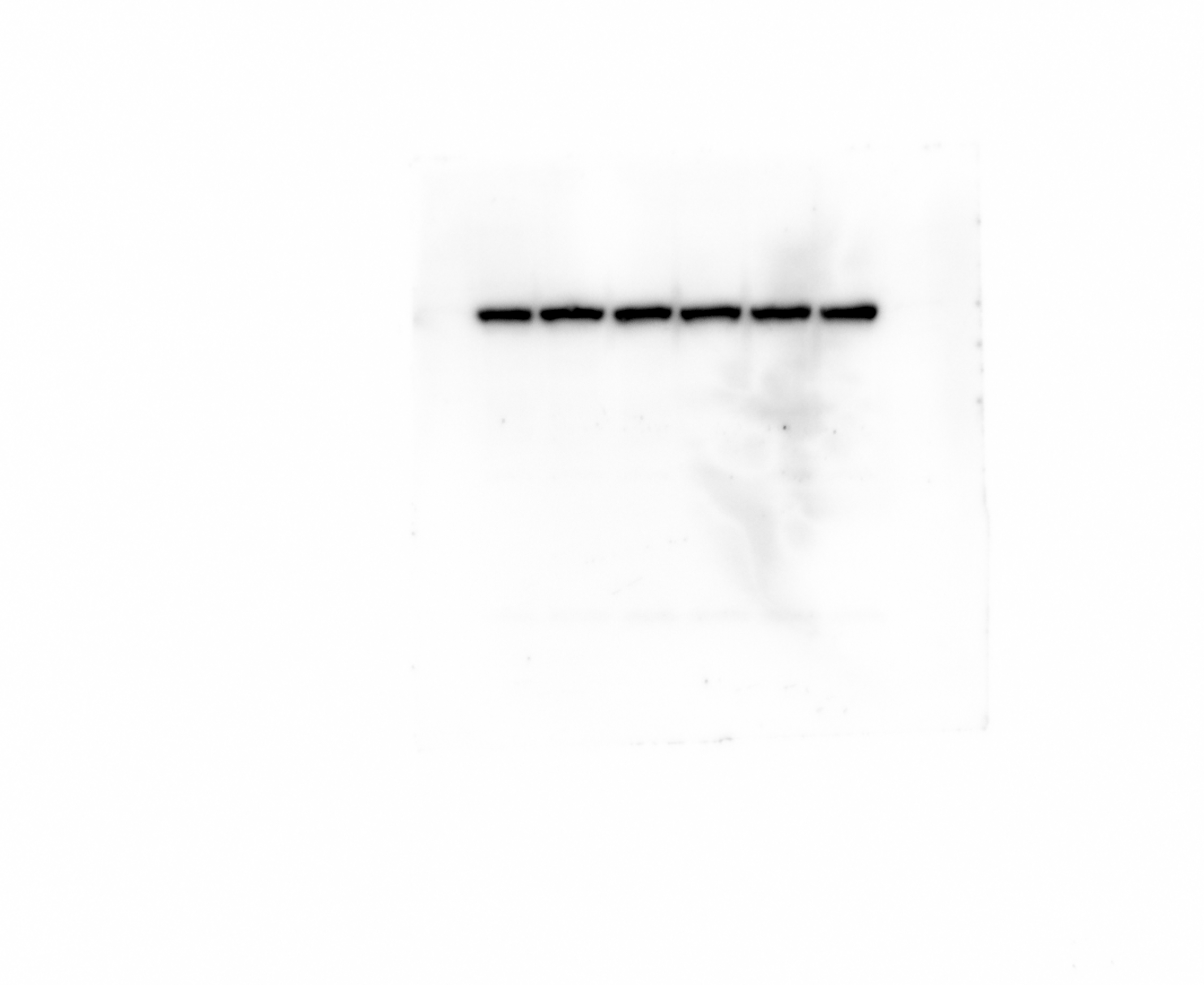

Supplement: Supplementary file 2 [file DataSheet1.ZIP › Original Gel Pictures/Figure 5 G/p65-replicate 2.tif]

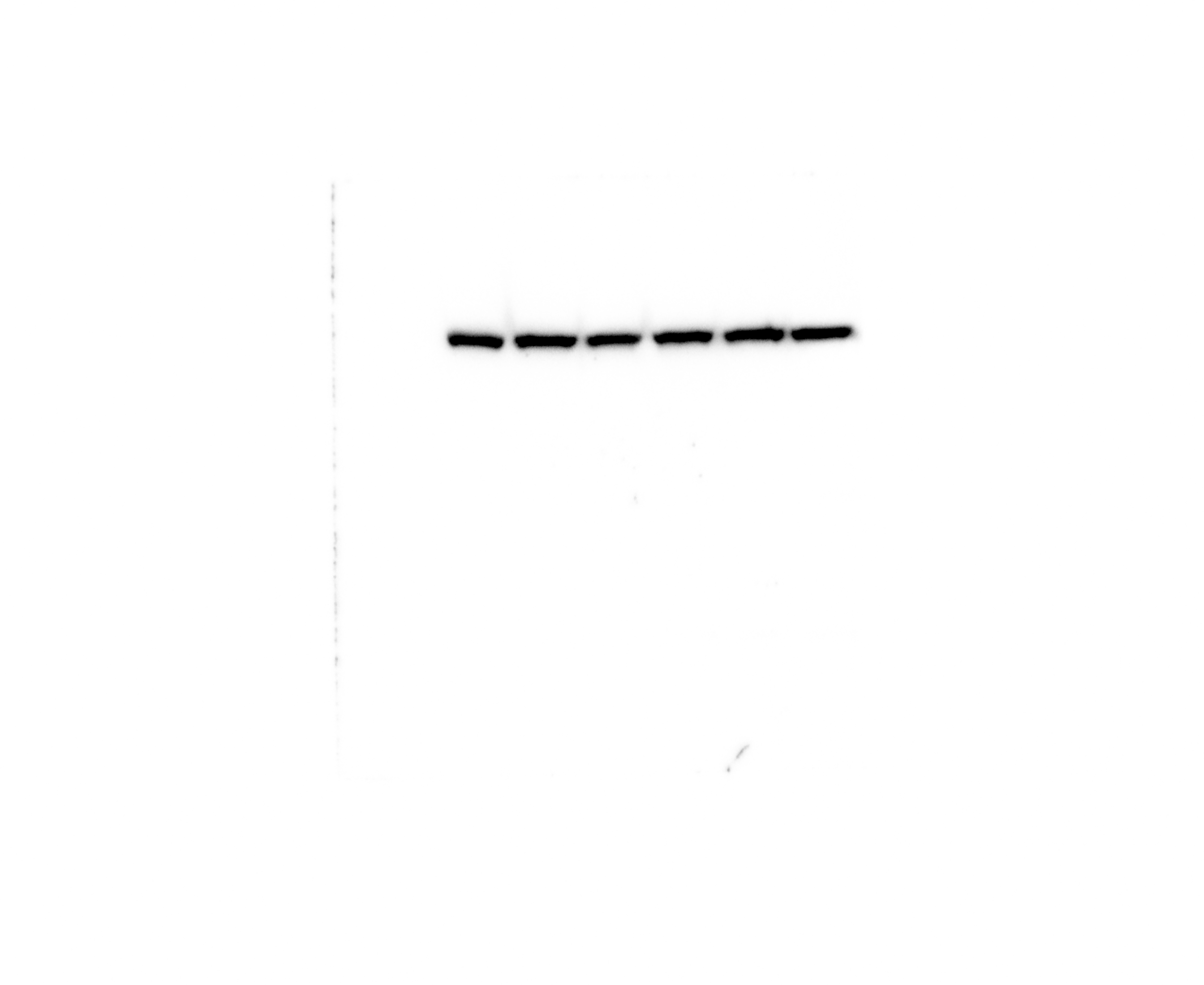

Supplement: Supplementary file 2 [file DataSheet1.ZIP › Original Gel Pictures/Figure 5 G/p65-replicate 3.tif]

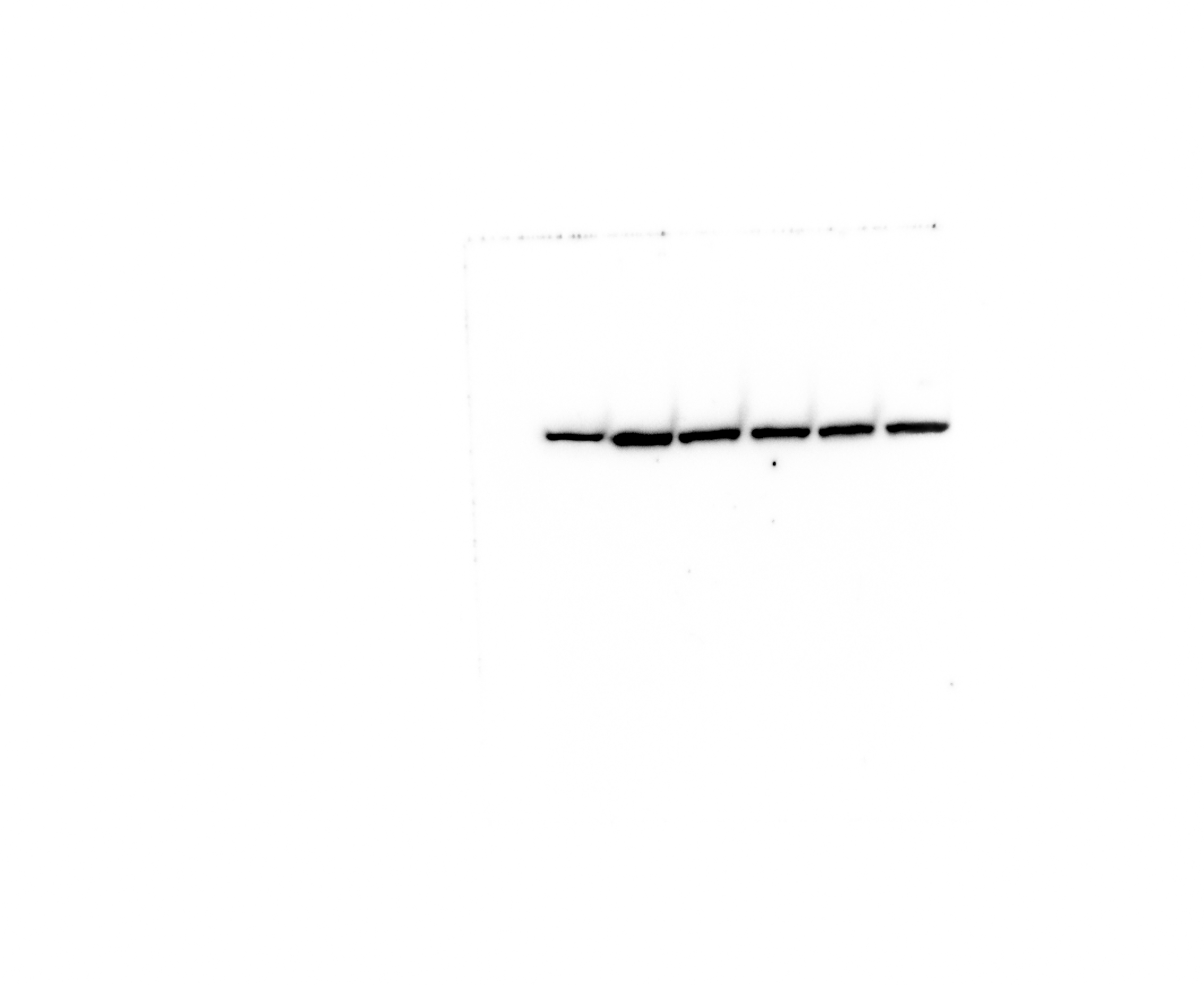

Supplement: Supplementary file 2 [file DataSheet1.ZIP › Original Gel Pictures/Figure 5 G/p-p65-replicate 2.tif]

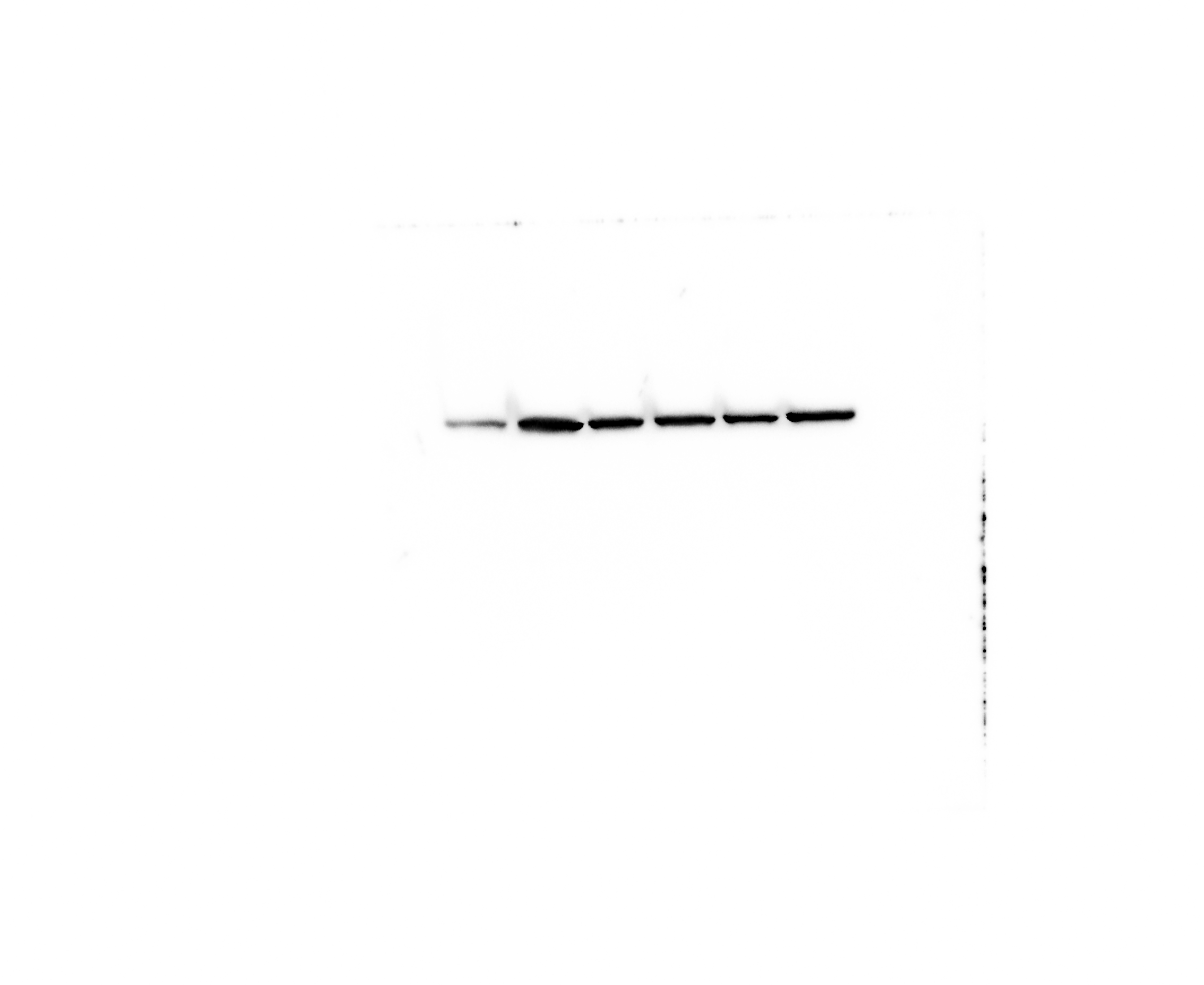

Supplement: Supplementary file 2 [file DataSheet1.ZIP › Original Gel Pictures/Figure 5 G/p-p65-replicate 3.tif]

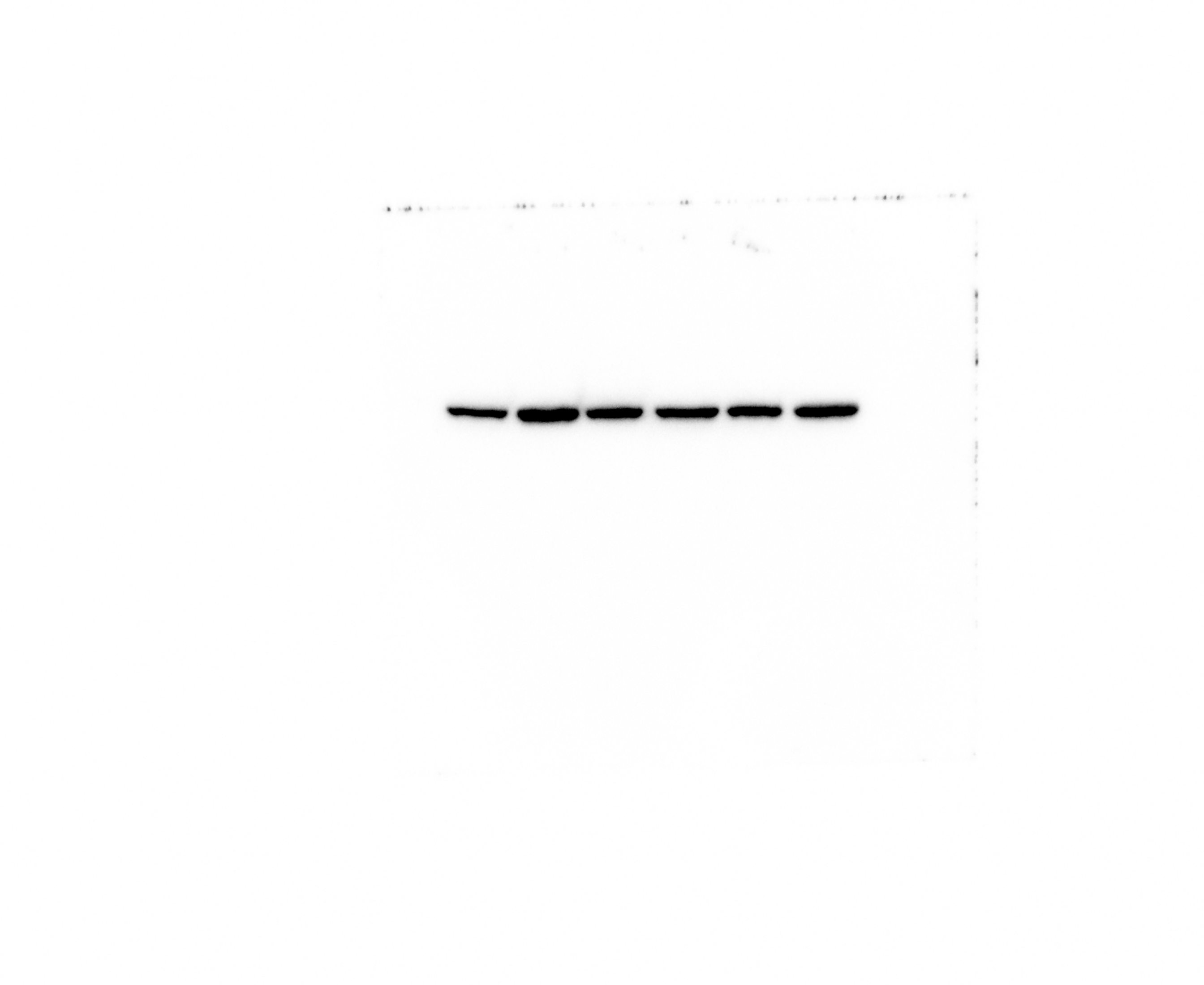

Supplement: Supplementary file 2 [file DataSheet1.ZIP › Original Gel Pictures/Figure 5 G/p-p65-replicate 1.tif]

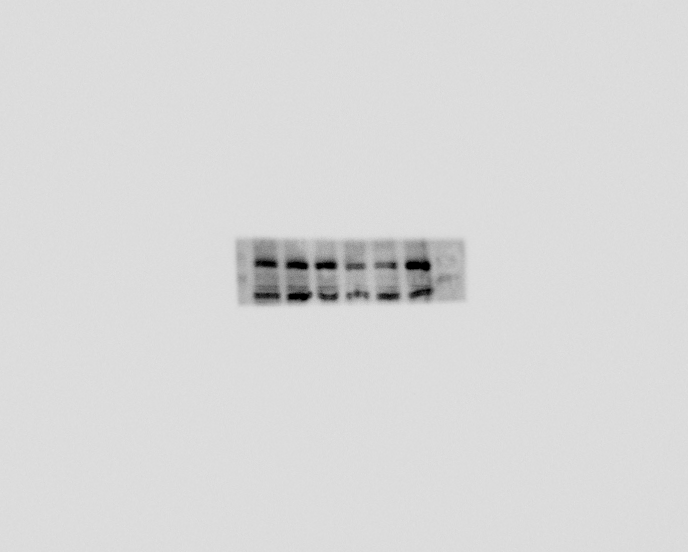

Supplement: Supplementary file 2 [file DataSheet1.ZIP › Original Gel Pictures/Figure 6 I/Tlr4-replicate 2.tif]

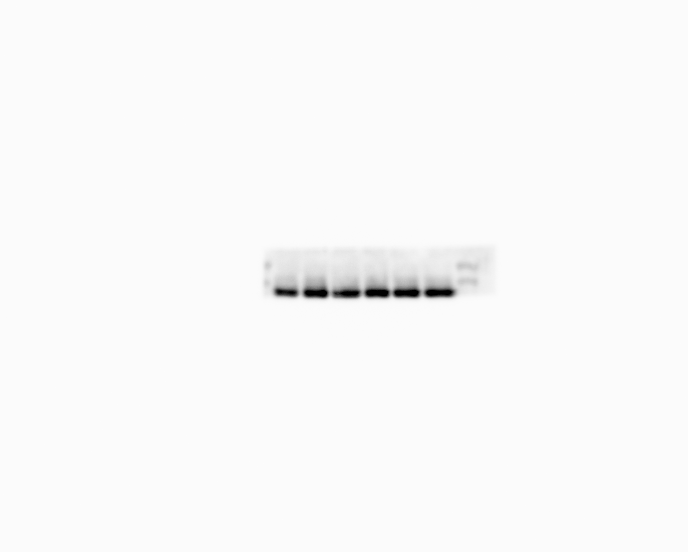

Supplement: Supplementary file 2 [file DataSheet1.ZIP › Original Gel Pictures/Figure 6 I/Tlr4-replicate 3.tif]

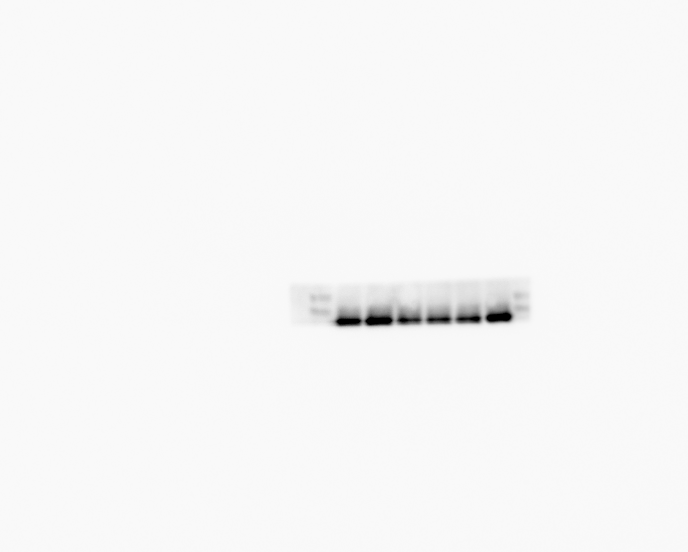

Supplement: Supplementary file 2 [file DataSheet1.ZIP › Original Gel Pictures/Figure 6 I/Tlr4-replicate 1.tif]

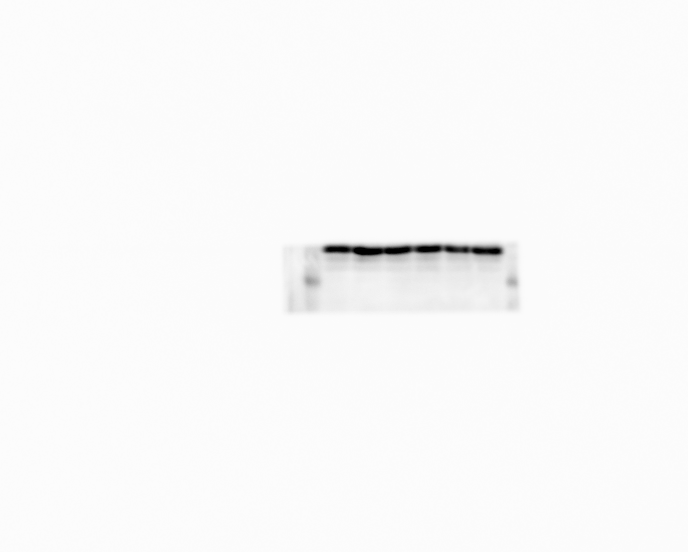

Supplement: Supplementary file 2 [file DataSheet1.ZIP › Original Gel Pictures/Figure 6 I/Myd88-replicate 2.tif]

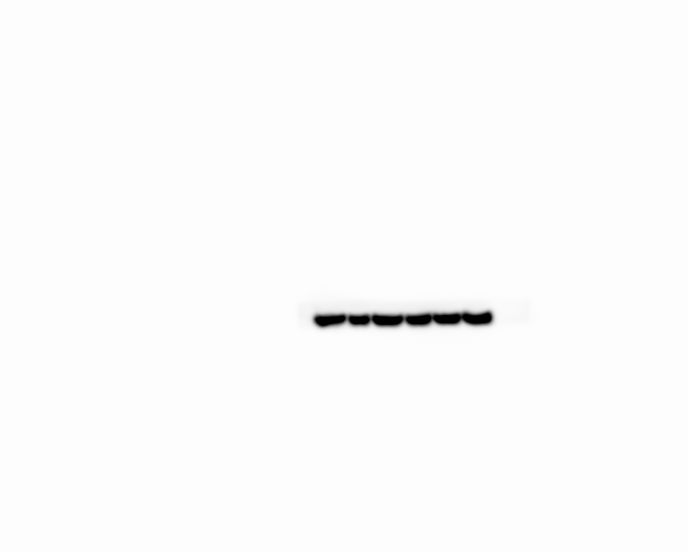

Supplement: Supplementary file 2 [file DataSheet1.ZIP › Original Gel Pictures/Figure 6 I/╬▓-actin-replicate 3.tif]

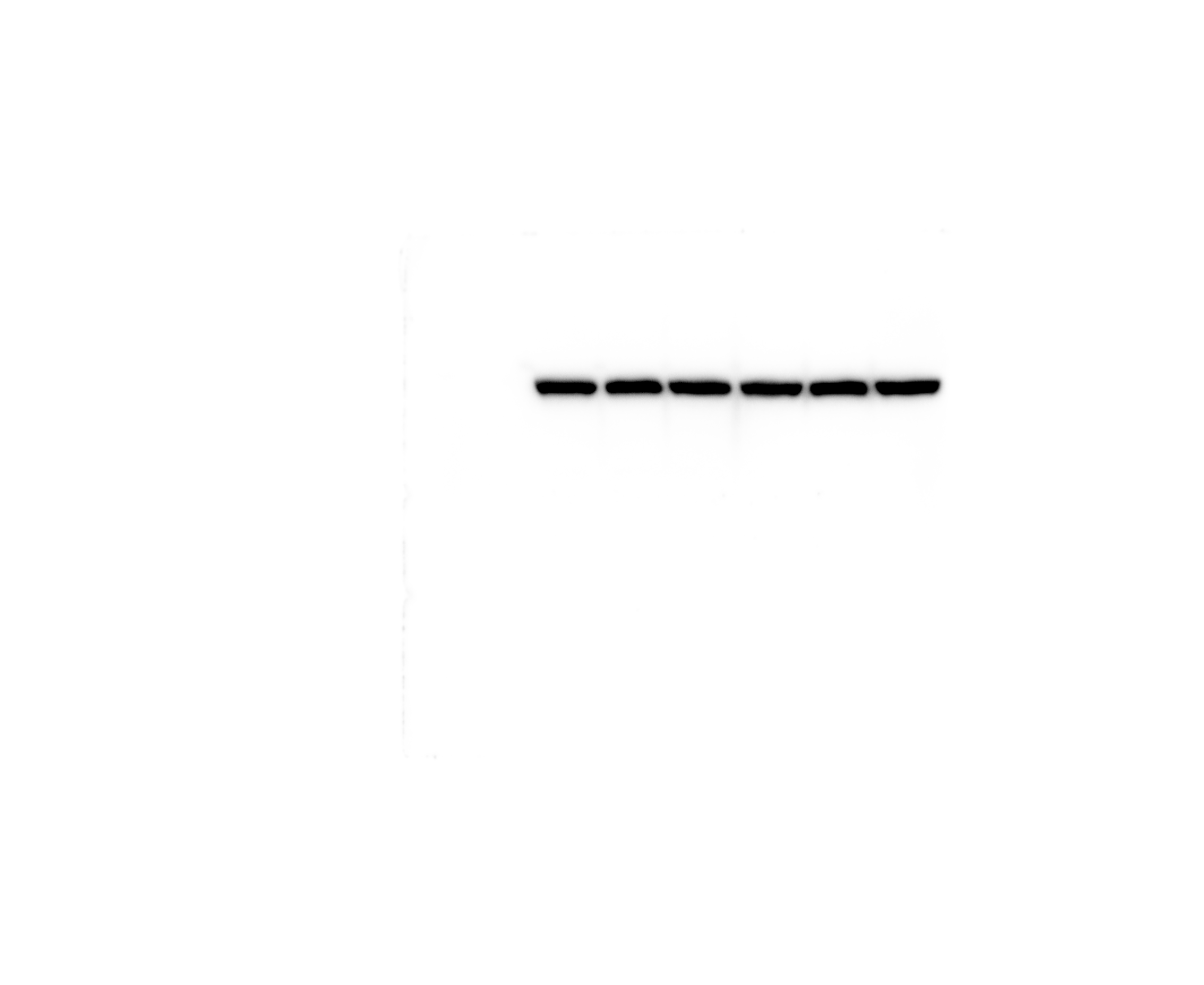

Supplement: Supplementary file 2 [file DataSheet1.ZIP › Original Gel Pictures/Figure 6 I/p65-replicate 1.tif]

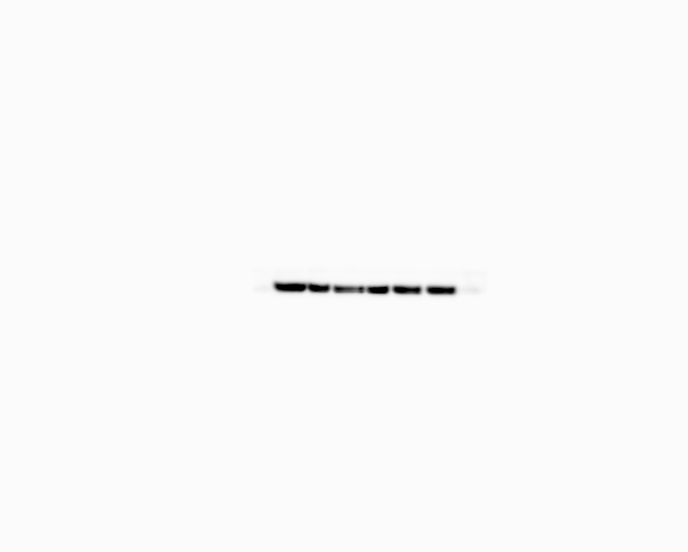

Supplement: Supplementary file 2 [file DataSheet1.ZIP › Original Gel Pictures/Figure 6 I/╬▓-actin-replicate 2.tif]

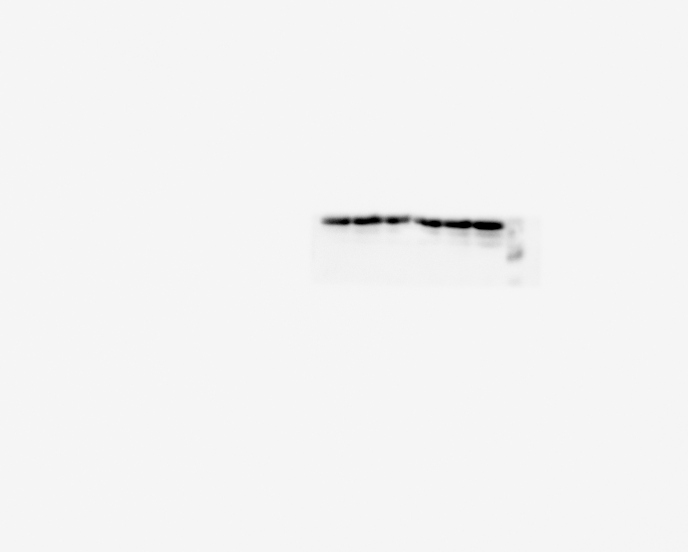

Supplement: Supplementary file 2 [file DataSheet1.ZIP › Original Gel Pictures/Figure 6 I/Myd88-replicate 3.tif]

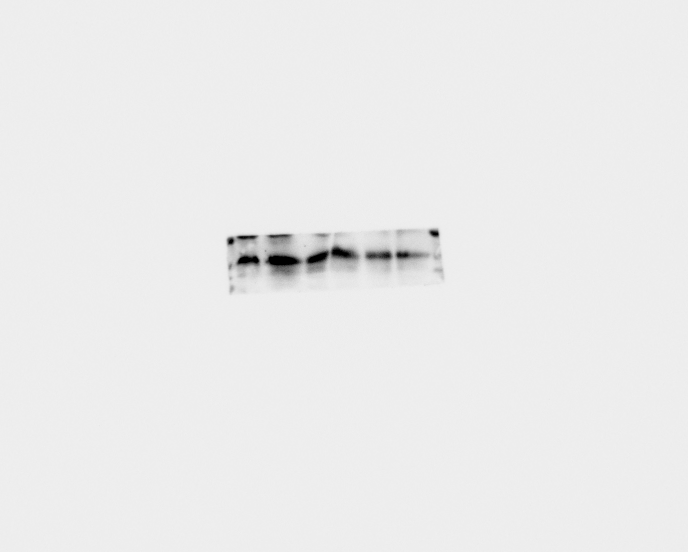

Supplement: Supplementary file 2 [file DataSheet1.ZIP › Original Gel Pictures/Figure 6 I/Myd88-replicate 1.tif]

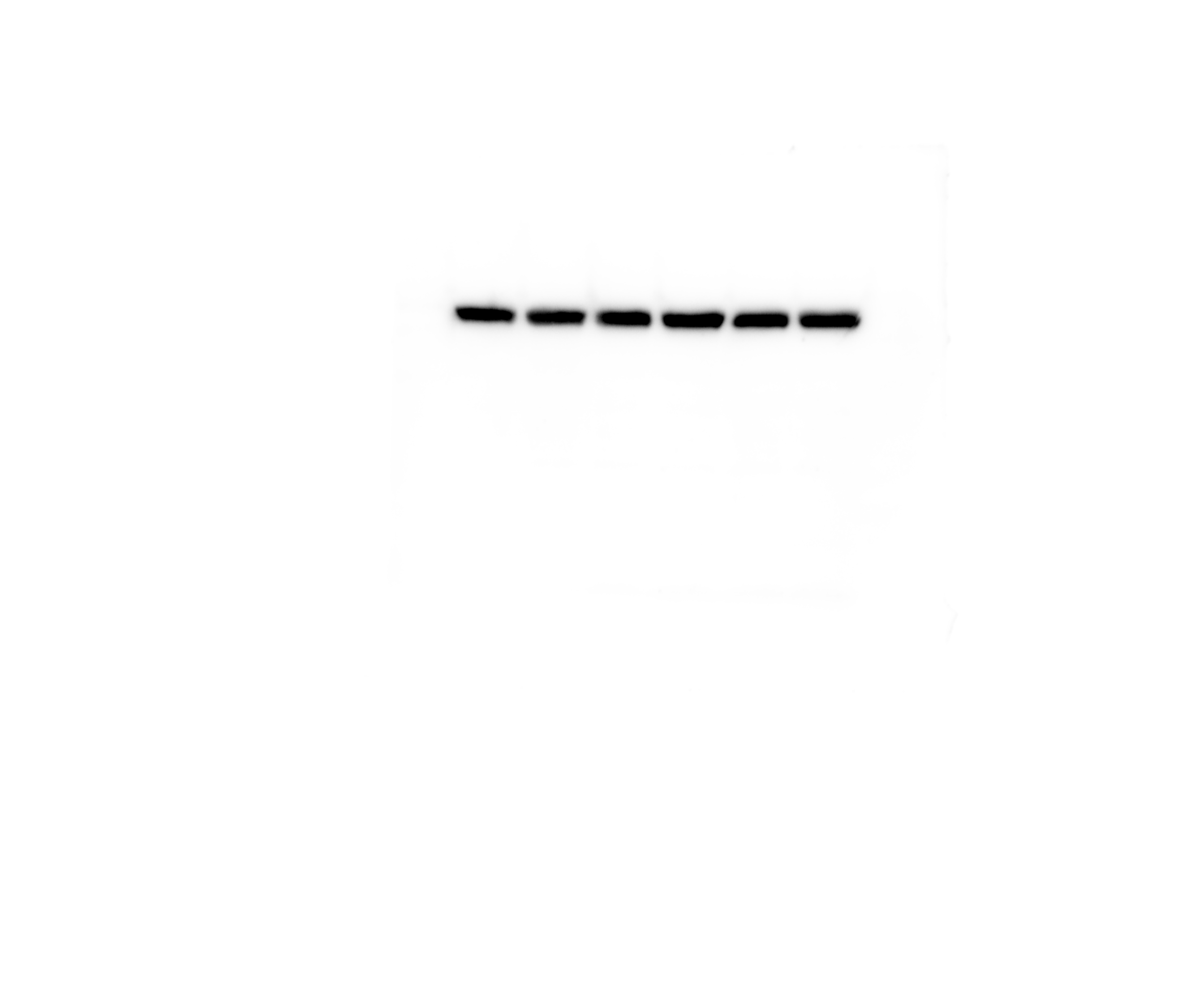

Supplement: Supplementary file 2 [file DataSheet1.ZIP › Original Gel Pictures/Figure 6 I/p65-replicate 2.tif]

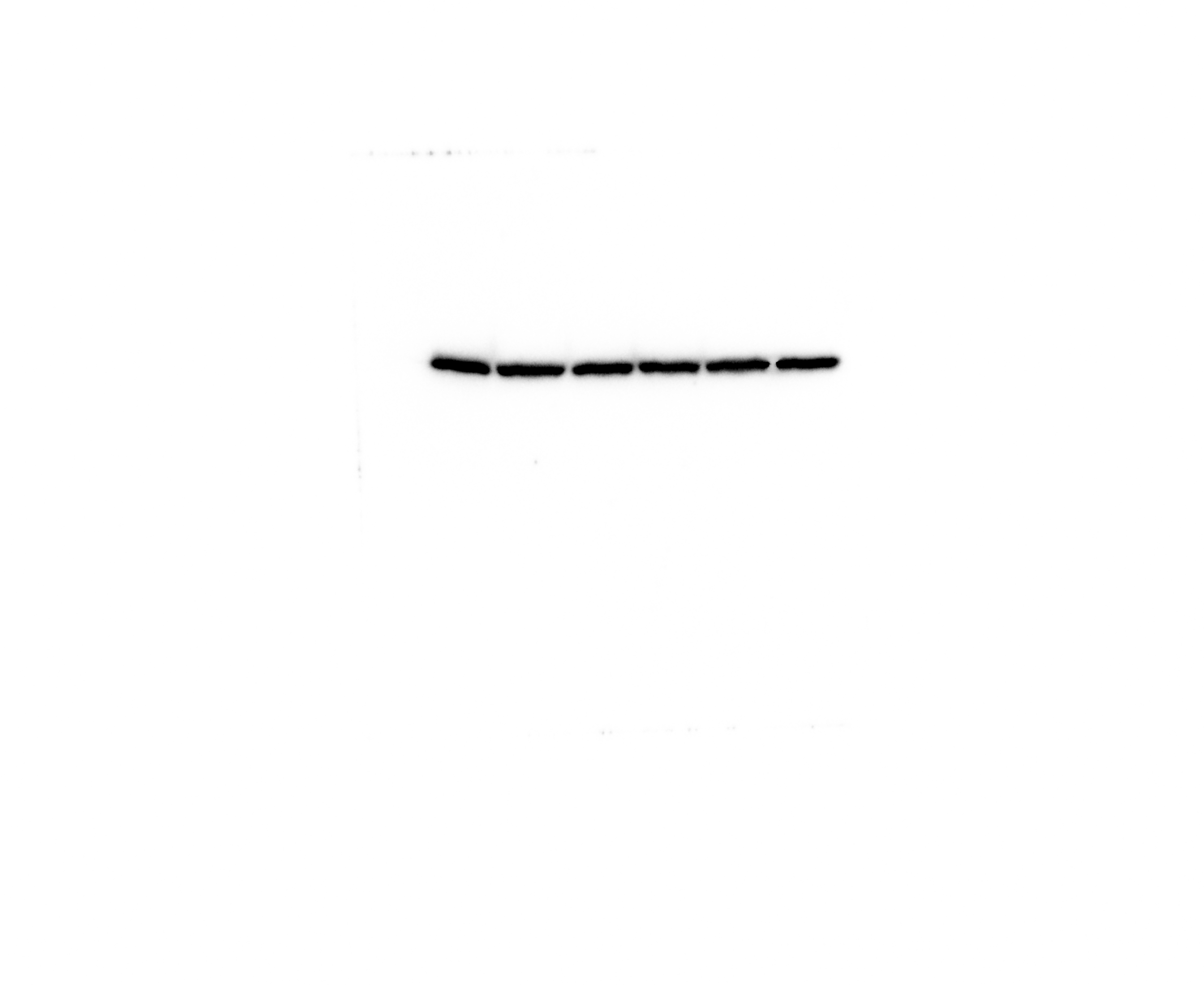

Supplement: Supplementary file 2 [file DataSheet1.ZIP › Original Gel Pictures/Figure 6 I/p65-replicate 3.tif]

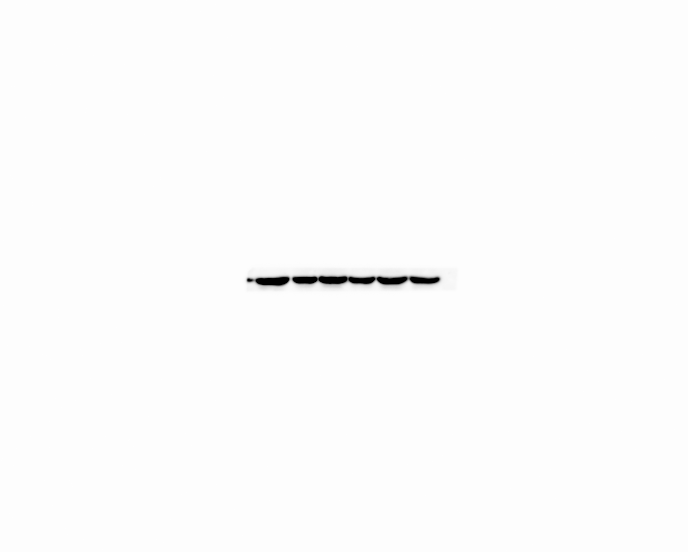

Supplement: Supplementary file 2 [file DataSheet1.ZIP › Original Gel Pictures/Figure 6 I/╬▓-actin-replicate 1.tif]

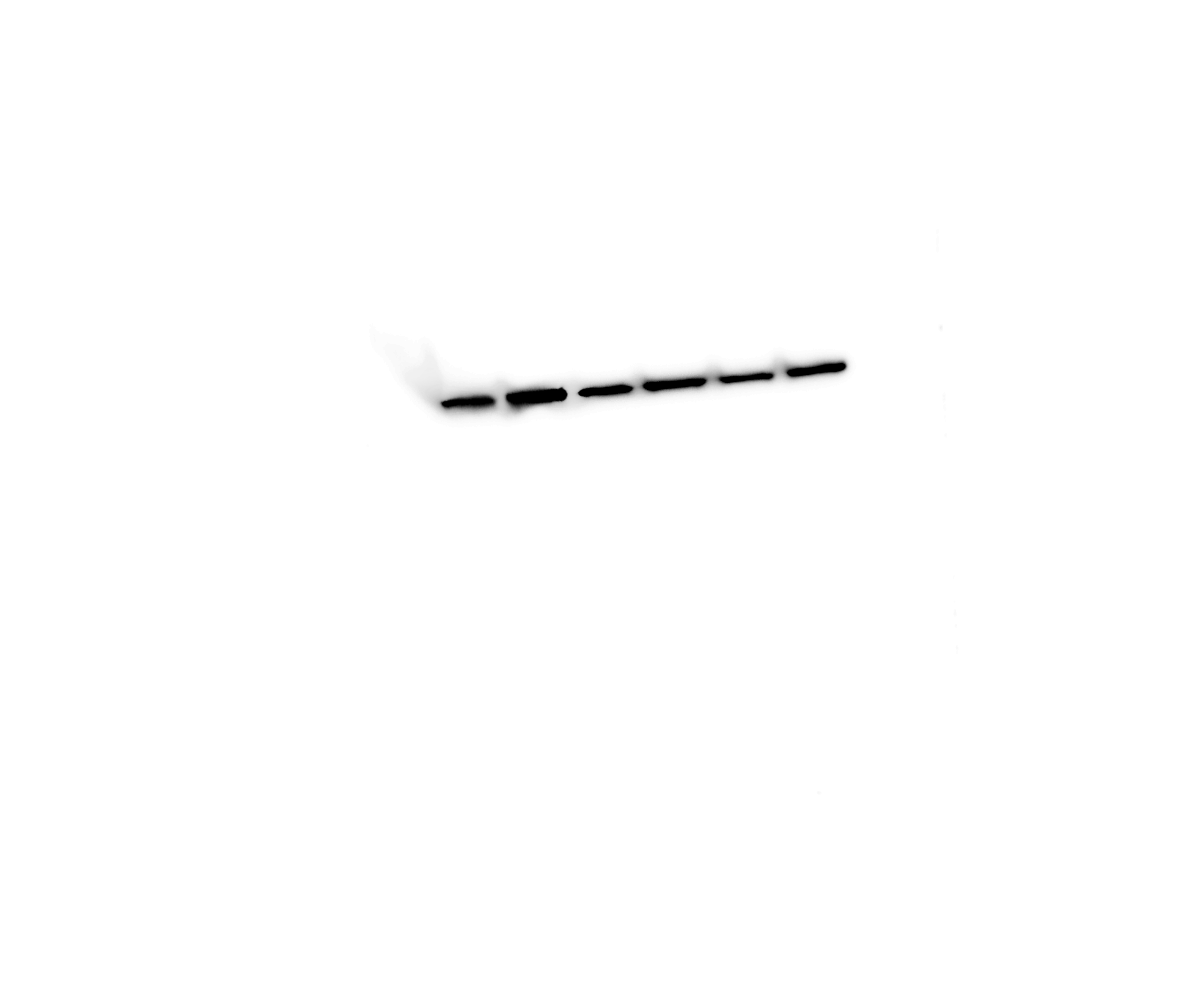

Supplement: Supplementary file 2 [file DataSheet1.ZIP › Original Gel Pictures/Figure 6 I/p-p65-replicate 2.tif]

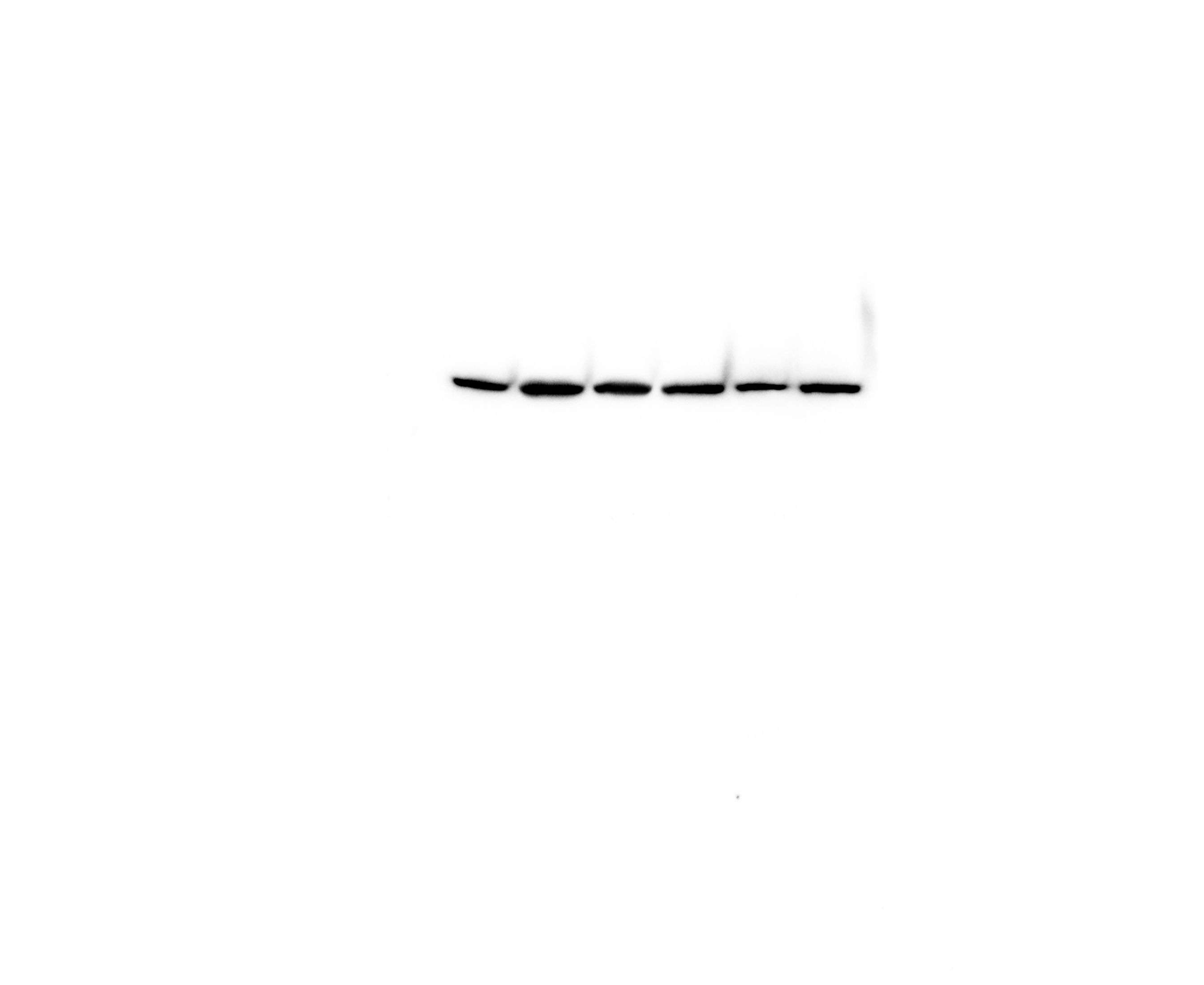

Supplement: Supplementary file 2 [file DataSheet1.ZIP › Original Gel Pictures/Figure 6 I/p-p65-replicate 3.tif]

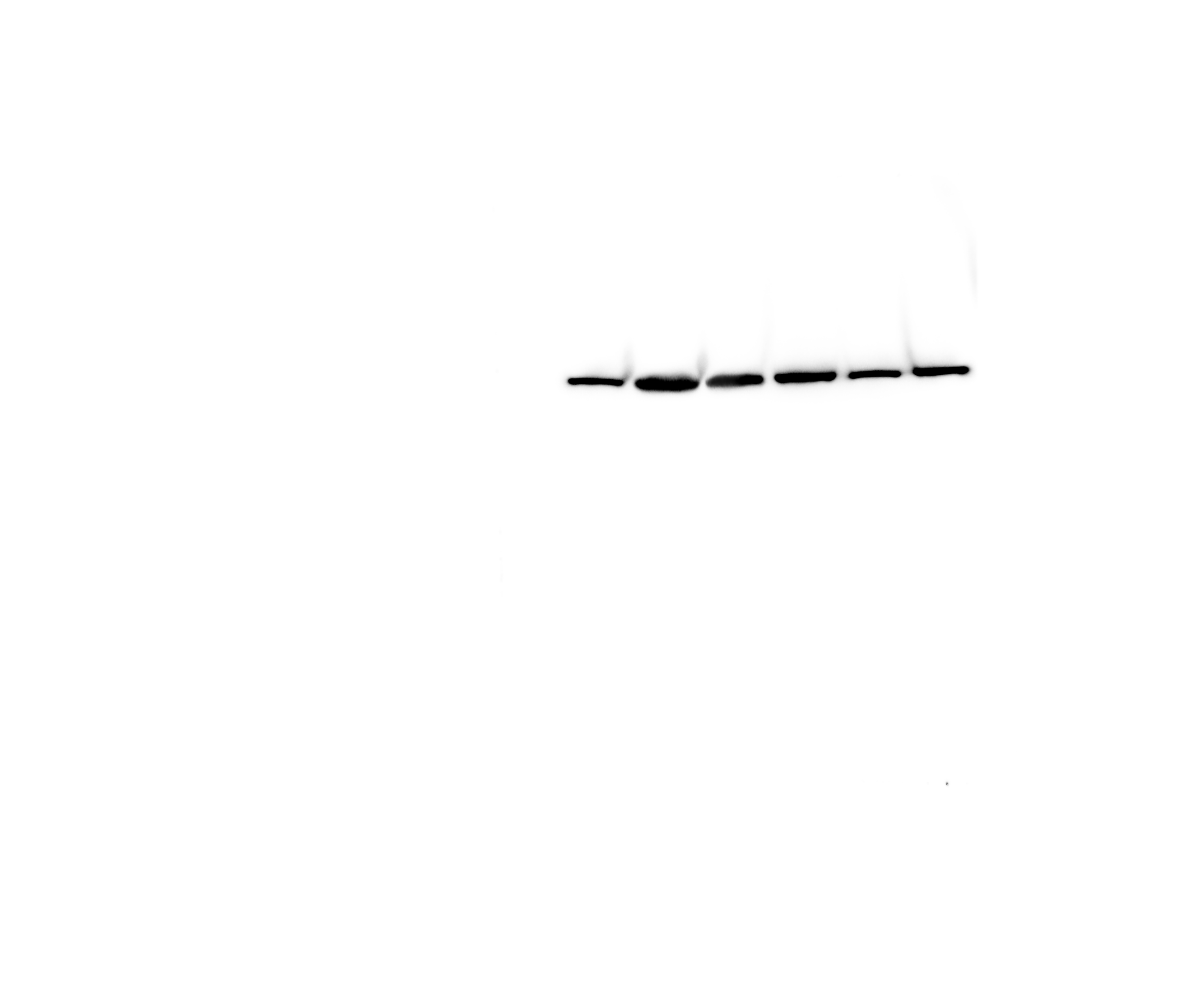

Supplement: Supplementary file 2 [file DataSheet1.ZIP › Original Gel Pictures/Figure 6 I/p-p65-replicate 1.tif]

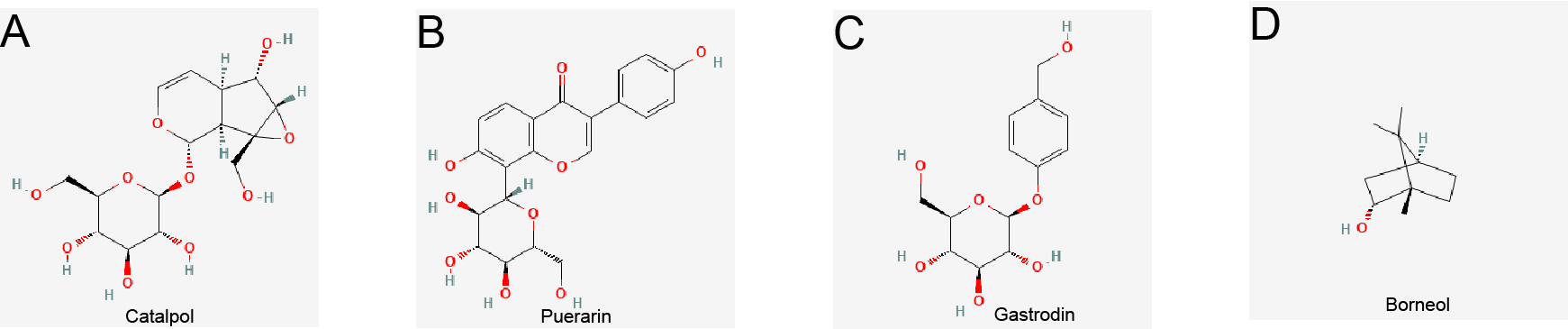

Supplement: Supplementary file 4 [file Image1.TIF]
