# Supplementary material for: Combined administration of catalpol, puerarin, gastrodin, and borneol modulates the Tlr4/Myd88/NF-κB signaling pathway and alleviates microglia inflammation in Alzheimer’s disease
Source: Front Pharmacol. 2024 Oct 31;15:1492237. doi: 10.3389/fphar.2024.1492237 (PMC11560463; doi:10.3389/fphar.2024.1492237)
Supplement: Supplementary file 6 [file Table2.DOCX]

| **Antibody Name** | **Dilution** | **Manufacturer** | **Cat No** |
| --- | --- | --- | --- |
| Rabbit anti-NeuN | 1:200 | Proteintech Biotechnology, Wuhan, China | 26975-1-AP |
| Rabbit anti-Synaptophysin | 1:1000 | Proteintech Biotechnology, Wuhan, China | 17785-1-AP |
| Rabbit anti-Aβ1-16 | 1:200 | Biolegend, San Diego, CA, USA | 805707 |
| Rabbit anti-APP | 1:1000 | Proteintech Biotechnology, Wuhan, China | 2254-1-AP |
| Mouse anti-Tau | 1:1000 | Proteintech Biotechnology, Wuhan, China | 66499-1-Ig |
| Rabbit anti-pTau396 | 1:1000 | Proteintech Biotechnology, Wuhan, China | BS4196 |
| Rabbit anti-GFAP | 1:200 | Proteintech Biotechnology, Wuhan, China | 16825-1-AP |
| Rabbit anti-Iba-1 | 1:200 | Proteintech Biotechnology, Wuhan, China | 10904-1-AP |
| Rabbit anti-TLR4 | 1:500/200 | Bioworld Technology, St. Paul, MN, USA | BS3489 |
| Rabbit anti-Myd88 | 1:1000/200 | Proteintech Biotechnology, Wuhan, China | 23230-1-AP |
| Rabbit anti-p65 | 1:1000/100 | Cell Signaling Technology, Danvers, MA, USA | 8242 |
| Rabbit anti-Tau | 1:100 | ABclonal, Wuhan, China | AP0053 |
| Mouse anti-β3-tubulin | 1:100 | Cell Signaling Technology, Danvers, MA, USA | 20536-1-AP |
| Donkey anti-rabbit, mouse | 1:200 | Jackson, West Grove, PA, USA | ab150077 |
| Goat anti-rabbit, mouse | 1:10000 | Jackson, West Grove, PA, USA | 111-035-003 |
| Rabbit anti-pTua231 | 1:10000 | ABclonal, Wuhan, China | AP0053 |
