# Supplementary material for: Combined administration of catalpol, puerarin, gastrodin, and borneol modulates the Tlr4/Myd88/NF-κB signaling pathway and alleviates microglia inflammation in Alzheimer’s disease
Source: Front Pharmacol. 2024 Oct 31;15:1492237. doi: 10.3389/fphar.2024.1492237 (PMC11560463; doi:10.3389/fphar.2024.1492237)
Supplement: Supplementary file 8 [file Table3.DOCX]

| **Sample Name** | **Sequencing Type** | **Orientation** | **Raw reads (Mb)** | **Raw Bases (Gb)** | **Q20 Ratio (%)** |
| --- | --- | --- | --- | --- | --- |
| Sham-1 | mRNA | Forward/Reverse | 78.37 | 11.76 | 98.26% |
| Sham-2 | mRNA | Forward/Reverse | 71.08 | 10.66 | 98.24% |
| Sham-3 | mRNA | Forward/Reverse | 76.14 | 11.42 | 98.27% |
| Sham-4 | mRNA | Forward/Reverse | 81.00 | 12.15 | 98.25% |
| Model-1 | mRNA | Forward/Reverse | 72.06 | 10.81 | 98.27% |
| Model-2 | mRNA | Forward/Reverse | 69.24 | 10.39 | 97.79% |
| Model-3 | mRNA | Forward/Reverse | 88.41 | 13.26 | 98.11% |
| Model-4 | mRNA | Forward/Reverse | 76.30 | 11.44 | 98.13% |
| CPGB-1 | mRNA | Forward/Reverse | 82.14 | 12.32 | 98.09% |
| CPGB-2 | mRNA | Forward/Reverse | 75.90 | 11.39 | 98.22% |
| CPGB-3 | mRNA | Forward/Reverse | 82.69 | 12.40 | 98.19% |
| CPGB-4 | mRNA | Forward/Reverse | 92.83 | 13.93 | 98.06% |
